# Serum vascular endothelial growth factor affects tissue fluid accumulation and is associated with deteriorating tissue perfusion and oxygenation in severe sepsis: a prospective observational study

**DOI:** 10.1186/s40001-023-01119-1

**Published:** 2023-04-21

**Authors:** Chin-Kuo Lin, Ying-Huang Tsai, Kuo-Chin Kao, Chieh-Mo Lin, Shao-Kui Zhou, Meng-Chin Ho, Shu-Yi Huang, Yu-Hung Fang, Che-Chia Chang, Wei-Chun Lee, Yueh-Lin Lee, Min-Chi Chen, Meng-Jer Hsieh, Yu-Ching Lin, Ming-Szu Hung, Wen-Chun Kuo, Bor-Shyh Lin

**Affiliations:** 1 Department of Pulmonary and Critical Care Medicine, Chang Gung Memorial Hospital, No. 6, West Sec. Chiapu Rd., Putzu City, Chiayi County 61363 Taiwan; 2 Graduate Institute of Clinical Medicine Sciences, College of Medicine, Chang Gung University, No. 259, Wenhua 1st Rd., Guishan Dist., Taoyuan, 33302 Taiwan; 3 Department of Pulmonary and Critical Care Medicine, Chang Gung Memorial Hospital, No. 5, Fuxing St., Guishan Dist., Linkou, Taoyuan City, 333 Taiwan; 4 Department of Respiratory Therapy, Chang Gung University College of Medicine, Taoyuan, 33302 Taiwan; 5 Department of Respiratory Therapy, Chang Gung Memorial Hospital, Chang Gung University College of Medicine, Taoyuan, 33305 Taiwan; 6 Chang Gung University of Science and Technology, No. 2, West Sec. Jiapu Rd., Puzi City, Chiayi County 61363 Taiwan; 7 Institute of Imaging and Biomedical Photonics, National Yang Ming Chiao Tung University, No. 301, Gaotie 3Rd Road, Guiren Dist., Tainan City, 71150 Taiwan; 8 Department of Public Health, Biostatistics Consulting Center, College of Medicine, Chang Gung University, No. 259, Wenhua 1st Road, Guishan Dist., Guishan, Taoyuan City, 33302 Taiwan; 9 Department of Hematology and Oncology, Chang Gung Memorial Hospital, No. 6, West Sec. Chiapu Rd, Putzu City, Chiayi County 61363 Taiwan; 10 Department of Respiratory Care, Chang Gung Memorial Hospital, No. 6, West Sec. Chiapu Rd, Putzu City, Chiayi County 61363 Taiwan; 11 Department of Medicine, College of Medicine, Chang Gung University, No. 259, Wenhua 1st Road, Guishan Dist., Taoyuan City, 33302 Taiwan

**Keywords:** Sepsis, Microcirculation, Tissue fluid, Near-infrared spectroscopy, Vascular endothelial growth factor

## Abstract

**Background:**

Positive fluid balance and tissue fluid accumulation are associated with adverse outcomes in sepsis. Vascular endothelial growth factor (VEGF) increases in sepsis, promotes vascular permeability, and may affect tissue fluid accumulation and oxygenation. We used near-infrared spectroscopy (NIRS) to estimate tissue hemoglobin (Hb) oxygenation and water (H_2_O) levels to investigate their relationship with serum VEGF levels.

**Material and methods:**

New-onset severe sepsis patients admitted to the intensive care unit were enrolled. Relative tissue concentrations of oxy-Hb (*[HbO*_*2*_*]*), deoxy-Hb (*[HbR]*), total Hb (*[HbT]*), and H_2_O (*[H*_*2*_*O]*) were estimated by near-infrared spectroscopy (NIRS) for three consecutive days and serum VEGF levels were measured. Comparisons between oliguric and non-oliguric patients were conducted and the correlations between variables were analyzed.

**Results:**

Among 75 eligible patients, compared with non-oliguric patients, oliguric patients were administrated more intravascular fluids (median [IQR], 1926.00 [1348.50–3092.00] mL/day vs. 1069.00 [722.00–1486.75] mL/day, *p* < 0.001) and had more positive daily net intake and output (mean [SD], 1,235.06 [1303.14] mL/day vs. 313.17 [744.75] mL/day, *p* = 0.012), lower *[HbO*_*2*_*]* and *[HbT]* over the three-day measurement (analyzed by GEE* p* = 0.01 and 0.043, respectively) and significantly higher *[H*_*2*_*O]* on the third day than on the first two days (analyzed by GEE *p* = 0.034 and 0.018, respectively). Overall, serum VEGF levels were significantly negatively correlated with *[HbO*_*2*_*]* and *[HbT]* (rho = − 0.246 and − 0.266, *p* = 0.042 and 0.027, respectively) but positively correlated with *[H*_*2*_*O]* (rho = 0.449, *p* < 0.001). Subgroup analysis revealed a significant correlation between serum VEGF and [H2O] in oliguric patients (rho = 0.532, *p* = 0.003). Multiple regression analysis determined the independent effect of serum VEGF on *[H*_*2*_*O]* (standardized coefficient = 0.281, *p* = 0.038).

**Conclusions:**

In severe sepsis, oliguria relates to higher positive fluid balance, lower tissue perfusion and oxygenation, and progressive tissue fluid accumulation. Elevated serum VEGF is associated with worsening tissue perfusion and oxygenation and independently affects tissue fluid accumulation.

**Supplementary Information:**

The online version contains supplementary material available at 10.1186/s40001-023-01119-1.

## Introduction

Sepsis caused by microbial infection is characterized by systemic inflammation, tissue hypoperfusion, and organ dysfunction and is associated with high mortality [1, 2]. Early fluid resuscitation is recommended to normalize systemic hemodynamics and improve sepsis-induced tissue hypoperfusion [3]. However, sepsis alters microcirculation and decouples microcirculation from macrocirculation [4]. Achieving systemic hemodynamic goals may not guarantee improved peripheral tissue perfusion and oxygenation [4–6]. Furthermore, infusion after initial resuscitation increases the risk of tissue edema and worsens tissue perfusion [7]. A higher cumulative fluid balance is independently associated with sepsis mortality [8], particularly in patients with acute renal failure (AKI) [9].

Systemic inflammation in sepsis disrupts endothelial function, leading to increased vascular permeability and tissue edema [10]. Serum vascular endothelial growth factor (VEGF) levels increase in early septic shock and contribute to increased vascular permeability [11]. VEGF is also related to increased pulmonary vascular permeability and edema in sepsis-induced acute respiratory distress syndrome (ARDS) and is associated with poor clinical outcomes [12, 13]. However, the relationship between serum VEGF levels and tissue perfusion, oxygenation, and edema in sepsis is unexplored.

Near-infrared spectroscopy (NIRS) can be used to determine the concentration and oxygenation status of light-absorbing chromophores in tissues and non-invasive assessment of microcirculation by measuring tissue oxygenation [14, 15]. Under different spectra of near-infrared wavelength light, hemoglobin (Hb) in different oxygenation states, such as oxy-Hb (HbO_2_) and deoxy-Hb (HbR), has differential absorption properties [16]. Light absorption is primarily derived from Hb, and depends on limb perfusion [17]. Tissue Hb oxygen saturation (StO_2_) can be obtained by estimating HbO_2_ and HbR in the tissues [16]. Low StO_2_ during early resuscitation is related to poor outcomes, and dynamic changes in StO_2_ can be used to estimate the vascular response to ischemic challenges [18, 19]. However, increased vascular permeability and tissue edema due to sepsis-related endothelial dysfunction may affect StO_2_ assessment [20]. Our previous study has identified an inverse relationship between tissue water (H_2_O) and StO_2_ estimated using NIRS in patients with severe sepsis [21]. We hypothesized that increased serum VEGF in patients with sepsis leads to the accumulation of tissue fluid and is associated with tissue hypoperfusion. This study compared differences in tissue perfusion, oxygenation, and H_2_O between oliguria and non-oliguria patients and investigated their relationship with serum VEGF levels.

## Methods

### Near-infrared diffuse optical technique and wireless optical monitoring system

The design of the proposed wireless optical monitoring system was based on the diffuse optical technique, and mainly comprised an optical probe, wireless optical signal acquisition module, and back-end system platform [22]. When light penetrates through human tissue, some photons may be scattered or absorbed by human tissue, causing optical intensity attenuation of the optical density [23]. Different tissue components provide different absorption and scattering capabilities corresponding to different wavelengths. In general, red and near-infrared light can provide relatively low absorption and scattering properties for many human tissue components, and Hb and H_2_O are two major absorbers [24–26]. According to the difference between their absorption spectra, the relative tissue concentrations of HbO_2_ (*[HbO*_*2*_*]*), HbR (*[HbR]*), and H_2_O (*[H*_*2*_*O]*) can then be estimated from the multiwavelength optical density attenuation. The total Hb (*[HbT]*) can then be obtained by the sum of *[HbO*_*2*_*]* and *[HbR]*, and StO_2_ is defined as the proportion of *[HbO*_*2*_*]* to *[HbT]*.

### Study design and patients

This prospective observational study was approved by the Institutional Review Board of the Chang Gung Medical Foundation (approval no. 103-5357B) and was conducted in a 20-bed medical intensive care unit (ICU) of Chiayi Chang Gung Memorial Hospital from November 27, 2015, to April 30, 2019. Adult patients (aged ≥ 18 years) who were transferred to the ICU from the emergency department and admitted within 72 h for new-onset severe sepsis were enrolled. Most patients in this study received initial fluid resuscitation according to the Surviving Sepsis Campaign 2016 guidelines to normalize systemic hemodynamics prior to ICU admission [27], and subsequent fluid management was at the discretion of the ICU clinician. The detailed eligibility criteria were described in our previous report [21]. After signing the informed consent, the patients were assessed with a non-invasive wireless NIRS device, which is still investigational, for three consecutive days. Demographic and clinical data were recorded for all patients, including age, sex, etiology of severe sepsis, Acute Physiology and Chronic Health Evaluation II score (APACHE II) on admission, systemic hemodynamic parameters, fluid balance parameters including daily net intake and output (I/O), urine output (UO), and administered intravascular fluid (IVF) recorded on the day before the NIRS measurements, laboratory results, and ICU outcome. For comparison, we divided patients into an oliguric group with mean urine output of < 500 mL and a non-oliguric group with mean urine output of ≥ 500 mL during the three-day study period. Patients with missing or incomplete NIRS data were excluded from final analysis.

### Determination of serum VEGF levels

Serum VEGF concentrations of each blood sample obtained from the patients on the first day of the study were determined by enzyme-linked immunosorbent assay (ELISA) using a commercial kit (Sigma-Aldrich; product number RAB0507; lot number 1210F0196) that recognizes VEGF-A, including biologically active VEGF121 and VEGF165, and the minimum detectable dose of VEGF-A was 10 pg/mL. We followed the manufacturer’s instructions to assay all samples without dilution in duplicate. All assays were performed in 96-well plates (150 μL total volume). Absorbances at 450 nm using the EnSpire multimode plate reader (PerkinElmer, Waltham, MA, USA) were measured. Serum VEGF levels were expressed in pg/mL. The inter-and intra-assay coefficient of variation was < 10% and < 12%, respectively.

### Sample size calculation

The sample size estimate for the initial observational study was based on our previous study of comparisons of NIRS parameters between patients and controls [21].

### Statistical analysis

Continuous data were summarized as mean (standard deviation [SD]) and 95% confidence interval (CI) or median (interquartile range [IQR]), depending on the normality of distribution. Categorical data are presented as counts and percentages. Differences between groups were analyzed using a two-sample t-test, Mann–Whitney U test, Chi-square test, or Fisher’s exact test, as appropriate. To compare the NIRS parameters, which were repeatedly measured in individuals at specified time intervals, we used generalized estimating equations (GEEs) to assess the differences between groups and changes in the parameters at different time intervals. Pearson or Spearman correlation coefficients were applied to investigate pairwise relationships between continuous variables, and multiple regression was conducted to identify the independent factors affecting *[H*_*2*_*O]*. All statistical analyses were performed using SPSS version 22 (IBM Corp., Armonk, NY, USA). All tests were two-tailed, and p < 0.05 indicated significance.

## Results

### Patient characteristics

**Fig. 1:**
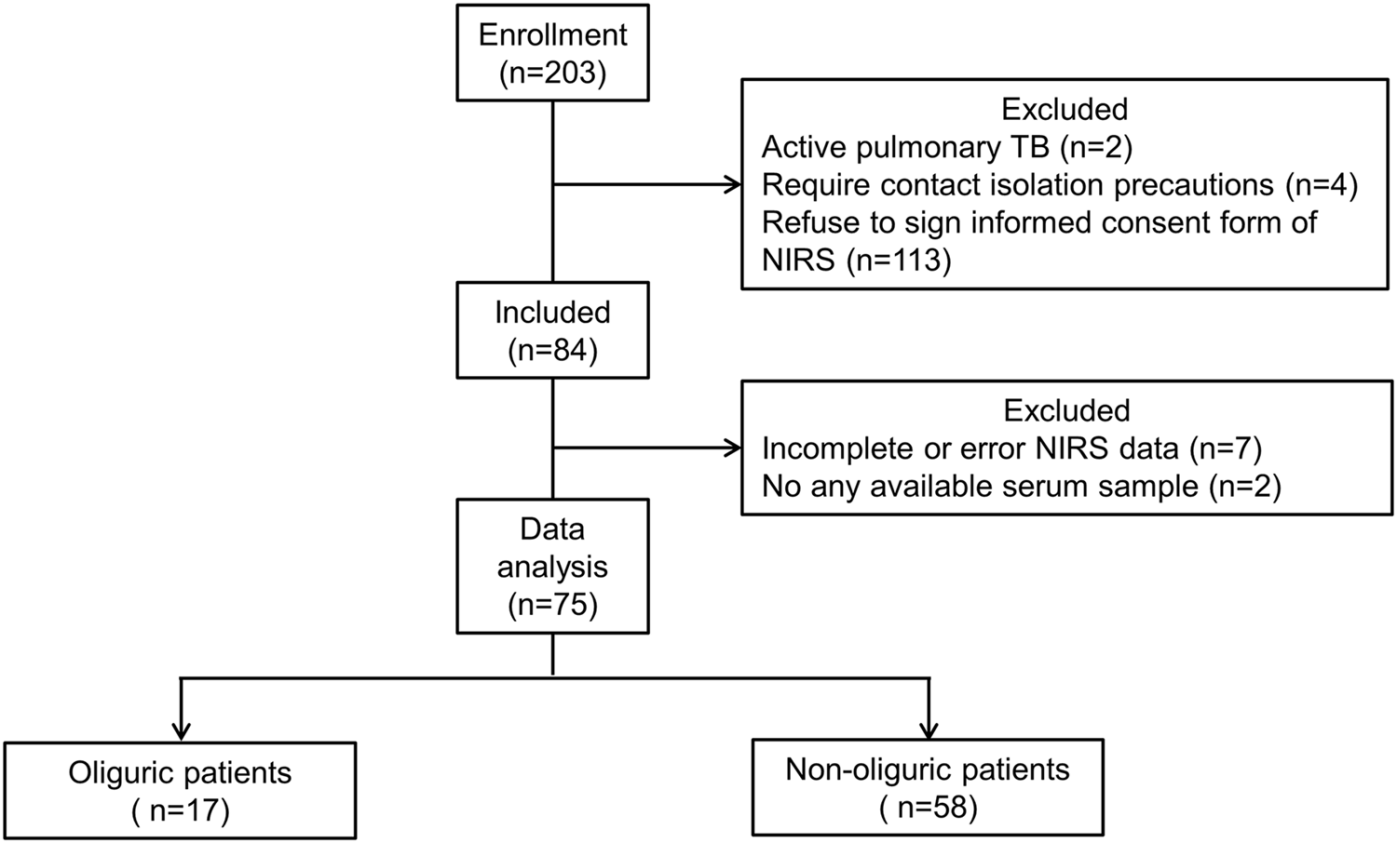
Flowchart of patient selection in the study. The patients included were ultimately divided into two groups, oliguric and non-oliguric, based on the average urine output during the three-day study period. Oliguric patients included those with less than 500 mL urine output, and non-oliguric patients included those with urine output of 500 mL or more. TB, tuberculosis; NIRS, near-infrared spectroscopy

A total of 203 patients were assessed for eligibility; 84 patients were included, and 75 patients with complete NIRS data and available serum samples for VEGF examination were included for data extraction and final data analysis (Fig. 1). Of these 75 patients, 17 had oliguria and 58 were non-oliguric (Table 1). Data are expressed as the mean (SD) or median (IQR). The median age was 77.00 (65.00–83.00) years. Additionally, 44 patients (59%) with septic shock received vasoactive agent therapy. Serum VEGF and albumin measured on the first day of the study were 122.17 (47.89–284.02) pg/mL and 2.87 (0.69) g/dL, respectively. During the three days, the total average of I/O was 522.55 (972.22) mL/day, of UO was 943.00 (547.00–1433.00) mL/day, and of administrated IVF was 1105.00 (767.00–1862.00) mL/day. Fifty-nine patients (79%) survived in the ICU.

**Table 1:** Demographic characteristics and clinical data of the patients with severe sepsis

Variables	Total (n = 75*)*	Oliguric patients (n = 17)	Non-oliguric patients (n = 58)	*p* value
Age, years, median (IQR)	77.00 (65.00–83.00)	77.00 (68.50–82.50)	76.00 (64.50–83.00)	0.709
Sex, male, n (%)	45 (60%)	11 (65%)	34 (59%)	0.781
Height, cm, mean (SD)	160.05 (7.78)	160.53 (8.63)	159.91 (7.59)	0.776
Weight, kg, mean (SD)	58.03 (12.26)	61.18 (14.70)	57.10 (11.42)	0.231
Body mass index, mean (SD)	22.65 (4.56)	23.84 (6.01)	22.30 (4.04)	0.332
Septic shock treated using vasoactive agents, n (%)	44 (59%)	14 (82%)	30 (52%)	0.028
Glasgow coma scale, median (IQR)	8.00 (6.00–15.00)	6.00 (3.00–10.00)	9.00 (7.00–15.00)	0.001
Acute Physiology and Chronic Health Evaluation Score II, mean (SD)	18.89 (6.67)	25.76 (6.09)	16.88 (5.39)	< 0.001
Mean arterial pressure, mmHg, median (IQR)	87.00 (78.00–94.50)	87.00 (77.75–97.75)	87.25 (77.75–94.50)	0.790
Systolic arterial pressure, mmHg, mean (SD)	114.63 (19.54)	116.41 (18.82)	114.10 (19.87)	0.671
Diastolic arterial pressure, mmHg, median (IQR)	59.00 (53.00–70.00)	62.00 (51.00–70.50)	58.00 (53.75–70.00)	0.830
White blood cells, 1000/µL, mean (SD)	13.54 (8.65)	15.41 (9.00)	12.99 (8.55)	0.315
Hemoglobin, g/dL, mean (SD)	11.16 (2.64)	10.37 (2.55)	11.39 (2.64)	0.162
Creatinine, mg/dL, median (IQR)	1.67 (1.02–2.49)	2.41 (1.81–3.64)	1.44 (0.96–2.11)	0.008
Arterial lactate, mg/dL (n = 73)^a^, median (IQR)	19.40 (13.60–30.50)	34.20 (17.50–77.65)	18.25 (13.38–24.78)	0.003
Vascular endothelial growth factor, pg/mL (n = 69)^a^, median (IQR)	122.17 (47.89–284.02)	99.65 (37.34–317.84)	141.86 (51.55–283.38)	0.590
Albumin, g/dL(n = 66)^a^, mean (SD)	2.87 (0.69)	2.95 (0.69)	2.85 (0.70)	0.666
Partial pressure of oxygen, mmHg, median (IQR)	104.50 (82.70–141.20)	98.70 (83.30–139.25)	106.10 (82.53–146.23)	0.617
Arterial oxygen saturation, percent, median (IQR)	98.00 (96.80–99.30)	97.90 (96.00–98.85)	98.25 (97.25–99.40)	0.182
Intake and Output, mL/day^b^				
Day 1 (n = 68)^a^, median (IQR)	201.00 (− 298.75–1190.25)	1142.00 (147.25–1729.00)	155.00 (− 384.25–834.50)	0.018
Day 2 (n = 73)^a^, median (IQR)	531.00 (− 132.00–1476.00)	1872.00 (135.00–2398.50)	461.00 (− 187.50–1080.00)	0.008
Day 3 (n = 62)^a^, mean (SD)	209.27 (1116.02)	397.45 (1475.58)	168.69 (1036.61)	0.542
Total average, mean (SD)	522.55 (972.22)	1235.06 (1303.14)	313.17 (744.75)	0.012
Urine output, mL/day^b^				
Day 1 (n = 68)^a^, median (IQR)	600.00 (192.50–1187.50)	70.00 (0.00–237.50)	880.00 (400.00–1312.50)	< 0.001
Day 2 (n = 73)^a^, median (IQR)	880.00 (460.00–1565.00)	120.00 (0.00–317.50)	1150.00 (800.00–1760.00)	< 0.001
Day 3 (n = 62)^a^, median (IQR)	1150.00 (690.00–1900.00)	50.00 (0.00–320.00)	1330.00 (940.00–1950.00)	< 0.001
Total average, median (IQR)	943.00 (547.00–1433.00)	100.00 (1.50–306.00)	1105.00 (854.25–1585.75)	< 0.001
Administered intravascular fluid, mL/day^b^				
Day 1 (n = 68)^a^, median (IQR)	973.00 (570.00–1620.25)	1726.50 (919.75–2441.50)	922.50 (526.00–1417.50)	0.018
Day 2 (n = 73)^a^, median (IQR)	1440.00 (902.00–2017.00)	2074.00 (1412.50–3575.00)	1350.00 (784.50–1907.00)	0.002
Day 3 (n = 62)^a^, median (IQR)	960.00 (480.00–1624.25)	1693.00 (624.00–2166.00)	940.00 (450.00–1420.00)	0.047
Total average, median (IQR)	1105.00 (767.00–1862.00)	1926.00 (1348.50–3092.00)	1069.00 (722.00–1486.75)	< 0.001
Diagnosis				
Pulmonary infection, n (%)	49 (65%)	14 (82%)	35 (60%)	0.147
Urinary tract infection, n (%)	35 (47%)	8 (47%)	27 (47%)	1.000
Hepatic or biliary tract infection, n (%)	8 (11%)	0 (0%)	8 (14%)	0.186
Spontaneous bacteria peritonitis, n (%)	1 (1%)	0 (0%)	1 (2%)	1.000
Pelvic infection, n (%)	1 (1%)	0 (0%)	1 (2%)	1.000
Cellulitis, n (%)	4 (5%)	1 (6%)	3 (5%)	1.000
Other, n (%)	3 (4%)	0 (0%)	3 (5%)	1.000
ICU survivor, n (%)	59 (79%)	8 (47%)	51 (88%)	0.001
ICU length of stay, days, median (IQR)	7.00 (4.00–11.00)	9.00 (4.00–14.50)	6.00 (4.75–10.25)	0.589

n: count; IQR: interquartile range; SD: standard deviation; ICU: intensive care unit

^a^Variable with missing or unrecorded data

^b^Data recorded on the day before the NIRS measurements

### Higher disease severity, higher accumulative fluid balance, and poor outcomes in oliguric patients

Compared with non-oliguric patients, oliguric patients had a higher rate of septic shock (82% vs. 52%, *p* = 0.028, Table 1), lower Glasgow coma scale (6.00 [3.00–10.00] vs. 9.00 [7.00–15.00], *p* = 0.001, Table 1), higher APACHE II (25.76 [6.09] vs. 16.88 [5.39], *p* < 0.001, Table 1), higher level of arterial lactate (34.20 [17.50–77.65] mg/dL vs. 18.25 [13.38–24.78] mg/dL, *p* = 0.003, Table 1), and more positive I/O (1235.06 [1303.14] mL/day vs. 313.17 [744.75] mL/day, *p* = 0.012; Table 1). Additionally, they received more IVFs (1926.00 [1348.50–3092.00] mL/day vs. 1069.00 [722.00–1486.75] mL/day, *p* < 0.001, Table 1) and had a lower ICU survival rate (47% vs. 88%, *p* < 0.001, Table 1). However, the post-resuscitation mean arterial pressure and serum VEGF and albumin levels were not significantly different (87.00 [77.75–97.75] mmHg vs. 87.25 [77.75–94.50] mmHg, *p* = 0.790; 99.65 [37.34–317.84] mg/dL vs. 141.86 [51.55–283.38] mg/dL, *p* = 0.590; and 2.95 [0.69] g/dL vs. 2.85 [0.70] g/dL, *p* = 0.666, respectively, Table 1).

### Lower tissue hemoglobin and oxy-hemoglobin and progressive increase of tissue water in oliguric patients

**Fig. 2:**
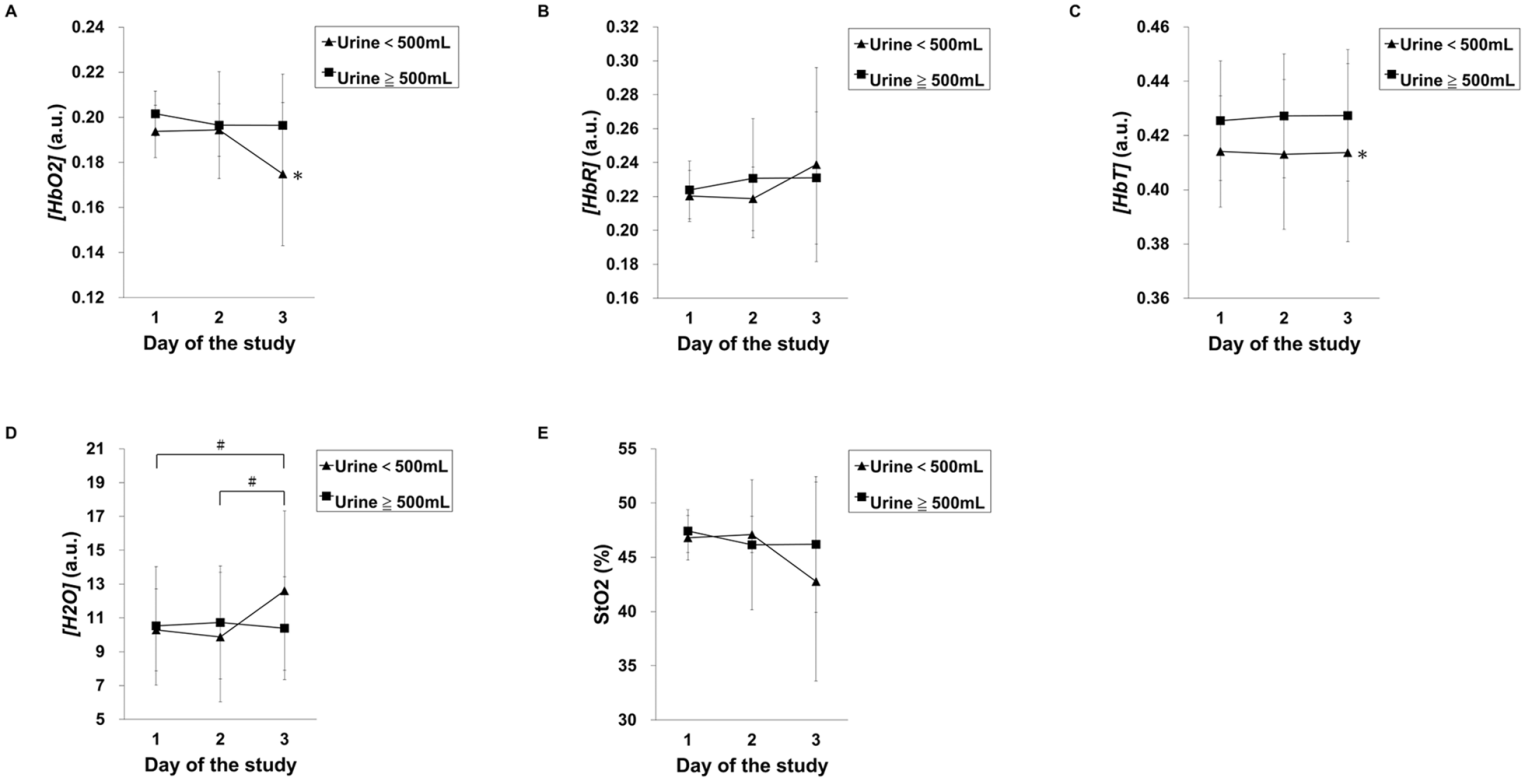
Comparison of time courses of relative tissue concentrations of hemoglobin, water, and tissue oxygen saturation between oliguric and non-oliguric patients. The relative tissue concentrations of **A** oxy-hemoglobin (*[HbO*_*2*_*]*), **B** deoxy-hemoglobin (*[HbR*]), **C** total hemoglobin *[HbT]*), and **D** H_2_O (*[H*_*2*_*O]*), and **E** tissue Hb oxygen saturation (StO_2_) measured from days 1 to 3 of the study in oliguric and non-oliguric patients are shown. Error bars represent standard deviation of the mean. Relative tissue concentrations of substances in arbitrary units (a.u.). * Generalized estimating equation (GEE) analysis showed a significant difference between the two groups (*p* ≤ 0.001). ^#^ GEE analysis showed that the parameters of oliguric patients changed significantly over time (*p* < 0.05). The actual *p* values are included in the Additional file 1

Regarding NIRS parameters, the mean (SD; 95% CI) of the anterior tibial *[HbO*_*2*_*]*, *[HbR]*, *[HbT]*, StO_2_, and *[H*_*2*_*O]* of oliguric and non-oliguric patients on the first day were 0.194 (0.012; 0.188–0.200) and 0.202 (0.010; 0.199–0.204), 0.220 (0.015; 0.213–0.228) and 0.224 (0.017; 0.219–0.228), 0.414 (0.020; 0.404–0.425) and 0.425 (0.022; 0.420–0.431), 46.80 (2.06; 45.74–47.86)% and 47.42 (1.96; 46.90–47.93)%, and 10.29 (2.43; 9.04–11.54) and 10.53 (3.50; 9.60–11.45), respectively (Table 2). On the third day, *[H*_*2*_*O]* in oliguric patients increased to 12.61 (4.7; 9.45–15.78), and *[HbO*_*2*_*]* and StO_2_ decreased to 0.175 (0.032; 0.153–0.196) and 42.77 (9.15; 36.62–48.92), respectively. GEE analysis showed that *[HbO*_*2*_*]* and *[HbT]* were significantly lower in oliguric patients than in non-oliguric patients during the three-day measurement (*p* = 0.01, p = 0.043, respectively; Fig. 2 and Additional file 1). Moreover, in oliguric patients, the *[H*_*2*_*O]* levels measured on the third day were significantly higher than on the first day and secondary day (*p* = 0.034 and 0.018, respectively; Fig. 2 and Additional file 1).

**Table 2:** Comparisons of the relative tissue concentrations of hemoglobin and water and tissue hemoglobin oxygen saturation between oliguric and non-oliguric patients

Measures	Days	Oliguric patients			Non-oliguric patients		
		Mean (SD)	95% CI	n	Mean (SD)	95% CI	n
*[HbO*_*2*_*]* (a.u)	1	0.194 (0.012)	0.188–0.200	17	0.202 (0.010)	0.199–0.204	58
	2	0.194 (0.012)	0.188–0.200	17	0.196 (0.024)	0.190–0.203	56
	3	0.175 (0.032)	0.153–0.196	11	0.196 (0.023)	0.190–0.203	51
*[HbR]* (a.u)	1	0.220 (0.015)	0.213–0.228	17	0.224 (0.017)	0.219–0.228	58
	2	0.219 (0.019)	0.209–0.228	17	0.231 (0.035)	0.221–0.240	56
	3	0.239 (0.057)	0.200–0.277	11	0.231 (0.039)	0.220–0.242	51
*[HbT]* (a.u)	1	0.414 (0.020)	0.404–0.425	17	0.425 (0.022)	0.420–0.431	58
	2	0.413 (0.028)	0.399–0.427	17	0.427 (0.023)	0.421–0.433	56
	3	0.414 (0.033)	0.392–0.436	11	0.427 (0.024)	0.421–0.434	51
StO_2_ (%)	1	46.80 (2.06)	45.74–47.86	17	47.42 (1.96)	46.90–47.93	58
	2	47.10 (1.67)	46.24–47.96	17	46.15 (6.00)	44.54–47.76	56
	3	42.77 (9.15)	36.62–48.92	11	46.20 (6.25)	44.44–47.95	51
*[H*_*2*_*O]* (a.u)	1	10.29 (2.43)	9.04–11.54	17	10.53 (3.50)	9.60–11.45	58
	2	9.87 (3.82)	7.90–11.84	17	10.73 (3.34)	9.83–11.62	56
	3	12.61 (4.71)	9.45–15.78	11	10.39 (3.04)	9.54–11.24	51

a.u., arbitrary unit; SD: standard deviation*;* CI: confidence interval; *[HbO*_*2*_*]*: relative tissue concentration of oxy-hemoglobin; *[HbR]*: relative tissue concentration of deoxy-hemoglobin; *[HbT]*: relative tissue concentration of total hemoglobin; StO_2_: tissue hemoglobin oxygen saturation; *[H*_*2*_*O]*: relative tissue concentration of H_2_O

### The relationship between tissue oxygenation and water content and fluid balance

I/O was negatively correlated with *[HbT]* (rho = − 0.268, *p* = 0.035; Table 3 and Additional file 1), and UO was positively correlated with *[HbO*_*2*_*]* and *[HbT]* (rho = 0.367 and 0.297, *p* = 0.003 and 0.019, respectively; Table 3 and Additional file 1) measured on the third day. Additionally, administered IVF was negatively correlated with *[HbT]* measured on the first day (rho = − 0.259,* p* = 0.033; Table 3 and Additional file 1). However, there was no association between the tissue H_2_O content and fluid balance parameters.

**Table 3:** Correlation coefficients between NIRS parameters and intake and output, urine output, administrated fluid, serum VEGF, and serum albumin

Measures	Days	*[H*_*2*_*O]*	*[HbO*_*2*_*]*	*[HbR]*	*[HbT]*	*StO*_*2*_
I/O^*a*^	1	− 0.050	− 0.139	− 0.142	− 0.223	0.012
	2	− 0.013	0.036	0.036	0.041	0.014
	3	0.046	− 0.140	− 0.166	− 0.268^c^	0.061
UO^a^	1	− 0.084	0.233	− 0.025	0.136	0.181
	2	0.098	0.204	0.156	0.194	0.038
	3	− 0.240	0.367^b^	0.136	0.297^*c*^	0.099
IVF^a^	1	0.010	− 0.143	− 0.204	− 0.259^*c*^	0.029
	2	0.077	− 0.021	0.059	0.043	− 0.080
	3	0.179	− 0.092	− 0.025	− 0.142	− 0.042
VEGF	1	0.449^b^	− 0.246^c^	− 0.105	− 0.266^*c*^	− 0.105
Albumin	1	− 0.329^b^	0.130	− 0.024	0.036	0.085

Numbers in cells are Pearson’s r or Spearman’s rho correlation coefficients. NIRS: near-infrared spectroscopy; VEGF: vascular endothelial growth factor; *[HbO*_*2*_*]*: relative tissue concentration of oxy-hemoglobin; *[HbR]*: relative tissue concentration of deoxy-hemoglobin; *[HbT]*: relative tissue concentration of total hemoglobin; StO_2_: tissue hemoglobin oxygen saturation; *[H*_*2*_*O]*: relative tissue concentration of H_2_O; I/O: intake and output; UO: urine output; IVF: intravascular fluid, ^a^Data recorded on the day before the NIRS measurements. ^b^p < 0.01; ^c^p < 0.05. The actual *p* values have been included in Additional file 1

### The relationship between tissue oxygenation and water content and serum VEGF and albumin

**Fig. 3:**
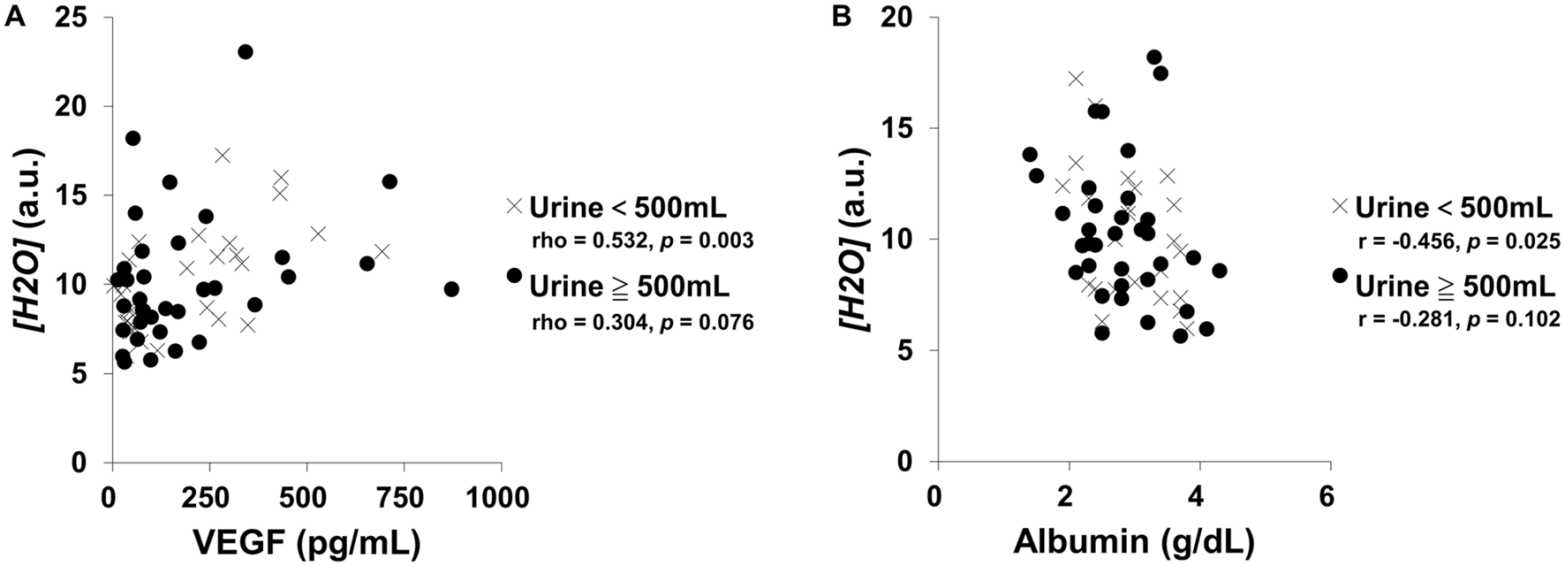
A graphical representation of the relationships between tissue water content and serum VEGF and albumin in oliguric and non-oliguric patients. Correlations between the regional tissue concentration of H_2_O (*[H*_*2*_*O]*) and **A** serum vascular endothelial growth factor (VEGF) and **B** serum albumin are shown. a.u., arbitrary unit

VEGF regulates VE-cadherin hyperphosphorylation at endothelial adherences junctions and affects vascular permeability [28]. Albumin is the most abundant circulating protein in the plasma and acts as the most significant modulator of plasma oncotic pressure [29]. Both affect tissue fluid balance and edema and may be related to tissue oxygenation. Therefore, we analyzed the correlations between NIRS parameters and serum VEGF and albumin levels. We found that there was a negative correlation between serum VEGF and *[HbO*_*2*_*]* and *[HbT]* (rho = − 0.246 and − 0.266, *p* = 0.042 and 0.027, respectively; Table 3 and Additional file 1), but a positive correlation between serum VEGF and *[H*_*2*_*O]* (rho = 0.449, *p* < 0.001; Table 3 and Additional file 1). In contrast, serum albumin was negatively correlated with *[H*_*2*_*O]* (r = − 0.329, *p* = 0.007; Table 3 and Additional file 1). However, there was no association between serum albumin level and tissue Hb oxygenation and content. In addition, subgroup analysis showed that serum VEGF, albumin, and *[H*_*2*_*O]* were significantly correlated in oliguric patients (rho = 0.532 and r = − 0.456, *p* = 0.003 and 0.025, respectively; Fig. 3) but not in non-oliguric patients (rho = 0.304 and r = − 0.281, *p* = 0.076 and 0.102, respectively; Fig. 3).

### Serum VEGF affects the increase in tissue water

To elucidate the independent factors affecting the increase in tissue water, we further analyzed the correlation between fluid balance parameters, serum VEGF, and albumin, and conducted a multiple linear regression analysis that included all the factors significantly correlated with *[H*_*2*_*O]*. We found that serum VEGF was negatively correlated with serum albumin (rho = − 0.401, *p* = 0.002; Table 4), but there was no correlation between serum VEGF and fluid balance parameters. Multiple linear regression revealed that serum VEGF levels were independently positively correlated to *[H*_*2*_*O]* (standardized coefficients = 0.281, *p* = 0.038; Table 5).

**Table 4:** Correlation coefficients between urine output, administrated fluid, serum VEGF, and serum albumin

Measures	I/O	UO	IV	VEGF	Albumin
I/O^a^	1				
UO^a^	− 0.390^b^	1			
IVF^a^	0.544^b^	− 0.043	1		
VEGF	0.093	0.011	− 0.033	1	
Albumin	0.107	− 0.143	0.052	− 0.401^b^	1

Numbers in cells are Pearson’s r or Spearman’s rho correlation coefficients. Vascular endothelial growth factor, VEGF; I/O: intake and output; UO: urine output; IVF: intravascular fluid. ^a^Data recorded on the day before the near-infrared spectroscopy measurements. ^b^p < 0.01. The actual *p* values have been included in Additional file 1

**Table 5:** Multiple linear regression analysis of the effect of serum VEGF and albumin on* [H*_*2*_*O]*

Model	B	Standard Error	Beta	t	*P* value	95% Confidence Interval for B		Tolerance	VIF
VEGF (pg/mL)	0.004	0.002	0.281	2.122	0.038	0 to 0.008		0.816	1.225
Albumin (g/dL)	− 0.971	0.567	− 0.227	− 1.714	0.092	− 2.106 to 0.164		0.816	1.225
	R^2^	adjusted R^2^	F	df	p value				
	0.185	0.156	6.468	2,57	0.003				

Vascular endothelial growth factor, VEGF; *[H*_*2*_*O]*, relative tissue concentration of H_2_O; Unstandardized coefficients, B; Standardized coefficients, Beta; Variance inflation factor, VIF; df, degrees of freedom

## Discussion

Septic shock is a distributive shock that involves abnormal microvascular blood flow [30]. Early fluid resuscitation can expand intravascular volume to improve tissue hypoperfusion and oxygenation and protect organ function [31–33]. However, excessive infusion may lead to increased fluid accumulation in the interstitial space, which may impede capillary blood flow, impair oxygen diffusion, distort tissue architecture, and adversely affect organ function [34]. Associations between positive fluid balance and deleterious outcomes and mortality in sepsis have been documented [35, 36]. In addition, positive fluid balance and oliguria are associated with increased mortality in patients with AKI, and the development of acute renal failure (ARF) in sepsis has further raised concerns about fluid therapy [9, 37]. Therefore, we divided the patients into oliguric and non-oliguric groups. We found a higher disease severity and rate of septic shock in oliguric patients than in non-oliguric patients. Furthermore, they received more IVF at the start of sepsis and had higher positive fluid balance and ICU mortality. These findings are consistent with Wim Van Biesen’s findings that septic patients with ARF had higher fluid loading during the first three days of sepsis [38]. Therefore, in septic patients with AFR, over-infusion and positive fluid balance are likely to occur in early sepsis and may associate with an increased risk of death. Thus, conservative fluid therapy is critical for dealing with unstable hemodynamics in sepsis patients with oliguria.

Understanding the state of tissue fluid is necessary to prevent the harm of fluid overload on microcirculation and may guide fluid therapy and prevent liberal fluid administration. To estimate the accumulated tissue fluid, we developed a NIRS device to detect tissue H_2_O and found that *[H*_*2*_*O]* was significantly higher in patients with severe sepsis than in healthy controls [21]. In the present research, we discovered that oliguric patients with severe sepsis had more positive fluid balance and gradually accumulated H_2_O in the tissues. Besides, their tissue HbO_2_ and HbT levels were lower, meaning their tissue perfusion and oxygenation were poorer than non-oliguric patients [39]. Meanwhile, inadequate tissue perfusion and oxygenation were related to a positive fluid balance and decreased UO, which became significant over time. Therefore, receiving more fluid to correct septic shock, followed by progressively accumulating tissue fluid and reduced tissue perfusion and oxygenation, may account for poor ICU outcomes in oliguric patients. Excess fluid can be removed by hemofiltration. Aggressive fluid removal by hemofiltration reduced cutaneous blood flow but did not change systemic hemodynamics in fluid-overload septic shock patients, reiterating the decoupling between macrocirculation and microcirculation in sepsis [40]. Correcting the microcirculation and tissue oxygenation is the ultimate goal of managing hemodynamic instability caused by sepsis, and comprehensively understanding the microcirculatory response to fluid therapy requires real-time tissue perfusion, oxygenation, and fluid monitoring. Simultaneously measuring tissue oxygenation and water content, NIRS may be a potentially powerful tool for assessing microcirculation and tissue fluid accumulation in patients with sepsis, especially those who have oliguria and require fluid therapy to correct unstable hemodynamics. Further studies are needed to validate its clinical application.

As a multifunctional cytokine, VEGF can promote angiogenesis, affect vascular permeability, and play a diverse role in tissue damage [41–43]. Decreased VEGF expression in the kidney is associated with glomerular endothelial injury and the development and progression of lipopolysaccharide(LPS)-induced AKI [44]. Karlsson et al. found that the median serum VEGF level in patients with severe sepsis was 423 pg/mL at the time of study entry and increased over the first 72 h [45]. Besides, low serum VEGF levels were associated with renal dysfunction and mortality. Compared with Karlsson et al.’s study, the patients in our study had relatively lower disease severity and serum VEGF levels. We found no significant differences in the serum VEGF levels between oliguric and non-oliguric patients. A systemic review and meta-analysis conducted by Tang et al. revealed that septic patients with high VEGF had poor clinical outcomes [13]. However, significant heterogeneity between the reviewed studies challenges the study’s conclusion. Controversial findings in different studies may arise from the disease severities of the patients studied and the time points at which VEGF was investigated during sepsis, which would yield different results and thus affect inferences. Besides, the effects of VEGF on different organs may vary. Therefore, comprehensive time-course studies on different severity of sepsis should be performed to elucidate the effects of VEGF on various organs at different disease stages.

Circulating VEGF and albumin are crucial factors in regulating tissue fluid balance. VEGF regulates vascular permeability and promotes the leakage of H_2_O from the capillaries to the interstitium. In contrast, albumin accounts for approximately 80% of the total plasma oncotic pressure, driving H_2_O from the interstitium into capillaries [29, 41, 46, 47]. Plasma VEGF obtained from patients with ARDS increases endothelial cell permeability-inducing activity and may be involved in developing ARDS pulmonary edema [12]. Furthermore, anti-VEGF antibodies inhibit LPS-induced vascular leakage in organ tissues, including the lungs, spleen, and kidneys [48]. Therefore, circulating VEGF may promote the accumulation of tissue fluid. Using NIRS to detect tissue H_2_O, we demonstrated that serum VEGF contributes to increased tissue fluid accumulation in the early stages of severe sepsis, especially in oliguric patients. Elevated serum VEGF levels are associated with poor tissue perfusion and oxygenation. Tissue hypoxia activates the hypoxia-inducible factor-1 pathway to promote VEGF production [49]. Accordingly, a vicious cycle may arise: sepsis-induced tissue hypoperfusion leads to tissue hypoxia and promotes the elevation of serum VEGF, which alters vascular permeability and tissue fluid accumulation and further deteriorates tissue perfusion and oxygenation. In contrast to VEGF, the decrease in serum albumin levels in sepsis causes tissue edema [50]. The present study demonstrated a correlation between tissue H_2_O and serum albumin levels. In septic resuscitation, volume expansion with crystalloid and saline alone results in a more positive fluid balance and tissue fluid accumulation, which is related to poor prognosis [8, 36]. On the contrary, resuscitation with albumin provides survival benefits [51]. This benefit may be due to less fluid accumulation during volume expansion with albumin, thereby preserving tissue perfusion and oxygenation. Moreover, decreased serum albumin levels in sepsis are associated with capillary leak syndrome caused by increased vascular permeability [52]. This study demonstrated a negative correlation between serum VEGF and albumin. It is speculated that serum VEGF mediates vascular permeability and strongly affects albumin retained in circulation. Thus, serum VEGF was independently associated with tissue fluid accumulation even after accounting for the effects of fluid balance parameters and serum albumin. To summarize the analysis and above discussion, serum VEGF is a crucial factor associated with fluid accumulation in tissue and may affect tissue perfusion and oxygenation. Measuring serum VEGF and estimating tissue fluid by NIRS are recommended in sepsis, which may help assess tissue fluid accumulation, especially when performing fluid therapy for oliguric patients, and facilitate a comprehensive assessment of the microcirculation.

This study has some limitations. First, oliguric patients had more severe sepsis and needed more fluids to correct septic shock, leading to a higher cumulative fluid balance. Meanwhile, they showed lower tissue perfusion and oxygenation. Thus, reduced tissue perfusion and oxygenation and a higher cumulative fluid balance may be accompanied by severe disease, forming a link between them. Further research is recommended to clarify the causal effect of positive fluid balance on tissue perfusion and oxygenation. Second, serum VEGF is related to tissue perfusion and oxygenation, but the independent role of serum VEGF remains to be elucidated. Regional arteriolar pressure and resistance critically affecting microcirculatory blood flow should be considered when exploring factors affecting tissue perfusion and oxygenation [53]. However, to our knowledge, there is currently no clinically available device to directly estimate the blood pressure and resistance of tissue vessels. Current clinical hemodynamic estimates are systemic. Measured systemic blood pressure and systemic vascular resistance calculated from systemic blood pressure and cardiac output represent systemic hemodynamics, which do not necessarily respond directly to regional tissue hemodynamics in sepsis. Third, theoretically, underlying comorbidities that alter peripheral vascular status may affect tissue perfusion [54], but we did not elucidate the influence of patients' underlying comorbidities on tissue perfusion in the present study. However, most previous studies on tissue perfusion investigated their relationship with systemic hemodynamics [40, 55], and the effect of a patient's underlying comorbidities on tissue perfusion has not been well established. Finally, this study's results are limited by the small sample size. Furthermore, since the patient population in this study was relatively old (median age 77.00 years), caution must be taken when extrapolating these results to younger populations. Nonetheless, these findings remain critical for identifying all possible factors affecting sepsis-associated tissue hypoperfusion and hypoxia, which may contribute to the development of personalized precision medicine. Further studies are warranted to elucidate the mechanisms underlying the effects of VEGF on microcirculation and tissue fluid in sepsis.

## Conclusion

In severe sepsis, oliguric patients have a more serious disease, higher cumulative fluid balance, lower tissue perfusion and oxygenation, and progressive tissue fluid accumulation. Increased serum VEGF levels in sepsis are related to tissue perfusion and oxygenation deterioration and independently affect increased tissue fluid. Serum VEGF measurement is recommended to understand the microcirculation and tissue fluid in sepsis, especially in oliguric patients.

## Supplementary Information

**Additional file 1.** The additional details describing the analyses of statistical data in the study.

## Data Availability

All data generated or analyzed during this study are included in this published article and the supplementary information. The datasets used and analyzed during the current study are available from the corresponding author on reasonable request.

## Abbreviations

AKI: Acute renal failure
VEGF: Vascular endothelial growth factor
ARDS: Acute respiratory distress syndrome
NIRS: Near-infrared spectroscopy
Hb: Hemoglobin
HbO_2_: Oxy-hemoglobin
HbR: Deoxy-hemoglobin
StO_2_: Tissue hemoglobin oxygen saturation
H_2_O: Water
*[HbO*_*2*_*]*: The relative tissue concentrations of oxy-hemoglobin
*[HbR]*: The relative tissue concentrations of deoxy-hemoglobin
*[H*_*2*_*O]*: The relative tissue concentrations of water
*[HbT]*: The relative tissue concentrations of total hemoglobin
ICU: Intensive care unit
APACHE II: Acute Physiology and Chronic Health Evaluation II score
I/O: Daily net intake and output
UO: Urine output
IVF: Intravascular fluid
ELISA: Enzyme-linked immunosorbent assay
SD: Standard deviation
CI: Confidence interval
IQR: Interquartile range
GEE: Generalized estimating equation
ARF: Acute renal failure
LPS: Lipopolysaccharide
