# Supplementary material for: Serum vascular endothelial growth factor affects tissue fluid accumulation and is associated with deteriorating tissue perfusion and oxygenation in severe sepsis: a prospective observational study
Source: Eur J Med Res. 2023 Apr 21;28:155. doi: 10.1186/s40001-023-01119-1 (PMC10120235; doi:10.1186/s40001-023-01119-1)
Supplement: Supplementary file 1 — Additional file 1. The additional details describing the analyses of statistical data in the study. [file 40001_2023_1119_MOESM1_ESM.docx]

***Additional file***

**1 Supplementary Data**

The additional details describing the analyses of statistical data in the study

**Content**

**1.1 Detailed analyses and results for Table 1 p. 2-82**

**1.2 Detailed analyses and results for Table 2 p.83-98**

**1.3 Detailed analyses and results for Table 3 p.99-108**

**1.4 Detailed analyses and results for Table 4 p.109-111**

**1.5 Detailed analyses and results for Figure 2 p.112-126**

**1.6 Detailed analyses and results for Figure 3 p.127-128**

**1.1 Detailed analyses and results for Table 1**

**Age**

| **Tests of Normality** | | | | | | |
| --- | --- | --- | --- | --- | --- | --- |
|  | Kolmogorov-Smirnov^a^ | | | Shapiro-Wilk | | |
|  | Statistic | df | Sig. | Statistic | df | Sig. |
| Age | .145 | 75 | .001 | .918 | 75 | .000 |
| a. Lilliefors Significance Correction | | | | | | |

Patients

| **Descriptives** | | | | |
| --- | --- | --- | --- | --- |
|  | | | Statistic | Std. Error |
| Age | Mean | | 73.03 | 1.493 |
|  | 95% Confidence Interval for Mean | Lower Bound | 70.05 |  |
|  |  | Upper Bound | 76.00 |  |
|  | 5% Trimmed Mean | | 73.93 |  |
|  | Median | | 77.00 |  |
|  | Variance | | 167.188 |  |
|  | Std. Deviation | | 12.930 |  |
|  | Minimum | | 38 |  |
|  | Maximum | | 91 |  |
|  | Range | | 53 |  |
|  | Interquartile Range | | 18 |  |
|  | Skewness | | -.920 | .277 |
|  | Kurtosis | | .369 | .548 |

| **Statistics** | | |
| --- | --- | --- |
| Age | | |
| N | Valid | 75 |
|  | Missing | 0 |
| Percentiles | 25 | 65.00 |
|  | 50 | 77.00 |
|  | 75 | 83.00 |

Oliguria

| **Descriptives** | | | | |
| --- | --- | --- | --- | --- |
|  | | | Statistic | Std. Error |
| Age | Mean | | 74.06 | 2.898 |
|  | 95% Confidence Interval for Mean | Lower Bound | 67.91 |  |
|  |  | Upper Bound | 80.20 |  |
|  | 5% Trimmed Mean | | 75.12 |  |
|  | Median | | 77.00 |  |
|  | Variance | | 142.809 |  |
|  | Std. Deviation | | 11.950 |  |
|  | Minimum | | 43 |  |
|  | Maximum | | 86 |  |
|  | Range | | 43 |  |
|  | Interquartile Range | | 14 |  |
|  | Skewness | | -1.513 | .550 |
|  | Kurtosis | | 2.060 | 1.063 |

| **Statistics** | | |
| --- | --- | --- |
| Age | | |
| N | Valid | 17 |
|  | Missing | 0 |
| Percentiles | 25 | 68.50 |
|  | 50 | 77.00 |
|  | 75 | 82.50 |

Non-oliguria

| **Descriptives** | | | | |
| --- | --- | --- | --- | --- |
|  | | | Statistic | Std. Error |
| Age | Mean | | 72.72 | 1.745 |
|  | 95% Confidence Interval for Mean | Lower Bound | 69.23 |  |
|  |  | Upper Bound | 76.22 |  |
|  | 5% Trimmed Mean | | 73.64 |  |
|  | Median | | 76.00 |  |
|  | Variance | | 176.554 |  |
|  | Std. Deviation | | 13.287 |  |
|  | Minimum | | 38 |  |
|  | Maximum | | 91 |  |
|  | Range | | 53 |  |
|  | Interquartile Range | | 19 |  |
|  | Skewness | | -.811 | .314 |
|  | Kurtosis | | .205 | .618 |

| **Statistics** | | |
| --- | --- | --- |
| Age | | |
| N | Valid | 58 |
|  | Missing | 0 |
| Percentiles | 25 | 64.50 |
|  | 50 | 76.00 |
|  | 75 | 83.00 |

**Sex**

Patients

| **Sex** | | | | | |
| --- | --- | --- | --- | --- | --- |
|  | | Frequency | Percent | Valid Percent | Cumulative Percent |
| Valid | female | 30 | 40.0 | 40.0 | 40.0 |
|  | male | 45 | 60.0 | 60.0 | 100.0 |
|  | Total | 75 | 100.0 | 100.0 |  |

Oliguria

| **Sex** | | | | | |
| --- | --- | --- | --- | --- | --- |
|  | | Frequency | Percent | Valid Percent | Cumulative Percent |
| Valid | female | 6 | 35.3 | 35.3 | 35.3 |
|  | male | 11 | 64.7 | 64.7 | 100.0 |
|  | Total | 17 | 100.0 | 100.0 |  |

Non-oliguria

| **Sex** | | | | | |
| --- | --- | --- | --- | --- | --- |
|  | | Frequency | Percent | Valid Percent | Cumulative Percent |
| Valid | female | 24 | 41.4 | 41.4 | 41.4 |
|  | male | 34 | 58.6 | 58.6 | 100.0 |
|  | Total | 58 | 100.0 | 100.0 |  |

P value

| **Oliguria * Sex Crosstabulation** | | | | |
| --- | --- | --- | --- | --- |
| Count | | | | |
|  | | Gender | | Total |
|  |  | female | male |  |
| Oliguria | urine＜500 mL | 6 | 11 | 17 |
|  | urine≧500 mL | 24 | 34 | 58 |
| Total | | 30 | 45 | 75 |

| **Chi-Square Tests** | | | | | |
| --- | --- | --- | --- | --- | --- |
|  | Value | df | Asymp. Sig. (2-sided) | Exact Sig. (2-sided) | Exact Sig. (1-sided) |
| Pearson Chi-Square | .203^a^ | 1 | .652 |  |  |
| Continuity Correction^b^ | .029 | 1 | .866 |  |  |
| Likelihood Ratio | .205 | 1 | .651 |  |  |
| Fisher's Exact Test |  |  |  | .781 | .437 |
| Linear-by-Linear Association | .200 | 1 | .655 |  |  |
| N of Valid Cases | 75 |  |  |  |  |
| a. 0 cells (.0%) have expected count less than 5. The minimum expected count is 6.80. | | | | | |
| b. Computed only for a 2x2 table | | | | | |

**Body height**

| **Tests of Normality** | | | | | | |
| --- | --- | --- | --- | --- | --- | --- |
|  | Kolmogorov-Smirnov^a^ | | | Shapiro-Wilk | | |
|  | Statistic | df | Sig. | Statistic | df | Sig. |
| Body height(cm) | .071 | 75 | .200^*^ | .983 | 75 | .421 |
| *. This is a lower bound of the true significance. | | | | | | |
| a. Lilliefors Significance Correction | | | | | | |

Patients

| **Descriptive Statistics** | | | | | |
| --- | --- | --- | --- | --- | --- |
|  | N | Minimum | Maximum | Mean | Std. Deviation |
| Body height (cm) | 75 | 144 | 177 | 160.05 | 7.779 |
| Valid N (listwise) | 75 |  |  |  |  |

Oliguria

| **Descriptive Statistics** | | | | | |
| --- | --- | --- | --- | --- | --- |
|  | N | Minimum | Maximum | Mean | Std. Deviation |
| Body height (cm) | 17 | 147 | 174 | 160.53 | 8.625 |
| Valid N (listwise) | 17 |  |  |  |  |

Non-oliguria

| **Descriptive Statistics** | | | | | |
| --- | --- | --- | --- | --- | --- |
|  | N | Minimum | Maximum | Mean | Std. Deviation |
| Body height (cm) | 58 | 144 | 177 | 159.91 | 7.589 |
| Valid N (listwise) | 58 |  |  |  |  |

P value

| **Group Statistics** | | | | | | |
| --- | --- | --- | --- | --- | --- | --- |
|  | Oliguria | | N | Mean | Std. Deviation | Std. Error Mean |
| Body height (cm) |  | urine＜500 mL | 17 | 160.53 | 8.625 | 2.092 |
|  |  | urine≧500 mL | 58 | 159.91 | 7.589 | .996 |

| **Independent Samples Test** | | | | | | | | | | |
| --- | --- | --- | --- | --- | --- | --- | --- | --- | --- | --- |
|  | | Levene's Test for Equality of Variances | | t-test for Equality of Means | | | | | | |
|  |  | F | Sig. | t | df | Sig. (2-tailed) | Mean Difference | Std. Error Difference | 95% Confidence Interval of the Difference | |
|  |  |  |  |  |  |  |  |  | Lower | Upper |
| Body height (cm) | Equal variances assumed | .195 | .660 | .285 | 73 | .776 | .616 | 2.159 | -3.687 | 4.918 |
|  | Equal variances not assumed |  |  | .266 | 23.742 | .793 | .616 | 2.317 | -4.169 | 5.401 |

**Body weight**

| **Tests of Normality** | | | | | | |
| --- | --- | --- | --- | --- | --- | --- |
|  | Kolmogorov-Smirnov^a^ | | | Shapiro-Wilk | | |
|  | Statistic | df | Sig. | Statistic | df | Sig. |
| Body weight (kg) | .071 | 75 | .200^*^ | .986 | 75 | .595 |
| *. This is a lower bound of the true significance. | | | | | | |
| a. Lilliefors Significance Correction | | | | | | |

Patients

| **Descriptive Statistics** | | | | | |
| --- | --- | --- | --- | --- | --- |
|  | N | Minimum | Maximum | Mean | Std. Deviation |
| Body weight (kg) | 75 | 34 | 87 | 58.03 | 12.255 |
| Valid N (listwise) | 75 |  |  |  |  |

Oliguria

| **Descriptive Statistics** | | | | | |
| --- | --- | --- | --- | --- | --- |
|  | N | Minimum | Maximum | Mean | Std. Deviation |
| Body weight (kg) | 17 | 34 | 82 | 61.18 | 14.698 |
| Valid N (listwise) | 17 |  |  |  |  |

Non-oliguria

| **Descriptive Statistics** | | | | | |
| --- | --- | --- | --- | --- | --- |
|  | N | Minimum | Maximum | Mean | Std. Deviation |
| Body weight (kg) | 58 | 36 | 87 | 57.10 | 11.424 |
| Valid N (listwise) | 58 |  |  |  |  |

P value

| **Group Statistics** | | | | | | |
| --- | --- | --- | --- | --- | --- | --- |
|  | Oliguria | | N | Mean | Std. Deviation | Std. Error Mean |
| Body weight (kg) |  | urine＜500 mL | 17 | 61.18 | 14.698 | 3.565 |
|  |  | urine≧500 mL | 58 | 57.10 | 11.424 | 1.500 |

| **Independent Samples Test** | | | | | | | | | | |
| --- | --- | --- | --- | --- | --- | --- | --- | --- | --- | --- |
|  | | Levene's Test for Equality of Variances | | t-test for Equality of Means | | | | | | |
|  |  | F | Sig. | t | df | Sig. (2-tailed) | Mean Difference | Std. Error Difference | 95% Confidence Interval of the Difference | |
|  |  |  |  |  |  |  |  |  | Lower | Upper |
| Body weight (kg) | Equal variances assumed | 2.713 | .104 | 1.209 | 73 | .231 | 4.073 | 3.369 | -2.642 | 10.788 |
|  | Equal variances not assumed |  |  | 1.053 | 21.975 | .304 | 4.073 | 3.868 | -3.948 | 12.094 |

**Body mass index(BMI)**

| **Tests of Normality** | | | | | | |
| --- | --- | --- | --- | --- | --- | --- |
|  | Kolmogorov-Smirnov^a^ | | | Shapiro-Wilk | | |
|  | Statistic | df | Sig. | Statistic | df | Sig. |
| Body mass index | .069 | 75 | .200^*^ | .975 | 75 | .153 |
| *. This is a lower bound of the true significance. | | | | | | |
| a. Lilliefors Significance Correction | | | | | | |

Patients

| **Descriptive Statistics** | | | | | |
| --- | --- | --- | --- | --- | --- |
|  | N | Minimum | Maximum | Mean | Std. Deviation |
| Body mass index | 75 | 13.4 | 34.2 | 22.646 | 4.5638 |
| Valid N (listwise) | 75 |  |  |  |  |

Oliguria

| **Descriptive Statistics** | | | | | |
| --- | --- | --- | --- | --- | --- |
|  | N | Minimum | Maximum | Mean | Std. Deviation |
| Body mass index | 17 | 13.4 | 34.2 | 23.838 | 6.0112 |
| Valid N (listwise) | 17 |  |  |  |  |

Non-oliguria

| **Descriptive Statistics** | | | | | |
| --- | --- | --- | --- | --- | --- |
|  | N | Minimum | Maximum | Mean | Std. Deviation |
| Body mass index | 58 | 14.4 | 33.2 | 22.297 | 4.0434 |
| Valid N (listwise) | 58 |  |  |  |  |

P value

| **Group Statistics** | | | | | | |
| --- | --- | --- | --- | --- | --- | --- |
|  | Oliguria | | N | Mean | Std. Deviation | Std. Error Mean |
| Body mass index |  | urine＜500 mL | 17 | 23.838 | 6.0112 | 1.4579 |
|  |  | urine≧500 mL | 58 | 22.297 | 4.0434 | .5309 |

| **Independent Samples Test** | | | | | | | | | | |
| --- | --- | --- | --- | --- | --- | --- | --- | --- | --- | --- |
|  | | Levene's Test for Equality of Variances | | t-test for Equality of Means | | | | | | |
|  |  | F | Sig. | t | df | Sig. (2-tailed) | Mean Difference | Std. Error Difference | 95% Confidence Interval of the Difference | |
|  |  |  |  |  |  |  |  |  | Lower | Upper |
| Body mass index | Equal variances assumed | 4.637 | .035 | 1.229 | 73 | .223 | 1.5416 | 1.2544 | -.9583 | 4.0416 |
|  | Equal variances not assumed |  |  | .994 | 20.424 | .332 | 1.5416 | 1.5516 | -1.6906 | 4.7739 |

**Septic shock with using vasoactive agents**

Patients

| **Septic shock with using vasoactive agents** | | | | | |
| --- | --- | --- | --- | --- | --- |
|  | | Frequency | Percent | Valid Percent | Cumulative Percent |
| Valid | 0 | 31 | 41.3 | 41.3 | 41.3 |
|  | 1 | 44 | 58.7 | 58.7 | 100.0 |
|  | Total | 75 | 100.0 | 100.0 |  |

Oliguria

| **Septic shock with using vasoactive agents** | | | | | |
| --- | --- | --- | --- | --- | --- |
|  | | Frequency | Percent | Valid Percent | Cumulative Percent |
| Valid | 0 | 3 | 17.6 | 17.6 | 17.6 |
|  | 1 | 14 | 82.4 | 82.4 | 100.0 |
|  | Total | 17 | 100.0 | 100.0 |  |

Non-oliguria

| **Septic shock with using vasoactive agents** | | | | | |
| --- | --- | --- | --- | --- | --- |
|  | | Frequency | Percent | Valid Percent | Cumulative Percent |
| Valid | 0 | 28 | 48.3 | 48.3 | 48.3 |
|  | 1 | 30 | 51.7 | 51.7 | 100.0 |
|  | Total | 58 | 100.0 | 100.0 |  |

P value

| **Oliguria * Septic shock with using vasoactive agents Crosstabulation** | | | | |
| --- | --- | --- | --- | --- |
| Count | | | | |
|  | | Septic shock with using vasoactive agents | | Total |
|  |  | 0 | 1 |  |
| Oliguria | urine＜500 mL | 3 | 14 | 17 |
|  | urine≧500 mL | 28 | 30 | 58 |
| Total | | 31 | 44 | 75 |

| **Chi-Square Tests** | | | | | |
| --- | --- | --- | --- | --- | --- |
|  | Value | df | Asymp. Sig. (2-sided) | Exact Sig. (2-sided) | Exact Sig. (1-sided) |
| Pearson Chi-Square | 5.086^a^ | 1 | .024 |  |  |
| Continuity Correction^b^ | 3.901 | 1 | .048 |  |  |
| Likelihood Ratio | 5.527 | 1 | .019 |  |  |
| Fisher's Exact Test |  |  |  | .028 | .021 |
| Linear-by-Linear Association | 5.018 | 1 | .025 |  |  |
| N of Valid Cases | 75 |  |  |  |  |
| a. 0 cells (.0%) have expected count less than 5. The minimum expected count is 7.03. | | | | | |
| b. Computed only for a 2x2 table | | | | | |

**Glasgow coma scale**

| **Tests of Normality** | | | | | | |
| --- | --- | --- | --- | --- | --- | --- |
|  | Kolmogorov-Smirnov^a^ | | | Shapiro-Wilk | | |
|  | Statistic | df | Sig. | Statistic | df | Sig. |
| Glasgow coma scale | .174 | 75 | .000 | .873 | 75 | .000 |
| a. Lilliefors Significance Correction | | | | | | |

Patients

| **Descriptives** | | | | |
| --- | --- | --- | --- | --- |
|  | | | Statistic | Std. Error |
| Glasgow coma scale | Mean | | 9.37 | .491 |
|  | 95% Confidence Interval for Mean | Lower Bound | 8.39 |  |
|  |  | Upper Bound | 10.35 |  |
|  | 5% Trimmed Mean | | 9.41 |  |
|  | Median | | 8.00 |  |
|  | Variance | | 18.102 |  |
|  | Std. Deviation | | 4.255 |  |
|  | Minimum | | 3 |  |
|  | Maximum | | 15 |  |
|  | Range | | 12 |  |
|  | Interquartile Range | | 9 |  |
|  | Skewness | | .130 | .277 |
|  | Kurtosis | | -1.335 | .548 |

| **Statistics** | | |
| --- | --- | --- |
| Glasgow coma scale | | |
| N | Valid | 75 |
|  | Missing | 0 |
| Percentiles | 25 | 6.00 |
|  | 50 | 8.00 |
|  | 75 | 15.00 |

Oliguria

| **Descriptives** | | | | |
| --- | --- | --- | --- | --- |
|  | | | Statistic | Std. Error |
| Glasgow coma scale | Mean | | 6.53 | 1.061 |
|  | 95% Confidence Interval for Mean | Lower Bound | 4.28 |  |
|  |  | Upper Bound | 8.78 |  |
|  | 5% Trimmed Mean | | 6.25 |  |
|  | Median | | 6.00 |  |
|  | Variance | | 19.140 |  |
|  | Std. Deviation | | 4.375 |  |
|  | Minimum | | 3 |  |
|  | Maximum | | 15 |  |
|  | Range | | 12 |  |
|  | Interquartile Range | | 7 |  |
|  | Skewness | | 1.010 | .550 |
|  | Kurtosis | | -.342 | 1.063 |

| **Statistics** | | |
| --- | --- | --- |
| Glasgow coma scale | | |
| N | Valid | 17 |
|  | Missing | 0 |
| Percentiles | 25 | 3.00 |
|  | 50 | 6.00 |
|  | 75 | 10.00 |

Non-oliguria

| **Descriptives** | | | | |
| --- | --- | --- | --- | --- |
|  | | | Statistic | Std. Error |
| Glasgow coma scale | Mean | | 10.21 | .509 |
|  | 95% Confidence Interval for Mean | Lower Bound | 9.19 |  |
|  |  | Upper Bound | 11.23 |  |
|  | 5% Trimmed Mean | | 10.29 |  |
|  | Median | | 9.00 |  |
|  | Variance | | 15.009 |  |
|  | Std. Deviation | | 3.874 |  |
|  | Minimum | | 3 |  |
|  | Maximum | | 15 |  |
|  | Range | | 12 |  |
|  | Interquartile Range | | 8 |  |
|  | Skewness | | .111 | .314 |
|  | Kurtosis | | -1.476 | .618 |

| **Statistics** | | |
| --- | --- | --- |
| Glasgow coma scale | | |
| N | Valid | 58 |
|  | Missing | 0 |
| Percentiles | 25 | 7.00 |
|  | 50 | 9.00 |
|  | 75 | 15.00 |

**Acute Physiology and Chronic Health Evaluation Score II**

| **Tests of Normality** | | | | | | |
| --- | --- | --- | --- | --- | --- | --- |
|  | Kolmogorov-Smirnov^a^ | | | Shapiro-Wilk | | |
|  | Statistic | df | Sig. | Statistic | df | Sig. |
| Acute Physiology and Chronic Health Evaluation Score II | .067 | 75 | .200^*^ | .985 | 75 | .529 |
| *. This is a lower bound of the true significance. | | | | | | |
| a. Lilliefors Significance Correction | | | | | | |

Patients

| **Descriptive Statistics** | | | | | |
| --- | --- | --- | --- | --- | --- |
|  | N | Minimum | Maximum | Mean | Std. Deviation |
| Acute Physiology and Chronic Health Evaluation Score II | 75 | 6 | 37 | 18.89 | 6.665 |
| Valid N (listwise) | 75 |  |  |  |  |

Oliguria

| **Descriptive Statistics** | | | | | |
| --- | --- | --- | --- | --- | --- |
|  | N | Minimum | Maximum | Mean | Std. Deviation |
| Acute Physiology and Chronic Health Evaluation Score II | 17 | 13 | 37 | 25.76 | 6.088 |
| Valid N (listwise) | 17 |  |  |  |  |

Non-oliguria

| **Descriptive Statistics** | | | | | |
| --- | --- | --- | --- | --- | --- |
|  | N | Minimum | Maximum | Mean | Std. Deviation |
| Acute Physiology and Chronic Health Evaluation Score II | 58 | 6 | 29 | 16.88 | 5.390 |
| Valid N (listwise) | 58 |  |  |  |  |

P value

| **Group Statistics** | | | | | | |
| --- | --- | --- | --- | --- | --- | --- |
|  | Oliguria | | N | Mean | Std. Deviation | Std. Error Mean |
| Acute Physiology and Chronic Health Evaluation Score II |  | urine＜500 mL | 17 | 25.76 | 6.088 | 1.477 |
|  |  | urine≧500 mL | 58 | 16.88 | 5.390 | .708 |

| **Independent Samples Test** | | | | | | | | | | |
| --- | --- | --- | --- | --- | --- | --- | --- | --- | --- | --- |
|  | | Levene's Test for Equality of Variances | | t-test for Equality of Means | | | | | | |
|  |  | F | Sig. | t | df | Sig. (2-tailed) | Mean Difference | Std. Error Difference | 95% Confidence Interval of the Difference | |
|  |  |  |  |  |  |  |  |  | Lower | Upper |
| Acute Physiology and Chronic Health Evaluation Score II | Equal variances assumed | .022 | .884 | 5.804 | 73 | .000 | 8.885 | 1.531 | 5.834 | 11.936 |
|  | Equal variances not assumed |  |  | 5.426 | 23.844 | .000 | 8.885 | 1.637 | 5.505 | 12.266 |

**Mean arterial pressure**

| **Tests of Normality** | | | | | | |
| --- | --- | --- | --- | --- | --- | --- |
|  | Kolmogorov-Smirnov^a^ | | | Shapiro-Wilk | | |
|  | Statistic | df | Sig. | Statistic | df | Sig. |
| Mean arterial pressure (mmHg) | .105 | 75 | .039 | .956 | 75 | .011 |
| a. Lilliefors Significance Correction | | | | | | |

Patients

| **Descriptives** | | | | |
| --- | --- | --- | --- | --- |
|  | | | Statistic | Std. Error |
| Mean arterial pressure (mmHg) | Mean | | 87.933 | 1.5911 |
|  | 95% Confidence Interval for Mean | Lower Bound | 84.763 |  |
|  |  | Upper Bound | 91.104 |  |
|  | 5% Trimmed Mean | | 87.200 |  |
|  | Median | | 87.000 |  |
|  | Variance | | 189.860 |  |
|  | Std. Deviation | | 13.7790 |  |
|  | Minimum | | 62.0 |  |
|  | Maximum | | 130.0 |  |
|  | Range | | 68.0 |  |
|  | Interquartile Range | | 16.5 |  |
|  | Skewness | | .742 | .277 |
|  | Kurtosis | | 1.095 | .548 |

| **Statistics** | | |
| --- | --- | --- |
| Mean arterial pressure (mmHg) | | |
| N | Valid | 75 |
|  | Missing | 0 |
| Percentiles | 25 | 78.000 |
|  | 50 | 87.000 |
|  | 75 | 94.500 |

Oliguria

| **Descriptives** | | | | |
| --- | --- | --- | --- | --- |
|  | | | Statistic | Std. Error |
| Mean arterial pressure (mmHg) | Mean | | 88.441 | 3.0869 |
|  | 95% Confidence Interval for Mean | Lower Bound | 81.897 |  |
|  |  | Upper Bound | 94.985 |  |
|  | 5% Trimmed Mean | | 88.212 |  |
|  | Median | | 87.000 |  |
|  | Variance | | 161.996 |  |
|  | Std. Deviation | | 12.7278 |  |
|  | Minimum | | 68.5 |  |
|  | Maximum | | 112.5 |  |
|  | Range | | 44.0 |  |
|  | Interquartile Range | | 20.0 |  |
|  | Skewness | | .402 | .550 |
|  | Kurtosis | | -.382 | 1.063 |

| **Statistics** | | |
| --- | --- | --- |
| Mean arterial pressure (mmHg) | | |
| N | Valid | 17 |
|  | Missing | 0 |
| Percentiles | 25 | 77.750 |
|  | 50 | 87.000 |
|  | 75 | 97.750 |

Non-oliguria

| **Descriptives** | | | | |
| --- | --- | --- | --- | --- |
|  | | | Statistic | Std. Error |
| Mean arterial pressure (mmHg) | Mean | | 87.784 | 1.8612 |
|  | 95% Confidence Interval for Mean | Lower Bound | 84.058 |  |
|  |  | Upper Bound | 91.511 |  |
|  | 5% Trimmed Mean | | 86.904 |  |
|  | Median | | 87.250 |  |
|  | Variance | | 200.913 |  |
|  | Std. Deviation | | 14.1744 |  |
|  | Minimum | | 62.0 |  |
|  | Maximum | | 130.0 |  |
|  | Range | | 68.0 |  |
|  | Interquartile Range | | 16.8 |  |
|  | Skewness | | .824 | .314 |
|  | Kurtosis | | 1.415 | .618 |

| **Statistics** | | |
| --- | --- | --- |
| Mean arterial pressure (mmHg) | | |
| N | Valid | 58 |
|  | Missing | 0 |
| Percentiles | 25 | 77.750 |
|  | 50 | 87.250 |
|  | 75 | 94.500 |

**Systolic arterial pressure**

| **Tests of Normality** | | | | | | |
| --- | --- | --- | --- | --- | --- | --- |
|  | Kolmogorov-Smirnov^a^ | | | Shapiro-Wilk | | |
|  | Statistic | df | Sig. | Statistic | df | Sig. |
| Systolic arterial pressure (mmHg) | .091 | 75 | .195 | .968 | 75 | .052 |
| a. Lilliefors Significance Correction | | | | | | |

Patients

| **Descriptive Statistics** | | | | | |
| --- | --- | --- | --- | --- | --- |
|  | N | Minimum | Maximum | Mean | Std. Deviation |
| Systolic arterial pressure (mmHg) | 75 | 79.0 | 180.0 | 114.627 | 19.5371 |
| Valid N (listwise) | 75 |  |  |  |  |

Oliguria

| **Descriptive Statistics** | | | | | |
| --- | --- | --- | --- | --- | --- |
|  | N | Minimum | Maximum | Mean | Std. Deviation |
| Systolic arterial pressure (mmHg) | 17 | 90.0 | 156.0 | 116.412 | 18.8151 |
| Valid N (listwise) | 17 |  |  |  |  |

Non-oliguria

| **Descriptive Statistics** | | | | | |
| --- | --- | --- | --- | --- | --- |
|  | N | Minimum | Maximum | Mean | Std. Deviation |
| Systolic arterial pressure (mmHg) | 58 | 79.0 | 180.0 | 114.103 | 19.8730 |
| Valid N (listwise) | 58 |  |  |  |  |

P value

| **Group Statistics** | | | | | | |
| --- | --- | --- | --- | --- | --- | --- |
|  | Oliguria | | N | Mean | Std. Deviation | Std. Error Mean |
| Systolic arterial pressure (mmHg) | 1 | urine＜500 mL | 17 | 116.412 | 18.8151 | 4.5633 |
|  |  | urine≧500 mL | 58 | 114.103 | 19.8730 | 2.6095 |

| **Independent Samples Test** | | | | | | | | | | |
| --- | --- | --- | --- | --- | --- | --- | --- | --- | --- | --- |
|  | | Levene's Test for Equality of Variances | | t-test for Equality of Means | | | | | | |
|  |  | F | Sig. | t | df | Sig. (2-tailed) | Mean Difference | Std. Error Difference | 95% Confidence Interval of the Difference | |
|  |  |  |  |  |  |  |  |  | Lower | Upper |
| Systolic arterial pressure (mmHg) | Equal variances assumed | .415 | .522 | .426 | 73 | .671 | 2.3083 | 5.4183 | -8.4904 | 13.1071 |
|  | Equal variances not assumed |  |  | .439 | 27.354 | .664 | 2.3083 | 5.2567 | -8.4711 | 13.0877 |

**Diastolic arterial pressure**

| **Tests of Normality** | | | | | | |
| --- | --- | --- | --- | --- | --- | --- |
|  | Kolmogorov-Smirnov^a^ | | | Shapiro-Wilk | | |
|  | Statistic | df | Sig. | Statistic | df | Sig. |
| Diastolic arterial pressure (mmHg) | .127 | 75 | .004 | .961 | 75 | .021 |
| a. Lilliefors Significance Correction | | | | | | |

Patients

| **Descriptives** | | | | |
| --- | --- | --- | --- | --- |
|  | | | Statistic | Std. Error |
| Diastolic arterial pressure (mmHg) | Mean | | 61.240 | 1.4157 |
|  | 95% Confidence Interval for Mean | Lower Bound | 58.419 |  |
|  |  | Upper Bound | 64.061 |  |
|  | 5% Trimmed Mean | | 60.674 |  |
|  | Median | | 59.000 |  |
|  | Variance | | 150.320 |  |
|  | Std. Deviation | | 12.2605 |  |
|  | Minimum | | 39.0 |  |
|  | Maximum | | 99.0 |  |
|  | Range | | 60.0 |  |
|  | Interquartile Range | | 17.0 |  |
|  | Skewness | | .722 | .277 |
|  | Kurtosis | | .708 | .548 |

| **Statistics** | | |
| --- | --- | --- |
| Diastolic arterial pressure (mmHg) | | |
| N | Valid | 75 |
|  | Missing | 0 |
| Percentiles | 25 | 53.000 |
|  | 50 | 59.000 |
|  | 75 | 70.000 |

Oliguria

| **Descriptives** | | | | |
| --- | --- | --- | --- | --- |
|  | | | Statistic | Std. Error |
| Diastolic arterial pressure (mmHg) | Mean | | 60.471 | 2.7880 |
|  | 95% Confidence Interval for Mean | Lower Bound | 54.560 |  |
|  |  | Upper Bound | 66.381 |  |
|  | 5% Trimmed Mean | | 60.634 |  |
|  | Median | | 62.000 |  |
|  | Variance | | 132.140 |  |
|  | Std. Deviation | | 11.4952 |  |
|  | Minimum | | 41.0 |  |
|  | Maximum | | 77.0 |  |
|  | Range | | 36.0 |  |
|  | Interquartile Range | | 19.5 |  |
|  | Skewness | | -.263 | .550 |
|  | Kurtosis | | -.831 | 1.063 |

| **Statistics** | | |
| --- | --- | --- |
| Diastolic arterial pressure (mmHg) | | |
| N | Valid | 17 |
|  | Missing | 0 |
| Percentiles | 25 | 51.000 |
|  | 50 | 62.000 |
|  | 75 | 70.500 |

Non-oliguria

| **Descriptives** | | | | |
| --- | --- | --- | --- | --- |
|  | | | Statistic | Std. Error |
| Diastolic arterial pressure (mmHg) | Mean | | 61.466 | 1.6496 |
|  | 95% Confidence Interval for Mean | Lower Bound | 58.162 |  |
|  |  | Upper Bound | 64.769 |  |
|  | 5% Trimmed Mean | | 60.795 |  |
|  | Median | | 58.000 |  |
|  | Variance | | 157.832 |  |
|  | Std. Deviation | | 12.5631 |  |
|  | Minimum | | 39.0 |  |
|  | Maximum | | 99.0 |  |
|  | Range | | 60.0 |  |
|  | Interquartile Range | | 16.3 |  |
|  | Skewness | | .919 | .314 |
|  | Kurtosis | | .944 | .618 |

| **Statistics** | | |
| --- | --- | --- |
| Diastolic arterial pressure (mmHg) | | |
| N | Valid | 58 |
|  | Missing | 0 |
| Percentiles | 25 | 53.750 |
|  | 50 | 58.000 |
|  | 75 | 70.000 |

**White blood cells**

| **Tests of Normality** | | | | | | |
| --- | --- | --- | --- | --- | --- | --- |
|  | Kolmogorov-Smirnov^a^ | | | Shapiro-Wilk | | |
|  | Statistic | df | Sig. | Statistic | df | Sig. |
| White blood cells (1000/uL) | .089 | 75 | .200^*^ | .929 | 75 | .000 |
| *. This is a lower bound of the true significance. | | | | | | |
| a. Lilliefors Significance Correction | | | | | | |

Patients

| **Descriptive Statistics** | | | | | |
| --- | --- | --- | --- | --- | --- |
|  | N | Minimum | Maximum | Mean | Std. Deviation |
| White blood cells (1000/uL) | 75 | .5 | 47.6 | 13.537 | 8.6535 |
| Valid N (listwise) | 75 |  |  |  |  |

Oliguria

| **Descriptive Statistics** | | | | | |
| --- | --- | --- | --- | --- | --- |
|  | N | Minimum | Maximum | Mean | Std. Deviation |
| White blood cells (1000/uL) | 17 | 1.5 | 37.3 | 15.406 | 8.9982 |
| Valid N (listwise) | 17 |  |  |  |  |

Non-oliguria

| **Descriptive Statistics** | | | | | |
| --- | --- | --- | --- | --- | --- |
|  | N | Minimum | Maximum | Mean | Std. Deviation |
| White blood cells (1000/uL) | 58 | .5 | 47.6 | 12.990 | 8.5523 |
| Valid N (listwise) | 58 |  |  |  |  |

P value

| **Group Statistics** | | | | | | |
| --- | --- | --- | --- | --- | --- | --- |
|  | Oliguria | | N | Mean | Std. Deviation | Std. Error Mean |
| White blood cells (1000/uL) |  | urine＜500 mL | 17 | 15.406 | 8.9982 | 2.1824 |
|  |  | urine≧500 mL | 58 | 12.990 | 8.5523 | 1.1230 |

| **Independent Samples Test** | | | | | | | | | | |
| --- | --- | --- | --- | --- | --- | --- | --- | --- | --- | --- |
|  | | Levene's Test for Equality of Variances | | t-test for Equality of Means | | | | | | |
|  |  | F | Sig. | t | df | Sig. (2-tailed) | Mean Difference | Std. Error Difference | 95% Confidence Interval of the Difference | |
|  |  |  |  |  |  |  |  |  | Lower | Upper |
| White blood cells (1000/uL) | Equal variances assumed | .001 | .979 | 1.013 | 73 | .315 | 2.4162 | 2.3862 | -2.3395 | 7.1719 |
|  | Equal variances not assumed |  |  | .984 | 25.100 | .334 | 2.4162 | 2.4544 | -2.6376 | 7.4701 |

**Hemoglobin**

| **Tests of Normality** | | | | | | |
| --- | --- | --- | --- | --- | --- | --- |
|  | Kolmogorov-Smirnov^a^ | | | Shapiro-Wilk | | |
|  | Statistic | df | Sig. | Statistic | df | Sig. |
| Hemoglobin (g/dL) | .084 | 75 | .200^*^ | .971 | 75 | .080 |
| *. This is a lower bound of the true significance. | | | | | | |
| a. Lilliefors Significance Correction | | | | | | |

Patients

| **Descriptive Statistics** | | | | | |
| --- | --- | --- | --- | --- | --- |
|  | N | Minimum | Maximum | Mean | Std. Deviation |
| Hemoglobin (g/dL) | 75 | 5.8 | 16.1 | 11.155 | 2.6404 |
| Valid N (listwise) | 75 |  |  |  |  |

Oliguria

| **Descriptive Statistics** | | | | | |
| --- | --- | --- | --- | --- | --- |
|  | N | Minimum | Maximum | Mean | Std. Deviation |
| Hemoglobin (g/dL) | 17 | 6.4 | 15.0 | 10.365 | 2.5497 |
| Valid N (listwise) | 17 |  |  |  |  |

Non-oliguria

| **Descriptive Statistics** | | | | | |
| --- | --- | --- | --- | --- | --- |
|  | N | Minimum | Maximum | Mean | Std. Deviation |
| Hemoglobin (g/dL) | 58 | 5.8 | 16.1 | 11.386 | 2.6431 |
| Valid N (listwise) | 58 |  |  |  |  |

P value

| **Group Statistics** | | | | | | |
| --- | --- | --- | --- | --- | --- | --- |
|  | Oliguria | | N | Mean | Std. Deviation | Std. Error Mean |
| Hemoglobin (g/dL) |  | urine＜500 mL | 17 | 10.365 | 2.5497 | .6184 |
|  |  | urine≧500 mL | 58 | 11.386 | 2.6431 | .3471 |

| **Independent Samples Test** | | | | | | | | | | |
| --- | --- | --- | --- | --- | --- | --- | --- | --- | --- | --- |
|  | | Levene's Test for Equality of Variances | | t-test for Equality of Means | | | | | | |
|  |  | F | Sig. | t | df | Sig. (2-tailed) | Mean Difference | Std. Error Difference | 95% Confidence Interval of the Difference | |
|  |  |  |  |  |  |  |  |  | Lower | Upper |
| Hemoglobin (g/dL) | Equal variances assumed | .153 | .697 | -1.412 | 73 | .162 | -1.0215 | .7234 | -2.4632 | .4202 |
|  | Equal variances not assumed |  |  | -1.440 | 26.916 | .161 | -1.0215 | .7091 | -2.4767 | .4337 |

**Creatinine**

| **Tests of Normality** | | | | | | |
| --- | --- | --- | --- | --- | --- | --- |
|  | Kolmogorov-Smirnov^a^ | | | Shapiro-Wilk | | |
|  | Statistic | df | Sig. | Statistic | df | Sig. |
| Creatinine (mg/dL) | .209 | 75 | .000 | .613 | 75 | .000 |
| a. Lilliefors Significance Correction | | | | | | |

Patients

| **Descriptives** | | | | |
| --- | --- | --- | --- | --- |
|  | | | Statistic | Std. Error |
| Creatinine (mg/dL) | Mean | | 2.1115 | .23515 |
|  | 95% Confidence Interval for Mean | Lower Bound | 1.6429 |  |
|  |  | Upper Bound | 2.5800 |  |
|  | 5% Trimmed Mean | | 1.8450 |  |
|  | Median | | 1.6700 |  |
|  | Variance | | 4.147 |  |
|  | Std. Deviation | | 2.03645 |  |
|  | Minimum | | .37 |  |
|  | Maximum | | 15.66 |  |
|  | Range | | 15.29 |  |
|  | Interquartile Range | | 1.47 |  |
|  | Skewness | | 4.357 | .277 |
|  | Kurtosis | | 26.349 | .548 |

| **Statistics** | | |
| --- | --- | --- |
| Creatinine (mg/dL) | | |
| N | Valid | 75 |
|  | Missing | 0 |
| Percentiles | 25 | 1.0200 |
|  | 50 | 1.6700 |
|  | 75 | 2.4900 |

Oliguria

| **Descriptives** | | | | |
| --- | --- | --- | --- | --- |
|  | | | Statistic | Std. Error |
| Creatinine (mg/dL) | Mean | | 3.3041 | .84781 |
|  | 95% Confidence Interval for Mean | Lower Bound | 1.5068 |  |
|  |  | Upper Bound | 5.1014 |  |
|  | 5% Trimmed Mean | | 2.7796 |  |
|  | Median | | 2.4100 |  |
|  | Variance | | 12.219 |  |
|  | Std. Deviation | | 3.49563 |  |
|  | Minimum | | .39 |  |
|  | Maximum | | 15.66 |  |
|  | Range | | 15.27 |  |
|  | Interquartile Range | | 1.82 |  |
|  | Skewness | | 3.077 | .550 |
|  | Kurtosis | | 10.724 | 1.063 |

| **Statistics** | | |
| --- | --- | --- |
| Creatinine (mg/dL) | | |
| N | Valid | 17 |
|  | Missing | 0 |
| Percentiles | 25 | 1.8100 |
|  | 50 | 2.4100 |
|  | 75 | 3.6350 |

Non-oliguria

| **Descriptives** | | | | |
| --- | --- | --- | --- | --- |
|  | | | Statistic | Std. Error |
| Creatinine (mg/dL) | Mean | | 1.7619 | .15566 |
|  | 95% Confidence Interval for Mean | Lower Bound | 1.4502 |  |
|  |  | Upper Bound | 2.0736 |  |
|  | 5% Trimmed Mean | | 1.6423 |  |
|  | Median | | 1.4400 |  |
|  | Variance | | 1.405 |  |
|  | Std. Deviation | | 1.18550 |  |
|  | Minimum | | .37 |  |
|  | Maximum | | 5.72 |  |
|  | Range | | 5.35 |  |
|  | Interquartile Range | | 1.15 |  |
|  | Skewness | | 1.669 | .314 |
|  | Kurtosis | | 2.858 | .618 |

| **Statistics** | | |
| --- | --- | --- |
| Creatinine (mg/dL) | | |
| N | Valid | 58 |
|  | Missing | 0 |
| Percentiles | 25 | .9550 |
|  | 50 | 1.4400 |
|  | 75 | 2.1075 |

**Arterial lactate**

| **Tests of Normality** | | | | | | |
| --- | --- | --- | --- | --- | --- | --- |
|  | Kolmogorov-Smirnov^a^ | | | Shapiro-Wilk | | |
|  | Statistic | df | Sig. | Statistic | df | Sig. |
| Arterial lactate (mg/dL) | .262 | 73 | .000 | .539 | 73 | .000 |
| a. Lilliefors Significance Correction | | | | | | |

Patients

| **Descriptives** | | | | |
| --- | --- | --- | --- | --- |
|  | | | Statistic | Std. Error |
| Arterial lactate (mg/dL) | Mean | | 28.367 | 3.7204 |
|  | 95% Confidence Interval for Mean | Lower Bound | 20.951 |  |
|  |  | Upper Bound | 35.784 |  |
|  | 5% Trimmed Mean | | 23.014 |  |
|  | Median | | 19.400 |  |
|  | Variance | | 1010.442 |  |
|  | Std. Deviation | | 31.7874 |  |
|  | Minimum | | 5.1 |  |
|  | Maximum | | 218.9 |  |
|  | Range | | 213.8 |  |
|  | Interquartile Range | | 16.9 |  |
|  | Skewness | | 4.054 | .281 |
|  | Kurtosis | | 19.688 | .555 |

| **Statistics** | | |
| --- | --- | --- |
| Arterial lactate (mg/dL) | | |
| N | Valid | 73 |
|  | Missing | 2 |
| Percentiles | 25 | 13.600 |
|  | 50 | 19.400 |
|  | 75 | 30.500 |

Oliguria

| **Descriptives** | | | | |
| --- | --- | --- | --- | --- |
|  | | | Statistic | Std. Error |
| Arterial lactate (mg/dL) | Mean | | 55.065 | 13.6501 |
|  | 95% Confidence Interval for Mean | Lower Bound | 26.128 |  |
|  |  | Upper Bound | 84.002 |  |
|  | 5% Trimmed Mean | | 48.494 |  |
|  | Median | | 34.200 |  |
|  | Variance | | 3167.507 |  |
|  | Std. Deviation | | 56.2806 |  |
|  | Minimum | | 9.5 |  |
|  | Maximum | | 218.9 |  |
|  | Range | | 209.4 |  |
|  | Interquartile Range | | 60.2 |  |
|  | Skewness | | 1.902 | .550 |
|  | Kurtosis | | 3.624 | 1.063 |

| **Statistics** | | |
| --- | --- | --- |
| Arterial lactate (mg/dL) | | |
| N | Valid | 17 |
|  | Missing | 0 |
| Percentiles | 25 | 17.500 |
|  | 50 | 34.200 |
|  | 75 | 77.650 |

Non-oliguria

| **Descriptives** | | | | |
| --- | --- | --- | --- | --- |
|  | | | Statistic | Std. Error |
| Arterial lactate (mg/dL) | Mean | | 20.263 | 1.4275 |
|  | 95% Confidence Interval for Mean | Lower Bound | 17.402 |  |
|  |  | Upper Bound | 23.123 |  |
|  | 5% Trimmed Mean | | 19.226 |  |
|  | Median | | 18.250 |  |
|  | Variance | | 114.117 |  |
|  | Std. Deviation | | 10.6825 |  |
|  | Minimum | | 5.1 |  |
|  | Maximum | | 67.9 |  |
|  | Range | | 62.8 |  |
|  | Interquartile Range | | 11.4 |  |
|  | Skewness | | 2.039 | .319 |
|  | Kurtosis | | 6.349 | .628 |

| **Statistics** | | |
| --- | --- | --- |
| Arterial lactate (mg/dL) | | |
| N | Valid | 56 |
|  | Missing | 2 |
| Percentiles | 25 | 13.375 |
|  | 50 | 18.250 |
|  | 75 | 24.775 |

**Vascular endothelial growth factor**

| **Tests of Normality** | | | | | | |
| --- | --- | --- | --- | --- | --- | --- |
|  | Kolmogorov-Smirnov^a^ | | | Shapiro-Wilk | | |
|  | Statistic | df | Sig. | Statistic | df | Sig. |
| VEGF (pg/mL) | .172 | 69 | .000 | .825 | 69 | .000 |
| a. Lilliefors Significance Correction | | | | | | |

Patients

| **Descriptives** | | | | |
| --- | --- | --- | --- | --- |
|  | | | Statistic | Std. Error |
| VEGF (pg/mL) | Mean | | 198.7241 | 23.75761 |
|  | 95% Confidence Interval for Mean | Lower Bound | 151.3166 |  |
|  |  | Upper Bound | 246.1317 |  |
|  | 5% Trimmed Mean | | 178.4468 |  |
|  | Median | | 122.1715 |  |
|  | Variance | | 38945.265 |  |
|  | Std. Deviation | | 197.34555 |  |
|  | Minimum | | 3.17 |  |
|  | Maximum | | 871.87 |  |
|  | Range | | 868.70 |  |
|  | Interquartile Range | | 236.13 |  |
|  | Skewness | | 1.489 | .289 |
|  | Kurtosis | | 1.891 | .570 |

| **Statistics** | | |
| --- | --- | --- |
| VEGF (pg/mL) | | |
| N | Valid | 69 |
|  | Missing | 6 |
| Percentiles | 25 | 47.8930 |
|  | 50 | 122.1715 |
|  | 75 | 284.0197 |

Oliguria

| **Descriptives** | | | | |
| --- | --- | --- | --- | --- |
|  | | | Statistic | Std. Error |
| VEGF (pg/mL) | Mean | | 193.1803 | 54.74871 |
|  | 95% Confidence Interval for Mean | Lower Bound | 75.7560 |  |
|  |  | Upper Bound | 310.6046 |  |
|  | 5% Trimmed Mean | | 175.9742 |  |
|  | Median | | 99.6536 |  |
|  | Variance | | 44961.325 |  |
|  | Std. Deviation | | 212.04086 |  |
|  | Minimum | | 3.17 |  |
|  | Maximum | | 692.90 |  |
|  | Range | | 689.74 |  |
|  | Interquartile Range | | 280.50 |  |
|  | Skewness | | 1.284 | .580 |
|  | Kurtosis | | .752 | 1.121 |

| **Statistics** | | |
| --- | --- | --- |
| VEGF (pg/mL) | | |
| N | Valid | 15 |
|  | Missing | 2 |
| Percentiles | 25 | 37.3399 |
|  | 50 | 99.6536 |
|  | 75 | 317.8435 |

Non-oliguria

| **Descriptives** | | | | |
| --- | --- | --- | --- | --- |
|  | | | Statistic | Std. Error |
| VEGF (pg/mL) | Mean | | 200.2641 | 26.55526 |
|  | 95% Confidence Interval for Mean | Lower Bound | 147.0010 |  |
|  |  | Upper Bound | 253.5272 |  |
|  | 5% Trimmed Mean | | 179.0149 |  |
|  | Median | | 141.8597 |  |
|  | Variance | | 38079.819 |  |
|  | Std. Deviation | | 195.14051 |  |
|  | Minimum | | 13.34 |  |
|  | Maximum | | 871.87 |  |
|  | Range | | 858.53 |  |
|  | Interquartile Range | | 231.83 |  |
|  | Skewness | | 1.604 | .325 |
|  | Kurtosis | | 2.521 | .639 |

| **Statistics** | | |
| --- | --- | --- |
| VEGF (pg/mL) | | |
| N | Valid | 54 |
|  | Missing | 4 |
| Percentiles | 25 | 51.5496 |
|  | 50 | 141.8597 |
|  | 75 | 283.3774 |

**Albumin**

| **Tests of Normality** | | | | | | |
| --- | --- | --- | --- | --- | --- | --- |
|  | Kolmogorov-Smirnov^a^ | | | Shapiro-Wilk | | |
|  | Statistic | df | Sig. | Statistic | df | Sig. |
| Albumin (g/dL) | .083 | 66 | .200^*^ | .990 | 66 | .891 |
| *. This is a lower bound of the true significance. | | | | | | |
| a. Lilliefors Significance Correction | | | | | | |

Patients

| **Descriptive Statistics** | | | | | |
| --- | --- | --- | --- | --- | --- |
|  | N | Minimum | Maximum | Mean | Std. Deviation |
| Albumin (g/dL) | 66 | 1.2 | 4.6 | 2.871 | .6905 |
| Valid N (listwise) | 66 |  |  |  |  |

Oliguria

| **Descriptive Statistics** | | | | | |
| --- | --- | --- | --- | --- | --- |
|  | N | Minimum | Maximum | Mean | Std. Deviation |
| Albumin (g/dL) | 12 | 1.9 | 3.7 | 2.950 | .6882 |
| Valid N (listwise) | 12 |  |  |  |  |

Non-oliguria

| **Descriptive Statistics** | | | | | |
| --- | --- | --- | --- | --- | --- |
|  | N | Minimum | Maximum | Mean | Std. Deviation |
| Albumin (g/dL) | 54 | 1.2 | 4.6 | 2.854 | .6963 |
| Valid N (listwise) | 54 |  |  |  |  |

P value

| **Group Statistics** | | | | | | |
| --- | --- | --- | --- | --- | --- | --- |
|  | Oliguria | | N | Mean | Std. Deviation | Std. Error Mean |
| Albumin (g/dL) |  | urine＜500 mL | 12 | 2.950 | .6882 | .1987 |
|  |  | urine≧500 mL | 54 | 2.854 | .6963 | .0948 |

| **Independent Samples Test** | | | | | | | | | | |
| --- | --- | --- | --- | --- | --- | --- | --- | --- | --- | --- |
|  | | Levene's Test for Equality of Variances | | t-test for Equality of Means | | | | | | |
|  |  | F | Sig. | t | df | Sig. (2-tailed) | Mean Difference | Std. Error Difference | 95% Confidence Interval of the Difference | |
|  |  |  |  |  |  |  |  |  | Lower | Upper |
| Albumin (g/dL) | Equal variances assumed | .281 | .598 | .434 | 64 | .666 | .0963 | .2218 | -.3467 | .5393 |
|  | Equal variances not assumed |  |  | .437 | 16.397 | .667 | .0963 | .2201 | -.3694 | .5620 |

**Partial pressure of oxygen**

| **Tests of Normality** | | | | | | |
| --- | --- | --- | --- | --- | --- | --- |
|  | Kolmogorov-Smirnov^a^ | | | Shapiro-Wilk | | |
|  | Statistic | df | Sig. | Statistic | df | Sig. |
| Partial pressure of oxygen (mmHg) | .217 | 75 | .000 | .693 | 75 | .000 |
| a. Lilliefors Significance Correction | | | | | | |

Patients

| **Descriptives** | | | | |
| --- | --- | --- | --- | --- |
|  | | | Statistic | Std. Error |
| Partial pressure of oxygen (mmHg) | Mean | | 128.201 | 9.0707 |
|  | 95% Confidence Interval for Mean | Lower Bound | 110.128 |  |
|  |  | Upper Bound | 146.275 |  |
|  | 5% Trimmed Mean | | 117.753 |  |
|  | Median | | 104.500 |  |
|  | Variance | | 6170.835 |  |
|  | Std. Deviation | | 78.5547 |  |
|  | Minimum | | 48.7 |  |
|  | Maximum | | 565.5 |  |
|  | Range | | 516.8 |  |
|  | Interquartile Range | | 58.5 |  |
|  | Skewness | | 3.114 | .277 |
|  | Kurtosis | | 13.148 | .548 |

| **Statistics** | | |
| --- | --- | --- |
| Partial pressure of oxygen (mmHg) | | |
| N | Valid | 75 |
|  | Missing | 0 |
| Percentiles | 25 | 82.700 |
|  | 50 | 104.500 |
|  | 75 | 141.200 |

Oliguria

| **Descriptives** | | | | |
| --- | --- | --- | --- | --- |
|  | | | Statistic | Std. Error |
| Partial pressure of oxygen (mmHg) | Mean | | 108.376 | 8.3515 |
|  | 95% Confidence Interval for Mean | Lower Bound | 90.672 |  |
|  |  | Upper Bound | 126.081 |  |
|  | 5% Trimmed Mean | | 107.268 |  |
|  | Median | | 98.700 |  |
|  | Variance | | 1185.711 |  |
|  | Std. Deviation | | 34.4341 |  |
|  | Minimum | | 48.7 |  |
|  | Maximum | | 188.0 |  |
|  | Range | | 139.3 |  |
|  | Interquartile Range | | 56.0 |  |
|  | Skewness | | .549 | .550 |
|  | Kurtosis | | .341 | 1.063 |

| **Statistics** | | |
| --- | --- | --- |
| Partial pressure of oxygen (mmHg) | | |
| N | Valid | 17 |
|  | Missing | 0 |
| Percentiles | 25 | 83.300 |
|  | 50 | 98.700 |
|  | 75 | 139.250 |

Non-oliguria

| **Descriptives** | | | | |
| --- | --- | --- | --- | --- |
|  | | | Statistic | Std. Error |
| Partial pressure of oxygen (mmHg) | Mean | | 134.012 | 11.3918 |
|  | 95% Confidence Interval for Mean | Lower Bound | 111.200 |  |
|  |  | Upper Bound | 156.824 |  |
|  | 5% Trimmed Mean | | 122.657 |  |
|  | Median | | 106.100 |  |
|  | Variance | | 7526.854 |  |
|  | Std. Deviation | | 86.7574 |  |
|  | Minimum | | 56.4 |  |
|  | Maximum | | 565.5 |  |
|  | Range | | 509.1 |  |
|  | Interquartile Range | | 63.7 |  |
|  | Skewness | | 2.843 | .314 |
|  | Kurtosis | | 10.508 | .618 |

| **Statistics** | | |
| --- | --- | --- |
| Partial pressure of oxygen (mmHg) | | |
| N | Valid | 58 |
|  | Missing | 0 |
| Percentiles | 25 | 82.525 |
|  | 50 | 106.100 |
|  | 75 | 146.225 |

**Arterial oxygen saturation**

| **Tests of Normality** | | | | | | |
| --- | --- | --- | --- | --- | --- | --- |
|  | Kolmogorov-Smirnov^a^ | | | Shapiro-Wilk | | |
|  | Statistic | df | Sig. | Statistic | df | Sig. |
| Arterial oxygen saturation (percent) | .218 | 75 | .000 | .602 | 75 | .000 |
| a. Lilliefors Significance Correction | | | | | | |

Patients

| **Descriptives** | | | | |
| --- | --- | --- | --- | --- |
|  | | | Statistic | Std. Error |
| Arterial oxygen saturation (percent) | Mean | | 97.428 | .3809 |
|  | 95% Confidence Interval for Mean | Lower Bound | 96.669 |  |
|  |  | Upper Bound | 98.187 |  |
|  | 5% Trimmed Mean | | 97.840 |  |
|  | Median | | 98.000 |  |
|  | Variance | | 10.880 |  |
|  | Std. Deviation | | 3.2985 |  |
|  | Minimum | | 75.0 |  |
|  | Maximum | | 100.0 |  |
|  | Range | | 25.0 |  |
|  | Interquartile Range | | 2.5 |  |
|  | Skewness | | -4.544 | .277 |
|  | Kurtosis | | 28.708 | .548 |

| **Statistics** | | |
| --- | --- | --- |
| Arterial oxygen saturation (percent) | | |
| N | Valid | 75 |
|  | Missing | 0 |
| Percentiles | 25 | 96.800 |
|  | 50 | 98.000 |
|  | 75 | 99.300 |

Oliguria

| **Descriptives** | | | | |
| --- | --- | --- | --- | --- |
|  | | | Statistic | Std. Error |
| Arterial oxygen saturation (percent) | Mean | | 96.106 | 1.3988 |
|  | 95% Confidence Interval for Mean | Lower Bound | 93.141 |  |
|  |  | Upper Bound | 99.071 |  |
|  | 5% Trimmed Mean | | 97.079 |  |
|  | Median | | 97.900 |  |
|  | Variance | | 33.261 |  |
|  | Std. Deviation | | 5.7672 |  |
|  | Minimum | | 75.0 |  |
|  | Maximum | | 99.7 |  |
|  | Range | | 24.7 |  |
|  | Interquartile Range | | 2.8 |  |
|  | Skewness | | -3.420 | .550 |
|  | Kurtosis | | 12.747 | 1.063 |

| **Statistics** | | |
| --- | --- | --- |
| Arterial oxygen saturation (percent) | | |
| N | Valid | 17 |
|  | Missing | 0 |
| Percentiles | 25 | 96.000 |
|  | 50 | 97.900 |
|  | 75 | 98.850 |

Non-oliguria

| **Descriptives** | | | | |
| --- | --- | --- | --- | --- |
|  | | | Statistic | Std. Error |
| Arterial oxygen saturation (percent) | Mean | | 97.816 | .2663 |
|  | 95% Confidence Interval for Mean | Lower Bound | 97.282 |  |
|  |  | Upper Bound | 98.349 |  |
|  | 5% Trimmed Mean | | 97.996 |  |
|  | Median | | 98.250 |  |
|  | Variance | | 4.114 |  |
|  | Std. Deviation | | 2.0284 |  |
|  | Minimum | | 91.2 |  |
|  | Maximum | | 100.0 |  |
|  | Range | | 8.8 |  |
|  | Interquartile Range | | 2.2 |  |
|  | Skewness | | -1.325 | .314 |
|  | Kurtosis | | 1.409 | .618 |

| **Statistics** | | |
| --- | --- | --- |
| Arterial oxygen saturation (percent) | | |
| N | Valid | 58 |
|  | Missing | 0 |
| Percentiles | 25 | 97.250 |
|  | 50 | 98.250 |
|  | 75 | 99.400 |

**Intake and Output (Data recorded on the day prior to the NIRS measurement)**

**Day 1**

| **Tests of Normality** | | | | | | |
| --- | --- | --- | --- | --- | --- | --- |
|  | Kolmogorov-Smirnov^a^ | | | Shapiro-Wilk | | |
|  | Statistic | df | Sig. | Statistic | df | Sig. |
| Intake and Output (mL/day) | .108 | 68 | .048 | .971 | 68 | .109 |
| a. Lilliefors Significance Correction | | | | | | |

Patients

| **Descriptives** | | | | |
| --- | --- | --- | --- | --- |
|  | | | Statistic | Std. Error |
| Intake and Output (mL/day) | Mean | | 438.18 | 134.729 |
|  | 95% Confidence Interval for Mean | Lower Bound | 169.26 |  |
|  |  | Upper Bound | 707.10 |  |
|  | 5% Trimmed Mean | | 401.20 |  |
|  | Median | | 201.00 |  |
|  | Variance | | 1234332.446 |  |
|  | Std. Deviation | | 1111.005 |  |
|  | Minimum | | -1967 |  |
|  | Maximum | | 4224 |  |
|  | Range | | 6191 |  |
|  | Interquartile Range | | 1489 |  |
|  | Skewness | | .638 | .291 |
|  | Kurtosis | | 1.221 | .574 |

| **Statistics** | | |
| --- | --- | --- |
| Intake and Output (mL/day) | | |
| N | Valid | 68 |
|  | Missing | 7 |
| Percentiles | 25 | -298.75 |
|  | 50 | 201.00 |
|  | 75 | 1190.25 |

Oliguria

| **Descriptives** | | | | |
| --- | --- | --- | --- | --- |
|  | | | Statistic | Std. Error |
| Intake and Output (mL/day) | Mean | | 1060.81 | 371.177 |
|  | 95% Confidence Interval for Mean | Lower Bound | 269.67 |  |
|  |  | Upper Bound | 1851.96 |  |
|  | 5% Trimmed Mean | | 1053.29 |  |
|  | Median | | 1142.00 |  |
|  | Variance | | 2204355.896 |  |
|  | Std. Deviation | | 1484.707 |  |
|  | Minimum | | -1967 |  |
|  | Maximum | | 4224 |  |
|  | Range | | 6191 |  |
|  | Interquartile Range | | 1582 |  |
|  | Skewness | | .062 | .564 |
|  | Kurtosis | | .825 | 1.091 |

| **Statistics** | | |
| --- | --- | --- |
| Intake and Output (mL/day) | | |
| N | Valid | 16 |
|  | Missing | 1 |
| Percentiles | 25 | 147.25 |
|  | 50 | 1142.00 |
|  | 75 | 1729.00 |

Non-oliguria

| **Descriptives** | | | | |
| --- | --- | --- | --- | --- |
|  | | | Statistic | Std. Error |
| Intake and Output (mL/day) | Mean | | 246.60 | 125.130 |
|  | 95% Confidence Interval for Mean | Lower Bound | -4.61 |  |
|  |  | Upper Bound | 497.80 |  |
|  | 5% Trimmed Mean | | 233.76 |  |
|  | Median | | 155.00 |  |
|  | Variance | | 814187.618 |  |
|  | Std. Deviation | | 902.323 |  |
|  | Minimum | | -1856 |  |
|  | Maximum | | 2700 |  |
|  | Range | | 4556 |  |
|  | Interquartile Range | | 1219 |  |
|  | Skewness | | .357 | .330 |
|  | Kurtosis | | .157 | .650 |

| **Statistics** | | |
| --- | --- | --- |
| Intake and Output (mL/day) | | |
| N | Valid | 52 |
|  | Missing | 6 |
| Percentiles | 25 | -384.25 |
|  | 50 | 155.00 |
|  | 75 | 834.50 |

**Day 2**

| **Tests of Normality** | | | | | | |
| --- | --- | --- | --- | --- | --- | --- |
|  | Kolmogorov-Smirnov^a^ | | | Shapiro-Wilk | | |
|  | Statistic | df | Sig. | Statistic | df | Sig. |
| Intake and Output (mL/day) | .115 | 73 | .017 | .947 | 73 | .004 |
| a. Lilliefors Significance Correction | | | | | | |

Patients

| **Descriptives** | | | | |
| --- | --- | --- | --- | --- |
|  | | | Statistic | Std. Error |
| Intake and Output (mL/day) | Mean | | 791.70 | 159.842 |
|  | 95% Confidence Interval for Mean | Lower Bound | 473.06 |  |
|  |  | Upper Bound | 1110.34 |  |
|  | 5% Trimmed Mean | | 727.89 |  |
|  | Median | | 531.00 |  |
|  | Variance | | 1865103.908 |  |
|  | Std. Deviation | | 1365.688 |  |
|  | Minimum | | -2418 |  |
|  | Maximum | | 5325 |  |
|  | Range | | 7743 |  |
|  | Interquartile Range | | 1608 |  |
|  | Skewness | | .859 | .281 |
|  | Kurtosis | | 1.614 | .555 |

| **Statistics** | | |
| --- | --- | --- |
| Intake and Output (mL/day) | | |
| N | Valid | 73 |
|  | Missing | 2 |
| Percentiles | 25 | -132.00 |
|  | 50 | 531.00 |
|  | 75 | 1476.00 |

Oliguria

| **Descriptives** | | | | |
| --- | --- | --- | --- | --- |
|  | | | Statistic | Std. Error |
| Intake and Output (mL/day) | Mean | | 1583.12 | 487.893 |
|  | 95% Confidence Interval for Mean | Lower Bound | 548.83 |  |
|  |  | Upper Bound | 2617.40 |  |
|  | 5% Trimmed Mean | | 1597.52 |  |
|  | Median | | 1872.00 |  |
|  | Variance | | 4046673.985 |  |
|  | Std. Deviation | | 2011.635 |  |
|  | Minimum | | -2418 |  |
|  | Maximum | | 5325 |  |
|  | Range | | 7743 |  |
|  | Interquartile Range | | 2264 |  |
|  | Skewness | | -.233 | .550 |
|  | Kurtosis | | .223 | 1.063 |

| **Statistics** | | |
| --- | --- | --- |
| Intake and Output (mL/day) | | |
| N | Valid | 17 |
|  | Missing | 0 |
| Percentiles | 25 | 135.00 |
|  | 50 | 1872.00 |
|  | 75 | 2398.50 |

Non-oliguria

| **Descriptives** | | | | |
| --- | --- | --- | --- | --- |
|  | | | Statistic | Std. Error |
| Intake and Output (mL/day) | Mean | | 551.45 | 134.431 |
|  | 95% Confidence Interval for Mean | Lower Bound | 282.04 |  |
|  |  | Upper Bound | 820.85 |  |
|  | 5% Trimmed Mean | | 485.54 |  |
|  | Median | | 461.00 |  |
|  | Variance | | 1012008.470 |  |
|  | Std. Deviation | | 1005.986 |  |
|  | Minimum | | -1404 |  |
|  | Maximum | | 3957 |  |
|  | Range | | 5361 |  |
|  | Interquartile Range | | 1268 |  |
|  | Skewness | | 1.087 | .319 |
|  | Kurtosis | | 2.116 | .628 |

| **Statistics** | | |
| --- | --- | --- |
| Intake and Output (mL/day) | | |
| N | Valid | 56 |
|  | Missing | 2 |
| Percentiles | 25 | -187.50 |
|  | 50 | 461.00 |
|  | 75 | 1080.00 |

**Day 3**

| **Tests of Normality** | | | | | | |
| --- | --- | --- | --- | --- | --- | --- |
|  | Kolmogorov-Smirnov^a^ | | | Shapiro-Wilk | | |
|  | Statistic | df | Sig. | Statistic | df | Sig. |
| Intake and Output (mL/day) | .081 | 62 | .200^*^ | .982 | 62 | .513 |
| *. This is a lower bound of the true significance. | | | | | | |
| a. Lilliefors Significance Correction | | | | | | |

Patients

| **Descriptive Statistics** | | | | | |
| --- | --- | --- | --- | --- | --- |
|  | N | Minimum | Maximum | Mean | Std. Deviation |
| Intake and Output (mL/day) | 62 | -3289 | 2538 | 209.27 | 1116.017 |
| Valid N (listwise) | 62 |  |  |  |  |

Oliguria

| **Descriptive Statistics** | | | | | |
| --- | --- | --- | --- | --- | --- |
|  | N | Minimum | Maximum | Mean | Std. Deviation |
| Intake and Output (mL/day) | 11 | -3289 | 1688 | 397.45 | 1475.578 |
| Valid N (listwise) | 11 |  |  |  |  |

Non-oliguria

| **Descriptive Statistics** | | | | | |
| --- | --- | --- | --- | --- | --- |
|  | N | Minimum | Maximum | Mean | Std. Deviation |
| Intake and Output (mL/day) | 51 | -1924 | 2538 | 168.69 | 1036.612 |
| Valid N (listwise) | 51 |  |  |  |  |

P value

| **Group Statistics** | | | | | |
| --- | --- | --- | --- | --- | --- |
|  | Oliguria | N | Mean | Std. Deviation | Std. Error Mean |
| Intake and Output (mL/day) | urine＜500 mL | 11 | 397.45 | 1475.578 | 444.904 |
|  | urine≧500 mL | 51 | 168.69 | 1036.612 | 145.155 |

| **Independent Samples Test** | | | | | | | | | | |
| --- | --- | --- | --- | --- | --- | --- | --- | --- | --- | --- |
|  | | Levene's Test for Equality of Variances | | t-test for Equality of Means | | | | | | |
|  |  | F | Sig. | t | df | Sig. (2-tailed) | Mean Difference | Std. Error Difference | 95% Confidence Interval of the Difference | |
|  |  |  |  |  |  |  |  |  | Lower | Upper |
| Intake and Output (mL/day) | Equal variances assumed | 1.458 | .232 | .613 | 60 | .542 | 228.768 | 372.921 | -517.185 | 974.721 |
|  | Equal variances not assumed |  |  | .489 | 12.215 | .634 | 228.768 | 467.984 | -788.898 | 1246.435 |

**Total average**

| **Tests of Normality** | | | | | | |
| --- | --- | --- | --- | --- | --- | --- |
|  | Kolmogorov-Smirnov^a^ | | | Shapiro-Wilk | | |
|  | Statistic | df | Sig. | Statistic | df | Sig. |
| Intake and Output (mL/day) | .094 | 75 | .095 | .959 | 75 | .015 |
| a. Lilliefors Significance Correction | | | | | | |

Patients

| **Descriptive Statistics** | | | | | |
| --- | --- | --- | --- | --- | --- |
|  | N | Minimum | Maximum | Mean | Std. Deviation |
| Intake and Output (mL/day) | 75 | -1434 | 3347 | 522.55 | 972.221 |
| Valid N (listwise) | 75 |  |  |  |  |

Oliguria

| **Descriptive Statistics** | | | | | |
| --- | --- | --- | --- | --- | --- |
|  | N | Minimum | Maximum | Mean | Std. Deviation |
| Intake and Output (mL/day) | 17 | -972 | 3347 | 1235.06 | 1303.136 |
| Valid N (listwise) | 17 |  |  |  |  |

Non-oliguria

| **Descriptive Statistics** | | | | | |
| --- | --- | --- | --- | --- | --- |
|  | N | Minimum | Maximum | Mean | Std. Deviation |
| Intake and Output (mL/day) | 58 | -1434 | 2526 | 313.71 | 744.749 |
| Valid N (listwise) | 58 |  |  |  |  |

P value

| **Group Statistics** | | | | | |
| --- | --- | --- | --- | --- | --- |
|  | Oliguria | N | Mean | Std. Deviation | Std. Error Mean |
| Intake and Output (mL/day) | urine＜500 mL | 17 | 1235.06 | 1303.136 | 316.057 |
|  | urine≧500 mL | 58 | 313.71 | 744.749 | 97.790 |

| **Independent Samples Test** | | | | | | | | | | |
| --- | --- | --- | --- | --- | --- | --- | --- | --- | --- | --- |
|  | | Levene's Test for Equality of Variances | | t-test for Equality of Means | | | | | | |
|  |  | F | Sig. | t | df | Sig. (2-tailed) | Mean Difference | Std. Error Difference | 95% Confidence Interval of the Difference | |
|  |  |  |  |  |  |  |  |  | Lower | Upper |
| Intake and Output (mL/day) | Equal variances assumed | 10.112 | .002 | 3.723 | 73 | .000 | 921.352 | 247.495 | 428.095 | 1414.609 |
|  | Equal variances not assumed |  |  | 2.785 | 19.161 | .012 | 921.352 | 330.840 | 229.290 | 1613.414 |

**Urine output (Data recorded on the day prior to the NIRS measurement)**

**Day 1**

| **Tests of Normality** | | | | | | |
| --- | --- | --- | --- | --- | --- | --- |
|  | Kolmogorov-Smirnov^a^ | | | Shapiro-Wilk | | |
|  | Statistic | df | Sig. | Statistic | df | Sig. |
| Urine output (mL/day) | .164 | 68 | .000 | .784 | 68 | .000 |
| a. Lilliefors Significance Correction | | | | | | |

Patients

| **Descriptives** | | | | |
| --- | --- | --- | --- | --- |
|  | | | Statistic | Std. Error |
| Urine output (mL/day) | Mean | | 781.32 | 96.759 |
|  | 95% Confidence Interval for Mean | Lower Bound | 588.19 |  |
|  |  | Upper Bound | 974.46 |  |
|  | 5% Trimmed Mean | | 707.22 |  |
|  | Median | | 600.00 |  |
|  | Variance | | 636638.521 |  |
|  | Std. Deviation | | 797.896 |  |
|  | Minimum | | 0 |  |
|  | Maximum | | 5100 |  |
|  | Range | | 5100 |  |
|  | Interquartile Range | | 995 |  |
|  | Skewness | | 2.549 | .291 |
|  | Kurtosis | | 11.556 | .574 |

| **Statistics** | | |
| --- | --- | --- |
| Urine output (mL/day) | | |
| N | Valid | 68 |
|  | Missing | 7 |
| Percentiles | 25 | 192.50 |
|  | 50 | 600.00 |
|  | 75 | 1187.50 |

Oliguria

| **Descriptives** | | | | |
| --- | --- | --- | --- | --- |
|  | | | Statistic | Std. Error |
| Urine output (mL/day) | Mean | | 161.25 | 63.920 |
|  | 95% Confidence Interval for Mean | Lower Bound | 25.01 |  |
|  |  | Upper Bound | 297.49 |  |
|  | 5% Trimmed Mean | | 124.17 |  |
|  | Median | | 70.00 |  |
|  | Variance | | 65371.667 |  |
|  | Std. Deviation | | 255.679 |  |
|  | Minimum | | 0 |  |
|  | Maximum | | 990 |  |
|  | Range | | 990 |  |
|  | Interquartile Range | | 238 |  |
|  | Skewness | | 2.577 | .564 |
|  | Kurtosis | | 7.560 | 1.091 |

| **Statistics** | | |
| --- | --- | --- |
| Urine output (mL/day) | | |
| N | Valid | 16 |
|  | Missing | 1 |
| Percentiles | 25 | .00 |
|  | 50 | 70.00 |
|  | 75 | 237.50 |

Non-oliguria

| **Descriptives** | | | | |
| --- | --- | --- | --- | --- |
|  | | | Statistic | Std. Error |
| Urine output (mL/day) | Mean | | 972.12 | 112.609 |
|  | 95% Confidence Interval for Mean | Lower Bound | 746.04 |  |
|  |  | Upper Bound | 1198.19 |  |
|  | 5% Trimmed Mean | | 899.62 |  |
|  | Median | | 880.00 |  |
|  | Variance | | 659401.320 |  |
|  | Std. Deviation | | 812.035 |  |
|  | Minimum | | 0 |  |
|  | Maximum | | 5100 |  |
|  | Range | | 5100 |  |
|  | Interquartile Range | | 913 |  |
|  | Skewness | | 2.653 | .330 |
|  | Kurtosis | | 12.063 | .650 |

| **Statistics** | | |
| --- | --- | --- |
| Urine output (mL/day) | | |
| N | Valid | 52 |
|  | Missing | 6 |
| Percentiles | 25 | 400.00 |
|  | 50 | 880.00 |
|  | 75 | 1312.50 |

**Day 2**

| **Tests of Normality** | | | | | | |
| --- | --- | --- | --- | --- | --- | --- |
|  | Kolmogorov-Smirnov^a^ | | | Shapiro-Wilk | | |
|  | Statistic | df | Sig. | Statistic | df | Sig. |
| Urine output (mL/day) | .110 | 73 | .029 | .919 | 73 | .000 |
| a. Lilliefors Significance Correction | | | | | | |

Patients

| **Descriptives** | | | | |
| --- | --- | --- | --- | --- |
|  | | | Statistic | Std. Error |
| Urine output (mL/day) | Mean | | 1071.16 | 98.801 |
|  | 95% Confidence Interval for Mean | Lower Bound | 874.21 |  |
|  |  | Upper Bound | 1268.12 |  |
|  | 5% Trimmed Mean | | 1006.16 |  |
|  | Median | | 880.00 |  |
|  | Variance | | 712593.417 |  |
|  | Std. Deviation | | 844.152 |  |
|  | Minimum | | 0 |  |
|  | Maximum | | 4100 |  |
|  | Range | | 4100 |  |
|  | Interquartile Range | | 1105 |  |
|  | Skewness | | 1.148 | .281 |
|  | Kurtosis | | 1.814 | .555 |

| **Statistics** | | |
| --- | --- | --- |
| Urine output (mL/day) | | |
| N | Valid | 73 |
|  | Missing | 2 |
| Percentiles | 25 | 460.00 |
|  | 50 | 880.00 |
|  | 75 | 1565.00 |

Oliguria

| **Descriptives** | | | | |
| --- | --- | --- | --- | --- |
|  | | | Statistic | Std. Error |
| Urine output (mL/day) | Mean | | 163.24 | 42.263 |
|  | 95% Confidence Interval for Mean | Lower Bound | 73.64 |  |
|  |  | Upper Bound | 252.83 |  |
|  | 5% Trimmed Mean | | 152.48 |  |
|  | Median | | 120.00 |  |
|  | Variance | | 30365.441 |  |
|  | Std. Deviation | | 174.257 |  |
|  | Minimum | | 0 |  |
|  | Maximum | | 520 |  |
|  | Range | | 520 |  |
|  | Interquartile Range | | 318 |  |
|  | Skewness | | .752 | .550 |
|  | Kurtosis | | -.709 | 1.063 |

| **Statistics** | | |
| --- | --- | --- |
| Urine output (mL/day) | | |
| N | Valid | 17 |
|  | Missing | 0 |
| Percentiles | 25 | .00 |
|  | 50 | 120.00 |
|  | 75 | 317.50 |

Non-oliguria

| **Descriptives** | | | | |
| --- | --- | --- | --- | --- |
|  | | | Statistic | Std. Error |
| Urine output (mL/day) | Mean | | 1346.79 | 102.806 |
|  | 95% Confidence Interval for Mean | Lower Bound | 1140.76 |  |
|  |  | Upper Bound | 1552.81 |  |
|  | 5% Trimmed Mean | | 1279.17 |  |
|  | Median | | 1150.00 |  |
|  | Variance | | 591873.117 |  |
|  | Std. Deviation | | 769.333 |  |
|  | Minimum | | 310 |  |
|  | Maximum | | 4100 |  |
|  | Range | | 3790 |  |
|  | Interquartile Range | | 960 |  |
|  | Skewness | | 1.419 | .319 |
|  | Kurtosis | | 2.591 | .628 |

| **Statistics** | | |
| --- | --- | --- |
| Urine output (mL/day) | | |
| N | Valid | 56 |
|  | Missing | 2 |
| Percentiles | 25 | 800.00 |
|  | 50 | 1150.00 |
|  | 75 | 1760.00 |

**Day 3**

| **Tests of Normality** | | | | | | |
| --- | --- | --- | --- | --- | --- | --- |
|  | Kolmogorov-Smirnov^a^ | | | Shapiro-Wilk | | |
|  | Statistic | df | Sig. | Statistic | df | Sig. |
| Urine output (mL/day) | .134 | 62 | .007 | .932 | 62 | .002 |
| a. Lilliefors Significance Correction | | | | | | |

Patients

| **Descriptives** | | | | |
| --- | --- | --- | --- | --- |
|  | | | Statistic | Std. Error |
| Urine output (mL/day) | Mean | | 1307.90 | 120.389 |
|  | 95% Confidence Interval for Mean | Lower Bound | 1067.17 |  |
|  |  | Upper Bound | 1548.63 |  |
|  | 5% Trimmed Mean | | 1241.85 |  |
|  | Median | | 1150.00 |  |
|  | Variance | | 898590.613 |  |
|  | Std. Deviation | | 947.940 |  |
|  | Minimum | | 0 |  |
|  | Maximum | | 4200 |  |
|  | Range | | 4200 |  |
|  | Interquartile Range | | 1210 |  |
|  | Skewness | | .953 | .304 |
|  | Kurtosis | | .923 | .599 |

| **Statistics** | | |
| --- | --- | --- |
| Urine output (mL/day) | | |
| N | Valid | 62 |
|  | Missing | 13 |
| Percentiles | 25 | 690.00 |
|  | 50 | 1150.00 |
|  | 75 | 1900.00 |

Oliguria

| **Descriptives** | | | | |
| --- | --- | --- | --- | --- |
|  | | | Statistic | Std. Error |
| Urine output (mL/day) | Mean | | 150.00 | 65.768 |
|  | 95% Confidence Interval for Mean | Lower Bound | 3.46 |  |
|  |  | Upper Bound | 296.54 |  |
|  | 5% Trimmed Mean | | 136.11 |  |
|  | Median | | 50.00 |  |
|  | Variance | | 47580.000 |  |
|  | Std. Deviation | | 218.128 |  |
|  | Minimum | | 0 |  |
|  | Maximum | | 550 |  |
|  | Range | | 550 |  |
|  | Interquartile Range | | 320 |  |
|  | Skewness | | 1.361 | .661 |
|  | Kurtosis | | .245 | 1.279 |

| **Statistics** | | |
| --- | --- | --- |
| Urine output (mL/day) | | |
| N | Valid | 11 |
|  | Missing | 6 |
| Percentiles | 25 | .00 |
|  | 50 | 50.00 |
|  | 75 | 320.00 |

Non-oliguria

| **Descriptives** | | | | |
| --- | --- | --- | --- | --- |
|  | | | Statistic | Std. Error |
| Urine output (mL/day) | Mean | | 1557.65 | 119.491 |
|  | 95% Confidence Interval for Mean | Lower Bound | 1317.64 |  |
|  |  | Upper Bound | 1797.65 |  |
|  | 5% Trimmed Mean | | 1484.51 |  |
|  | Median | | 1330.00 |  |
|  | Variance | | 728182.353 |  |
|  | Std. Deviation | | 853.336 |  |
|  | Minimum | | 530 |  |
|  | Maximum | | 4200 |  |
|  | Range | | 3670 |  |
|  | Interquartile Range | | 1010 |  |
|  | Skewness | | 1.288 | .333 |
|  | Kurtosis | | 1.345 | .656 |

| **Statistics** | | |
| --- | --- | --- |
| Urine output (mL/day) | | |
| N | Valid | 51 |
|  | Missing | 7 |
| Percentiles | 25 | 940.00 |
|  | 50 | 1330.00 |
|  | 75 | 1950.00 |

**Total average**

| **Tests of Normality** | | | | | | |
| --- | --- | --- | --- | --- | --- | --- |
|  | Kolmogorov-Smirnov^a^ | | | Shapiro-Wilk | | |
|  | Statistic | df | Sig. | Statistic | df | Sig. |
| Urine output (mL/day) | .104 | 75 | .045 | .906 | 75 | .000 |
| a. Lilliefors Significance Correction | | | | | | |

Patients

| **Descriptives** | | | | |
| --- | --- | --- | --- | --- |
|  | | | Statistic | Std. Error |
| Urine output (mL/day) | Mean | | 1049.97 | 92.556 |
|  | 95% Confidence Interval for Mean | Lower Bound | 865.55 |  |
|  |  | Upper Bound | 1234.39 |  |
|  | 5% Trimmed Mean | | 982.61 |  |
|  | Median | | 943.00 |  |
|  | Variance | | 642490.378 |  |
|  | Std. Deviation | | 801.555 |  |
|  | Minimum | | 0 |  |
|  | Maximum | | 4263 |  |
|  | Range | | 4263 |  |
|  | Interquartile Range | | 886 |  |
|  | Skewness | | 1.357 | .277 |
|  | Kurtosis | | 3.157 | .548 |

| **Statistics** | | |
| --- | --- | --- |
| Urine output (mL/day) | | |
| N | Valid | 75 |
|  | Missing | 0 |
| Percentiles | 25 | 547.00 |
|  | 50 | 943.00 |
|  | 75 | 1433.00 |

Oliguria

| **Descriptives** | | | | |
| --- | --- | --- | --- | --- |
|  | | | Statistic | Std. Error |
| Urine output (mL/day) | Mean | | 154.76 | 39.609 |
|  | 95% Confidence Interval for Mean | Lower Bound | 70.80 |  |
|  |  | Upper Bound | 238.73 |  |
|  | 5% Trimmed Mean | | 145.13 |  |
|  | Median | | 100.00 |  |
|  | Variance | | 26671.316 |  |
|  | Std. Deviation | | 163.314 |  |
|  | Minimum | | 0 |  |
|  | Maximum | | 483 |  |
|  | Range | | 483 |  |
|  | Interquartile Range | | 305 |  |
|  | Skewness | | .794 | .550 |
|  | Kurtosis | | -.693 | 1.063 |

| **Statistics** | | |
| --- | --- | --- |
| Urine output (mL/day) | | |
| N | Valid | 17 |
|  | Missing | 0 |
| Percentiles | 25 | 1.50 |
|  | 50 | 100.00 |
|  | 75 | 306.00 |

Non-oliguria

| **Descriptives** | | | | |
| --- | --- | --- | --- | --- |
|  | | | Statistic | Std. Error |
| Urine output (mL/day) | Mean | | 1312.36 | 94.463 |
|  | 95% Confidence Interval for Mean | Lower Bound | 1123.20 |  |
|  |  | Upper Bound | 1501.52 |  |
|  | 5% Trimmed Mean | | 1230.21 |  |
|  | Median | | 1105.00 |  |
|  | Variance | | 517554.340 |  |
|  | Std. Deviation | | 719.412 |  |
|  | Minimum | | 537 |  |
|  | Maximum | | 4263 |  |
|  | Range | | 3726 |  |
|  | Interquartile Range | | 732 |  |
|  | Skewness | | 1.921 | .314 |
|  | Kurtosis | | 4.866 | .618 |

| **Statistics** | | |
| --- | --- | --- |
| Urine output (mL/day) | | |
| N | Valid | 58 |
|  | Missing | 0 |
| Percentiles | 25 | 854.25 |
|  | 50 | 1105.00 |
|  | 75 | 1585.75 |

**Intravascular fluid administration (Data recorded on the day prior to the NIRS measurement)**

**Day 1**

| **Tests of Normality** | | | | | | |
| --- | --- | --- | --- | --- | --- | --- |
|  | Kolmogorov-Smirnov^a^ | | | Shapiro-Wilk | | |
|  | Statistic | df | Sig. | Statistic | df | Sig. |
| Intravascular fluid administration (mL/day) | .131 | 68 | .006 | .866 | 68 | .000 |
| a. Lilliefors Significance Correction | | | | | | |

Patients

| **Descriptives** | | | | |
| --- | --- | --- | --- | --- |
|  | | | Statistic | Std. Error |
| Intravascular fluid administration (mL/day) | Mean | | 1245.72 | 114.669 |
|  | 95% Confidence Interval for Mean | Lower Bound | 1016.84 |  |
|  |  | Upper Bound | 1474.60 |  |
|  | 5% Trimmed Mean | | 1155.47 |  |
|  | Median | | 973.00 |  |
|  | Variance | | 894129.070 |  |
|  | Std. Deviation | | 945.584 |  |
|  | Minimum | | 100 |  |
|  | Maximum | | 4330 |  |
|  | Range | | 4230 |  |
|  | Interquartile Range | | 1050 |  |
|  | Skewness | | 1.468 | .291 |
|  | Kurtosis | | 2.162 | .574 |

| **Statistics** | | |
| --- | --- | --- |
| Intravascular fluid administration (mL/day) | | |
| N | Valid | 68 |
|  | Missing | 7 |
| Percentiles | 25 | 570.00 |
|  | 50 | 973.00 |
|  | 75 | 1620.25 |

Oliguria

| **Descriptives** | | | | |
| --- | --- | --- | --- | --- |
|  | | | Statistic | Std. Error |
| Intravascular fluid administration (mL/day) | Mean | | 1863.88 | 326.160 |
|  | 95% Confidence Interval for Mean | Lower Bound | 1168.68 |  |
|  |  | Upper Bound | 2559.07 |  |
|  | 5% Trimmed Mean | | 1824.86 |  |
|  | Median | | 1726.50 |  |
|  | Variance | | 1702088.117 |  |
|  | Std. Deviation | | 1304.641 |  |
|  | Minimum | | 100 |  |
|  | Maximum | | 4330 |  |
|  | Range | | 4230 |  |
|  | Interquartile Range | | 1522 |  |
|  | Skewness | | .615 | .564 |
|  | Kurtosis | | -.368 | 1.091 |

| **Statistics** | | |
| --- | --- | --- |
| Intravascular fluid administration (mL/day) | | |
| N | Valid | 16 |
|  | Missing | 1 |
| Percentiles | 25 | 919.75 |
|  | 50 | 1726.50 |
|  | 75 | 2441.50 |

Non-oliguria

| **Descriptives** | | | | |
| --- | --- | --- | --- | --- |
|  | | | Statistic | Std. Error |
| Intravascular fluid administration (mL/day) | Mean | | 1055.52 | 99.736 |
|  | 95% Confidence Interval for Mean | Lower Bound | 855.29 |  |
|  |  | Upper Bound | 1255.75 |  |
|  | 5% Trimmed Mean | | 984.01 |  |
|  | Median | | 922.50 |  |
|  | Variance | | 517260.921 |  |
|  | Std. Deviation | | 719.209 |  |
|  | Minimum | | 186 |  |
|  | Maximum | | 3430 |  |
|  | Range | | 3244 |  |
|  | Interquartile Range | | 892 |  |
|  | Skewness | | 1.486 | .330 |
|  | Kurtosis | | 2.764 | .650 |

| **Statistics** | | |
| --- | --- | --- |
| Intravascular fluid administration (mL/day) | | |
| N | Valid | 52 |
|  | Missing | 6 |
| Percentiles | 25 | 526.00 |
|  | 50 | 922.50 |
|  | 75 | 1417.50 |

**Day 2**

| **Tests of Normality** | | | | | | |
| --- | --- | --- | --- | --- | --- | --- |
|  | Kolmogorov-Smirnov^a^ | | | Shapiro-Wilk | | |
|  | Statistic | df | Sig. | Statistic | df | Sig. |
| Intravascular fluid administration (mL/day) | .154 | 73 | .000 | .872 | 73 | .000 |
| a. Lilliefors Significance Correction | | | | | | |

Patients

| **Descriptives** | | | | |
| --- | --- | --- | --- | --- |
|  | | | Statistic | Std. Error |
| Intravascular fluid administration (mL/day) | Mean | | 1653.95 | 133.786 |
|  | 95% Confidence Interval for Mean | Lower Bound | 1387.25 |  |
|  |  | Upper Bound | 1920.64 |  |
|  | 5% Trimmed Mean | | 1550.53 |  |
|  | Median | | 1440.00 |  |
|  | Variance | | 1306608.664 |  |
|  | Std. Deviation | | 1143.070 |  |
|  | Minimum | | 80 |  |
|  | Maximum | | 6101 |  |
|  | Range | | 6021 |  |
|  | Interquartile Range | | 1115 |  |
|  | Skewness | | 1.607 | .281 |
|  | Kurtosis | | 3.405 | .555 |

| **Statistics** | | |
| --- | --- | --- |
| Intravascular fluid administration (mL/day) | | |
| N | Valid | 73 |
|  | Missing | 2 |
| Percentiles | 25 | 902.00 |
|  | 50 | 1440.00 |
|  | 75 | 2017.00 |

Oliguria

| **Descriptives** | | | | |
| --- | --- | --- | --- | --- |
|  | | | Statistic | Std. Error |
| Intravascular fluid administration (mL/day) | Mean | | 2542.59 | 373.882 |
|  | 95% Confidence Interval for Mean | Lower Bound | 1749.99 |  |
|  |  | Upper Bound | 3335.18 |  |
|  | 5% Trimmed Mean | | 2475.04 |  |
|  | Median | | 2074.00 |  |
|  | Variance | | 2376393.007 |  |
|  | Std. Deviation | | 1541.555 |  |
|  | Minimum | | 200 |  |
|  | Maximum | | 6101 |  |
|  | Range | | 5901 |  |
|  | Interquartile Range | | 2163 |  |
|  | Skewness | | .970 | .550 |
|  | Kurtosis | | .629 | 1.063 |

| **Statistics** | | |
| --- | --- | --- |
| Intravascular fluid administration (mL/day) | | |
| N | Valid | 17 |
|  | Missing | 0 |
| Percentiles | 25 | 1412.50 |
|  | 50 | 2074.00 |
|  | 75 | 3575.00 |

Non-oliguria

| **Descriptives** | | | | |
| --- | --- | --- | --- | --- |
|  | | | Statistic | Std. Error |
| Intravascular fluid administration (mL/day) | Mean | | 1384.18 | 111.881 |
|  | 95% Confidence Interval for Mean | Lower Bound | 1159.96 |  |
|  |  | Upper Bound | 1608.39 |  |
|  | 5% Trimmed Mean | | 1315.31 |  |
|  | Median | | 1350.00 |  |
|  | Variance | | 700973.095 |  |
|  | Std. Deviation | | 837.241 |  |
|  | Minimum | | 80 |  |
|  | Maximum | | 4137 |  |
|  | Range | | 4057 |  |
|  | Interquartile Range | | 1123 |  |
|  | Skewness | | 1.187 | .319 |
|  | Kurtosis | | 2.067 | .628 |

| **Statistics** | | |
| --- | --- | --- |
| Intravascular fluid administration (mL/day) | | |
| N | Valid | 56 |
|  | Missing | 2 |
| Percentiles | 25 | 784.50 |
|  | 50 | 1350.00 |
|  | 75 | 1907.00 |

**Day 3**

| **Tests of Normality** | | | | | | |
| --- | --- | --- | --- | --- | --- | --- |
|  | Kolmogorov-Smirnov^a^ | | | Shapiro-Wilk | | |
|  | Statistic | df | Sig. | Statistic | df | Sig. |
| Intravascular fluid administration (mL/day) | .153 | 62 | .001 | .893 | 62 | .000 |
| a. Lilliefors Significance Correction | | | | | | |

Patients

| **Descriptives** | | | | |
| --- | --- | --- | --- | --- |
|  | | | Statistic | Std. Error |
| Intravascular fluid administration (mL/day) | Mean | | 1108.79 | 102.001 |
|  | 95% Confidence Interval for Mean | Lower Bound | 904.83 |  |
|  |  | Upper Bound | 1312.75 |  |
|  | 5% Trimmed Mean | | 1051.16 |  |
|  | Median | | 960.00 |  |
|  | Variance | | 645061.021 |  |
|  | Std. Deviation | | 803.157 |  |
|  | Minimum | | 80 |  |
|  | Maximum | | 4430 |  |
|  | Range | | 4350 |  |
|  | Interquartile Range | | 1144 |  |
|  | Skewness | | 1.417 | .304 |
|  | Kurtosis | | 3.401 | .599 |

| **Statistics** | | |
| --- | --- | --- |
| Intravascular fluid administration (mL/day) | | |
| N | Valid | 62 |
|  | Missing | 13 |
| Percentiles | 25 | 480.00 |
|  | 50 | 960.00 |
|  | 75 | 1624.25 |

Oliguria

| **Descriptives** | | | | |
| --- | --- | --- | --- | --- |
|  | | | Statistic | Std. Error |
| Intravascular fluid administration (mL/day) | Mean | | 1514.18 | 247.743 |
|  | 95% Confidence Interval for Mean | Lower Bound | 962.18 |  |
|  |  | Upper Bound | 2066.19 |  |
|  | 5% Trimmed Mean | | 1503.59 |  |
|  | Median | | 1693.00 |  |
|  | Variance | | 675142.764 |  |
|  | Std. Deviation | | 821.671 |  |
|  | Minimum | | 480 |  |
|  | Maximum | | 2739 |  |
|  | Range | | 2259 |  |
|  | Interquartile Range | | 1542 |  |
|  | Skewness | | .021 | .661 |
|  | Kurtosis | | -1.767 | 1.279 |

| **Statistics** | | |
| --- | --- | --- |
| Intravascular fluid administration (mL/day) | | |
| N | Valid | 11 |
|  | Missing | 6 |
| Percentiles | 25 | 624.00 |
|  | 50 | 1693.00 |
|  | 75 | 2166.00 |

Non-oliguria

| **Descriptives** | | | | |
| --- | --- | --- | --- | --- |
|  | | | Statistic | Std. Error |
| Intravascular fluid administration (mL/day) | Mean | | 1021.35 | 109.185 |
|  | 95% Confidence Interval for Mean | Lower Bound | 802.05 |  |
|  |  | Upper Bound | 1240.66 |  |
|  | 5% Trimmed Mean | | 953.54 |  |
|  | Median | | 940.00 |  |
|  | Variance | | 607992.393 |  |
|  | Std. Deviation | | 779.739 |  |
|  | Minimum | | 80 |  |
|  | Maximum | | 4430 |  |
|  | Range | | 4350 |  |
|  | Interquartile Range | | 970 |  |
|  | Skewness | | 1.858 | .333 |
|  | Kurtosis | | 6.029 | .656 |

| **Statistics** | | |
| --- | --- | --- |
| Intravascular fluid administration (mL/day) | | |
| N | Valid | 51 |
|  | Missing | 7 |
| Percentiles | 25 | 450.00 |
|  | 50 | 940.00 |
|  | 75 | 1420.00 |

**Total average**

| **Tests of Normality** | | | | | | |
| --- | --- | --- | --- | --- | --- | --- |
|  | Kolmogorov-Smirnov^a^ | | | Shapiro-Wilk | | |
|  | Statistic | df | Sig. | Statistic | df | Sig. |
| Intravascular fluid administration (mL/day) | .159 | 75 | .000 | .902 | 75 | .000 |
| a. Lilliefors Significance Correction | | | | | | |

Patients

| **Descriptives** | | | | |
| --- | --- | --- | --- | --- |
|  | | | Statistic | Std. Error |
| Intravascular fluid administration (mL/day) | Mean | | 1383.87 | 96.012 |
|  | 95% Confidence Interval for Mean | Lower Bound | 1192.56 |  |
|  |  | Upper Bound | 1575.17 |  |
|  | 5% Trimmed Mean | | 1322.92 |  |
|  | Median | | 1105.00 |  |
|  | Variance | | 691367.117 |  |
|  | Std. Deviation | | 831.485 |  |
|  | Minimum | | 170 |  |
|  | Maximum | | 3763 |  |
|  | Range | | 3593 |  |
|  | Interquartile Range | | 1095 |  |
|  | Skewness | | 1.135 | .277 |
|  | Kurtosis | | .892 | .548 |

| **Statistics** | | |
| --- | --- | --- |
| Intravascular fluid administration (mL/day) | | |
| N | Valid | 75 |
|  | Missing | 0 |
| Percentiles | 25 | 767.00 |
|  | 50 | 1105.00 |
|  | 75 | 1862.00 |

Oliguria

| **Descriptives** | | | | |
| --- | --- | --- | --- | --- |
|  | | | Statistic | Std. Error |
| Intravascular fluid administration (mL/day) | Mean | | 2142.76 | 256.027 |
|  | 95% Confidence Interval for Mean | Lower Bound | 1600.01 |  |
|  |  | Upper Bound | 2685.52 |  |
|  | 5% Trimmed Mean | | 2157.35 |  |
|  | Median | | 1926.00 |  |
|  | Variance | | 1114343.941 |  |
|  | Std. Deviation | | 1055.625 |  |
|  | Minimum | | 260 |  |
|  | Maximum | | 3763 |  |
|  | Range | | 3503 |  |
|  | Interquartile Range | | 1744 |  |
|  | Skewness | | .082 | .550 |
|  | Kurtosis | | -.900 | 1.063 |

| **Statistics** | | |
| --- | --- | --- |
| Intravascular fluid administration (mL/day) | | |
| N | Valid | 17 |
|  | Missing | 0 |
| Percentiles | 25 | 1348.50 |
|  | 50 | 1926.00 |
|  | 75 | 3092.00 |

Non-oliguria

| **Descriptives** | | | | |
| --- | --- | --- | --- | --- |
|  | | | Statistic | Std. Error |
| Intravascular fluid administration (mL/day) | Mean | | 1161.43 | 79.074 |
|  | 95% Confidence Interval for Mean | Lower Bound | 1003.09 |  |
|  |  | Upper Bound | 1319.77 |  |
|  | 5% Trimmed Mean | | 1121.57 |  |
|  | Median | | 1069.00 |  |
|  | Variance | | 362652.916 |  |
|  | Std. Deviation | | 602.207 |  |
|  | Minimum | | 170 |  |
|  | Maximum | | 2772 |  |
|  | Range | | 2602 |  |
|  | Interquartile Range | | 765 |  |
|  | Skewness | | 1.001 | .314 |
|  | Kurtosis | | .766 | .618 |

| **Statistics** | | |
| --- | --- | --- |
| Intravascular fluid administration (mL/day) | | |
| N | Valid | 58 |
|  | Missing | 0 |
| Percentiles | 25 | 722.00 |
|  | 50 | 1069.00 |
|  | 75 | 1486.75 |

**Diagnosis**

**Pulmonary infection**

Patients

| **Pulmonary infection** | | | | | |
| --- | --- | --- | --- | --- | --- |
|  | | Frequency | Percent | Valid Percent | Cumulative Percent |
| Valid | No | 26 | 34.7 | 34.7 | 34.7 |
|  | Yes | 49 | 65.3 | 65.3 | 100.0 |
|  | Total | 75 | 100.0 | 100.0 |  |

Oliguria

| **Pulmonary infection** | | | | | |
| --- | --- | --- | --- | --- | --- |
|  | | Frequency | Percent | Valid Percent | Cumulative Percent |
| Valid | No | 3 | 17.6 | 17.6 | 17.6 |
|  | Yes | 14 | 82.4 | 82.4 | 100.0 |
|  | Total | 17 | 100.0 | 100.0 |  |

Non-oliguria

| **Pulmonary infection** | | | | | |
| --- | --- | --- | --- | --- | --- |
|  | | Frequency | Percent | Valid Percent | Cumulative Percent |
| Valid | No | 23 | 39.7 | 39.7 | 39.7 |
|  | Yes | 35 | 60.3 | 60.3 | 100.0 |
|  | Total | 58 | 100.0 | 100.0 |  |

P value

| **Oliguria * Pulmonary infection Crosstabulation** | | | | |
| --- | --- | --- | --- | --- |
| Count | | | | |
|  | | Pulmonary infection | | Total |
|  |  | No | Yes |  |
| Oliguria | urine＜500 mL | 3 | 14 | 17 |
|  | urine≧500 mL | 23 | 35 | 58 |
| Total | | 26 | 49 | 75 |

| **Chi-Square Tests** | | | | | |
| --- | --- | --- | --- | --- | --- |
|  | Value | df | Asymp. Sig. (2-sided) | Exact Sig. (2-sided) | Exact Sig. (1-sided) |
| Pearson Chi-Square | 2.811^a^ | 1 | .094 |  |  |
| Continuity Correction^b^ | 1.924 | 1 | .165 |  |  |
| Likelihood Ratio | 3.056 | 1 | .080 |  |  |
| Fisher's Exact Test |  |  |  | .147 | .080 |
| Linear-by-Linear Association | 2.774 | 1 | .096 |  |  |
| N of Valid Cases | 75 |  |  |  |  |
| a. 0 cells (.0%) have expected count less than 5. The minimum expected count is 5.89. | | | | | |
| b. Computed only for a 2x2 table | | | | | |

**Urinary tract infection**

Patients

| **Urinary tract infection** | | | | | |
| --- | --- | --- | --- | --- | --- |
|  | | Frequency | Percent | Valid Percent | Cumulative Percent |
| Valid | No | 40 | 53.3 | 53.3 | 53.3 |
|  | Yes | 35 | 46.7 | 46.7 | 100.0 |
|  | Total | 75 | 100.0 | 100.0 |  |

Oliguria

| **Urinary tract infection** | | | | | |
| --- | --- | --- | --- | --- | --- |
|  | | Frequency | Percent | Valid Percent | Cumulative Percent |
| Valid | No | 9 | 52.9 | 52.9 | 52.9 |
|  | Yes | 8 | 47.1 | 47.1 | 100.0 |
|  | Total | 17 | 100.0 | 100.0 |  |

Non-oliguria

| **Urinary tract infection** | | | | | |
| --- | --- | --- | --- | --- | --- |
|  | | Frequency | Percent | Valid Percent | Cumulative Percent |
| Valid | No | 31 | 53.4 | 53.4 | 53.4 |
|  | Yes | 27 | 46.6 | 46.6 | 100.0 |
|  | Total | 58 | 100.0 | 100.0 |  |

P value

| **Oliguria * Urinary tract infection Crosstabulation** | | | | |
| --- | --- | --- | --- | --- |
| Count | | | | |
|  | | Urinary tract infection | | Total |
|  |  | No | Yes |  |
| Oliguria | urine＜500 mL | 9 | 8 | 17 |
|  | urine≧500 mL | 31 | 27 | 58 |
| Total | | 40 | 35 | 75 |

| **Chi-Square Tests** | | | | | |
| --- | --- | --- | --- | --- | --- |
|  | Value | df | Asymp. Sig. (2-sided) | Exact Sig. (2-sided) | Exact Sig. (1-sided) |
| Pearson Chi-Square | .001^a^ | 1 | .971 |  |  |
| Continuity Correction^b^ | .000 | 1 | 1.000 |  |  |
| Likelihood Ratio | .001 | 1 | .971 |  |  |
| Fisher's Exact Test |  |  |  | 1.000 | .593 |
| Linear-by-Linear Association | .001 | 1 | .971 |  |  |
| N of Valid Cases | 75 |  |  |  |  |
| a. 0 cells (.0%) have expected count less than 5. The minimum expected count is 7.93. | | | | | |
| b. Computed only for a 2x2 table | | | | | |

**Hepatic or biliary tract infection**

Patients

| **Hepatic or biliary tract infection** | | | | | |
| --- | --- | --- | --- | --- | --- |
|  | | Frequency | Percent | Valid Percent | Cumulative Percent |
| Valid | No | 67 | 89.3 | 89.3 | 89.3 |
|  | Yes | 8 | 10.7 | 10.7 | 100.0 |
|  | Total | 75 | 100.0 | 100.0 |  |

Oliguria

| **Hepatic or biliary tract infection** | | | | | |
| --- | --- | --- | --- | --- | --- |
|  | | Frequency | Percent | Valid Percent | Cumulative Percent |
| Valid | No | 17 | 100.0 | 100.0 | 100.0 |

Non-oliguria

| **Hepatic or biliary tract infection** | | | | | |
| --- | --- | --- | --- | --- | --- |
|  | | Frequency | Percent | Valid Percent | Cumulative Percent |
| Valid | No | 50 | 86.2 | 86.2 | 86.2 |
|  | Yes | 8 | 13.8 | 13.8 | 100.0 |
|  | Total | 58 | 100.0 | 100.0 |  |

P value

| **Oliguria * Hepatic or biliary tract infection Crosstabulation** | | | | |
| --- | --- | --- | --- | --- |
| Count | | | | |
|  | | Hepatic or biliary tract infection | | Total |
|  |  | No | Yes |  |
| Oliguria | urine＜500 mL | 17 | 0 | 17 |
|  | urine≧500 mL | 50 | 8 | 58 |
| Total | | 67 | 8 | 75 |

| **Chi-Square Tests** | | | | | |
| --- | --- | --- | --- | --- | --- |
|  | Value | df | Asymp. Sig. (2-sided) | Exact Sig. (2-sided) | Exact Sig. (1-sided) |
| Pearson Chi-Square | 2.625^a^ | 1 | .105 |  |  |
| Continuity Correction^b^ | 1.377 | 1 | .241 |  |  |
| Likelihood Ratio | 4.385 | 1 | .036 |  |  |
| Fisher's Exact Test |  |  |  | .186 | .114 |
| Linear-by-Linear Association | 2.590 | 1 | .108 |  |  |
| N of Valid Cases | 75 |  |  |  |  |
| a. 1 cells (25.0%) have expected count less than 5. The minimum expected count is 1.81. | | | | | |
| b. Computed only for a 2x2 table | | | | | |

**Spontaneous bacteria peritonitis**

Patients

| **Spontaneous bacteria peritonitis** | | | | | |
| --- | --- | --- | --- | --- | --- |
|  | | Frequency | Percent | Valid Percent | Cumulative Percent |
| Valid | No | 74 | 98.7 | 98.7 | 98.7 |
|  | Yes | 1 | 1.3 | 1.3 | 100.0 |
|  | Total | 75 | 100.0 | 100.0 |  |

Oliguria

| **Spontaneous bacteria peritonitis** | | | | | |
| --- | --- | --- | --- | --- | --- |
|  | | Frequency | Percent | Valid Percent | Cumulative Percent |
| Valid | No | 17 | 100.0 | 100.0 | 100.0 |

Non-oliguria

| **Spontaneous bacteria peritonitis** | | | | | |
| --- | --- | --- | --- | --- | --- |
|  | | Frequency | Percent | Valid Percent | Cumulative Percent |
| Valid | No | 57 | 98.3 | 98.3 | 98.3 |
|  | Yes | 1 | 1.7 | 1.7 | 100.0 |
|  | Total | 58 | 100.0 | 100.0 |  |

P value

| **Oliguria * Spontaneous bacteria peritonitis Crosstabulation** | | | | |
| --- | --- | --- | --- | --- |
| Count | | | | |
|  | | Spontaneous bacteria peritonitis | | Total |
|  |  | No | Yes |  |
| Oliguria | urine＜500 mL | 17 | 0 | 17 |
|  | urine≧500 mL | 57 | 1 | 58 |
| Total | | 74 | 1 | 75 |

| **Chi-Square Tests** | | | | | |
| --- | --- | --- | --- | --- | --- |
|  | Value | df | Asymp. Sig. (2-sided) | Exact Sig. (2-sided) | Exact Sig. (1-sided) |
| Pearson Chi-Square | .297^a^ | 1 | .586 |  |  |
| Continuity Correction^b^ | .000 | 1 | 1.000 |  |  |
| Likelihood Ratio | .518 | 1 | .472 |  |  |
| Fisher's Exact Test |  |  |  | 1.000 | .773 |
| Linear-by-Linear Association | .293 | 1 | .588 |  |  |
| N of Valid Cases | 75 |  |  |  |  |
| a. 2 cells (50.0%) have expected count less than 5. The minimum expected count is .23. | | | | | |
| b. Computed only for a 2x2 table | | | | | |

**Pelvic infection**

Patients

| **Pelvic infection** | | | | | |
| --- | --- | --- | --- | --- | --- |
|  | | Frequency | Percent | Valid Percent | Cumulative Percent |
| Valid | No | 74 | 98.7 | 98.7 | 98.7 |
|  | Yes | 1 | 1.3 | 1.3 | 100.0 |
|  | Total | 75 | 100.0 | 100.0 |  |

Oliguria

| **Pelvic infection** | | | | | |
| --- | --- | --- | --- | --- | --- |
|  | | Frequency | Percent | Valid Percent | Cumulative Percent |
| Valid | No | 17 | 100.0 | 100.0 | 100.0 |

Non-oliguria

| **Pelvic infection** | | | | | |
| --- | --- | --- | --- | --- | --- |
|  | | Frequency | Percent | Valid Percent | Cumulative Percent |
| Valid | No | 57 | 98.3 | 98.3 | 98.3 |
|  | Yes | 1 | 1.7 | 1.7 | 100.0 |
|  | Total | 58 | 100.0 | 100.0 |  |

P value

| **Oliguria * Pelvic infection Crosstabulation** | | | | |
| --- | --- | --- | --- | --- |
| Count | | | | |
|  | | Pelvic infection | | Total |
|  |  | No | Yes |  |
| Oliguria | urine＜500 mL | 17 | 0 | 17 |
|  | urine≧500 mL | 57 | 1 | 58 |
| Total | | 74 | 1 | 75 |

| **Chi-Square Tests** | | | | | |
| --- | --- | --- | --- | --- | --- |
|  | Value | df | Asymp. Sig. (2-sided) | Exact Sig. (2-sided) | Exact Sig. (1-sided) |
| Pearson Chi-Square | .297^a^ | 1 | .586 |  |  |
| Continuity Correction^b^ | .000 | 1 | 1.000 |  |  |
| Likelihood Ratio | .518 | 1 | .472 |  |  |
| Fisher's Exact Test |  |  |  | 1.000 | .773 |
| Linear-by-Linear Association | .293 | 1 | .588 |  |  |
| N of Valid Cases | 75 |  |  |  |  |
| a. 2 cells (50.0%) have expected count less than 5. The minimum expected count is .23. | | | | | |
| b. Computed only for a 2x2 table | | | | | |

**Cellulitis**

Patients

| **Cellulitis** | | | | | |
| --- | --- | --- | --- | --- | --- |
|  | | Frequency | Percent | Valid Percent | Cumulative Percent |
| Valid | No | 71 | 94.7 | 94.7 | 94.7 |
|  | Yes | 4 | 5.3 | 5.3 | 100.0 |
|  | Total | 75 | 100.0 | 100.0 |  |

Oliguria

| **Cellulitis** | | | | | |
| --- | --- | --- | --- | --- | --- |
|  | | Frequency | Percent | Valid Percent | Cumulative Percent |
| Valid | No | 16 | 94.1 | 94.1 | 94.1 |
|  | Yes | 1 | 5.9 | 5.9 | 100.0 |
|  | Total | 17 | 100.0 | 100.0 |  |

Non-oliguria

| **Cellulitis** | | | | | |
| --- | --- | --- | --- | --- | --- |
|  | | Frequency | Percent | Valid Percent | Cumulative Percent |
| Valid | No | 55 | 94.8 | 94.8 | 94.8 |
|  | Yes | 3 | 5.2 | 5.2 | 100.0 |
|  | Total | 58 | 100.0 | 100.0 |  |

P value

| **Oliguria * Cellulitis Crosstabulation** | | | | |
| --- | --- | --- | --- | --- |
| Count | | | | |
|  | | Cellulitis | | Total |
|  |  | No | Yes |  |
| Oliguria | urine＜500 mL | 16 | 1 | 17 |
|  | urine≧500 mL | 55 | 3 | 58 |
| Total | | 71 | 4 | 75 |

| **Chi-Square Tests** | | | | | |
| --- | --- | --- | --- | --- | --- |
|  | Value | df | Asymp. Sig. (2-sided) | Exact Sig. (2-sided) | Exact Sig. (1-sided) |
| Pearson Chi-Square | .013^a^ | 1 | .909 |  |  |
| Continuity Correction^b^ | .000 | 1 | 1.000 |  |  |
| Likelihood Ratio | .013 | 1 | .910 |  |  |
| Fisher's Exact Test |  |  |  | 1.000 | .651 |
| Linear-by-Linear Association | .013 | 1 | .909 |  |  |
| N of Valid Cases | 75 |  |  |  |  |
| a. 2 cells (50.0%) have expected count less than 5. The minimum expected count is .91. | | | | | |
| b. Computed only for a 2x2 table | | | | | |

**Other**

Patients

| **Other** | | | | | |
| --- | --- | --- | --- | --- | --- |
|  | | Frequency | Percent | Valid Percent | Cumulative Percent |
| Valid | No | 72 | 96.0 | 96.0 | 96.0 |
|  | Yes | 3 | 4.0 | 4.0 | 100.0 |
|  | Total | 75 | 100.0 | 100.0 |  |

Oliguria

| **Other** | | | | | |
| --- | --- | --- | --- | --- | --- |
|  | | Frequency | Percent | Valid Percent | Cumulative Percent |
| Valid | No | 17 | 100.0 | 100.0 | 100.0 |

Non-oliguria

| **Other** | | | | | |
| --- | --- | --- | --- | --- | --- |
|  | | Frequency | Percent | Valid Percent | Cumulative Percent |
| Valid | No | 55 | 94.8 | 94.8 | 94.8 |
|  | Yes | 3 | 5.2 | 5.2 | 100.0 |
|  | Total | 58 | 100.0 | 100.0 |  |

P value

| **Oliguria * Other Crosstabulation** | | | | |
| --- | --- | --- | --- | --- |
| Count | | | | |
|  | | Other | | Total |
|  |  | No | Yes |  |
| Oliguria | urine＜500 mL | 17 | 0 | 17 |
|  | urine≧500 mL | 55 | 3 | 58 |
| Total | | 72 | 3 | 75 |

| **Chi-Square Tests** | | | | | |
| --- | --- | --- | --- | --- | --- |
|  | Value | df | Asymp. Sig. (2-sided) | Exact Sig. (2-sided) | Exact Sig. (1-sided) |
| Pearson Chi-Square | .916^a^ | 1 | .339 |  |  |
| Continuity Correction^b^ | .064 | 1 | .800 |  |  |
| Likelihood Ratio | 1.579 | 1 | .209 |  |  |
| Fisher's Exact Test |  |  |  | 1.000 | .457 |
| Linear-by-Linear Association | .904 | 1 | .342 |  |  |
| N of Valid Cases | 75 |  |  |  |  |
| a. 2 cells (50.0%) have expected count less than 5. The minimum expected count is .68. | | | | | |
| b. Computed only for a 2x2 table | | | | | |

**ICU survivor**

Patients

| **ICU survivor** | | | | | |
| --- | --- | --- | --- | --- | --- |
|  | | Frequency | Percent | Valid Percent | Cumulative Percent |
| Valid | ICU non-survivor | 16 | 21.3 | 21.3 | 21.3 |
|  | ICU survivor | 59 | 78.7 | 78.7 | 100.0 |
|  | Total | 75 | 100.0 | 100.0 |  |

Oliguria

| **ICU survivor** | | | | | |
| --- | --- | --- | --- | --- | --- |
|  | | Frequency | Percent | Valid Percent | Cumulative Percent |
| Valid | ICU non-survivor | 9 | 52.9 | 52.9 | 52.9 |
|  | ICU survivor | 8 | 47.1 | 47.1 | 100.0 |
|  | Total | 17 | 100.0 | 100.0 |  |

Non-oliguria

| **ICU survivor** | | | | | |
| --- | --- | --- | --- | --- | --- |
|  | | Frequency | Percent | Valid Percent | Cumulative Percent |
| Valid | ICU non-survivor | 7 | 12.1 | 12.1 | 12.1 |
|  | ICU survivor | 51 | 87.9 | 87.9 | 100.0 |
|  | Total | 58 | 100.0 | 100.0 |  |

P value

| **Oliguria * ICU survivor Crosstabulation** | | | | |
| --- | --- | --- | --- | --- |
| Count | | | | |
|  | | ICU survivor | | Total |
|  |  | ICU non-survivor | ICU survivor |  |
| Oliguria | urine＜500 mL | 9 | 8 | 17 |
|  | urine≧500 mL | 7 | 51 | 58 |
| Total | | 16 | 59 | 75 |

| **Chi-Square Tests** | | | | | |
| --- | --- | --- | --- | --- | --- |
|  | Value | df | Asymp. Sig. (2-sided) | Exact Sig. (2-sided) | Exact Sig. (1-sided) |
| Pearson Chi-Square | 13.086^a^ | 1 | .000 |  |  |
| Continuity Correction^b^ | 10.764 | 1 | .001 |  |  |
| Likelihood Ratio | 11.520 | 1 | .001 |  |  |
| Fisher's Exact Test |  |  |  | .001 | .001 |
| Linear-by-Linear Association | 12.912 | 1 | .000 |  |  |
| N of Valid Cases | 75 |  |  |  |  |
| a. 1 cells (25.0%) have expected count less than 5. The minimum expected count is 3.63. | | | | | |
| b. Computed only for a 2x2 table | | | | | |

**ICU length of stay**

| **Tests of Normality** | | | | | | |
| --- | --- | --- | --- | --- | --- | --- |
|  | Kolmogorov-Smirnov^a^ | | | Shapiro-Wilk | | |
|  | Statistic | df | Sig. | Statistic | df | Sig. |
| ICU length of stay (days) | .200 | 75 | .000 | .822 | 75 | .000 |
| a. Lilliefors Significance Correction | | | | | | |

Patients

| **Descriptives** | | | | |
| --- | --- | --- | --- | --- |
|  | | | Statistic | Std. Error |
| ICU length of stay (days) | Mean | | 9.11 | .757 |
|  | 95% Confidence Interval for Mean | Lower Bound | 7.60 |  |
|  |  | Upper Bound | 10.61 |  |
|  | 5% Trimmed Mean | | 8.51 |  |
|  | Median | | 7.00 |  |
|  | Variance | | 42.934 |  |
|  | Std. Deviation | | 6.552 |  |
|  | Minimum | | 3 |  |
|  | Maximum | | 27 |  |
|  | Range | | 24 |  |
|  | Interquartile Range | | 7 |  |
|  | Skewness | | 1.314 | .277 |
|  | Kurtosis | | .800 | .548 |

| **Statistics** | | |
| --- | --- | --- |
| ICU length of stay (days) | | |
| N | Valid | 75 |
|  | Missing | 0 |
| Percentiles | 25 | 4.00 |
|  | 50 | 7.00 |
|  | 75 | 11.00 |

Oliguria

| **Descriptives** | | | | |
| --- | --- | --- | --- | --- |
|  | | | Statistic | Std. Error |
| ICU length of stay (days) | Mean | | 9.88 | 1.599 |
|  | 95% Confidence Interval for Mean | Lower Bound | 6.49 |  |
|  |  | Upper Bound | 13.27 |  |
|  | 5% Trimmed Mean | | 9.54 |  |
|  | Median | | 9.00 |  |
|  | Variance | | 43.485 |  |
|  | Std. Deviation | | 6.594 |  |
|  | Minimum | | 3 |  |
|  | Maximum | | 23 |  |
|  | Range | | 20 |  |
|  | Interquartile Range | | 11 |  |
|  | Skewness | | .775 | .550 |
|  | Kurtosis | | -.428 | 1.063 |

| **Statistics** | | |
| --- | --- | --- |
| ICU length of stay (days) | | |
| N | Valid | 17 |
|  | Missing | 0 |
| Percentiles | 25 | 4.00 |
|  | 50 | 9.00 |
|  | 75 | 14.50 |

Non-oliguria

| **Descriptives** | | | | |
| --- | --- | --- | --- | --- |
|  | | | Statistic | Std. Error |
| ICU length of stay (days) | Mean | | 8.88 | .864 |
|  | 95% Confidence Interval for Mean | Lower Bound | 7.15 |  |
|  |  | Upper Bound | 10.61 |  |
|  | 5% Trimmed Mean | | 8.22 |  |
|  | Median | | 6.00 |  |
|  | Variance | | 43.301 |  |
|  | Std. Deviation | | 6.580 |  |
|  | Minimum | | 3 |  |
|  | Maximum | | 27 |  |
|  | Range | | 24 |  |
|  | Interquartile Range | | 6 |  |
|  | Skewness | | 1.502 | .314 |
|  | Kurtosis | | 1.358 | .618 |

| **Statistics** | | |
| --- | --- | --- |
| ICU length of stay (days) | | |
| N | Valid | 58 |
|  | Missing | 0 |
| Percentiles | 25 | 4.75 |
|  | 50 | 6.00 |
|  | 75 | 10.25 |

**Nonparametric Tests of Age, Glasgow coma scale, Mean arterial pressure, Diastolic arterial pressure, Creatinine, Arterial lactate, Partial pressure of oxygen, Arterial oxygen saturation and ICU length of stay**


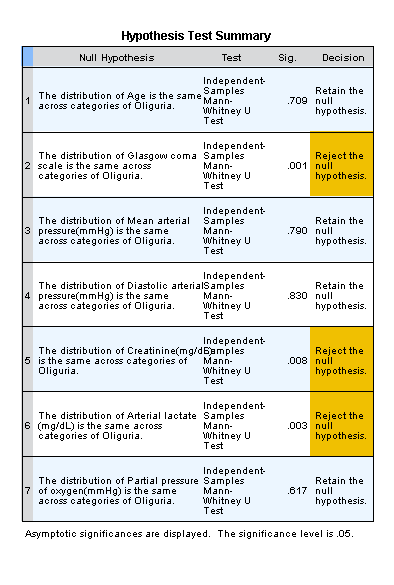


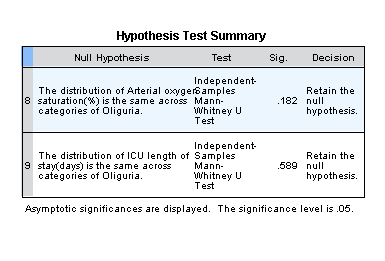


**Nonparametric Tests of VEGF**


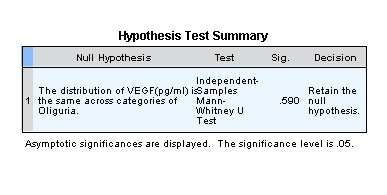


**Nonparametric Tests of Intake and Output, Urine output and Intravascular fluid administration**

**Day 1**


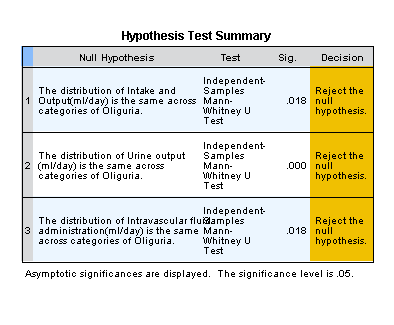


**Day 2**


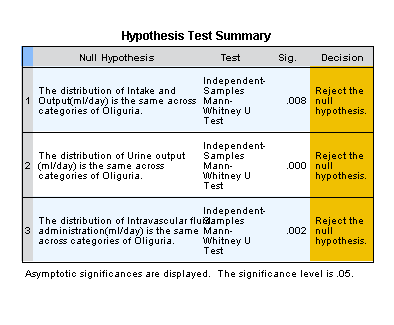


**Day 3**


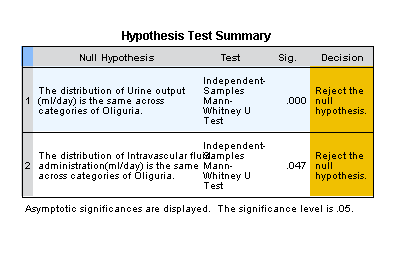


**Total average**


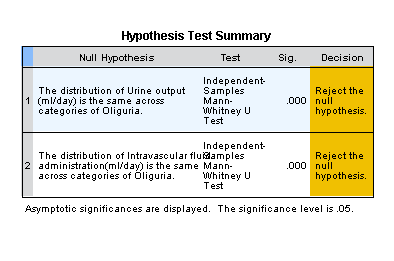


**1.2 Detailed analyses and results for Table 2**

|  |  | urine＜500 mL | | | urine≧500 mL | | |
| --- | --- | --- | --- | --- | --- | --- | --- |
|  |  | Mean | Standard Deviation | Valid N | Mean | Standard Deviation | Valid N |
| *[HbO_2_]* | Day1 | .194 | .012 | 17 | .202 | .010 | 58 |
|  | Day2 | .194 | .012 | 17 | .196 | .024 | 56 |
|  | Day3 | .175 | .032 | 11 | .196 | .023 | 51 |
| *[HbR]* | Day1 | .220 | .015 | 17 | .224 | .017 | 58 |
|  | Day2 | .219 | .019 | 17 | .231 | .035 | 56 |
|  | Day3 | .239 | .057 | 11 | .231 | .039 | 51 |
| *[HbT]* | Day1 | .414 | .020 | 17 | .425 | .022 | 58 |
|  | Day2 | .413 | .028 | 17 | .427 | .023 | 56 |
|  | Day3 | .414 | .033 | 11 | .427 | .024 | 51 |
| *StO_2_* (%) | Day1 | 46.80 | 2.06 | 17 | 47.42 | 1.96 | 58 |
|  | Day2 | 47.10 | 1.67 | 17 | 46.15 | 6.00 | 56 |
|  | Day3 | 42.77 | 9.15 | 11 | 46.20 | 6.25 | 51 |
| *[H_2_O]* | Day1 | 10.29 | 2.43 | 17 | 10.53 | 3.50 | 58 |
|  | Day2 | 9.87 | 3.82 | 17 | 10.73 | 3.34 | 56 |
|  | Day3 | 12.61 | 4.71 | 11 | 10.39 | 3.04 | 51 |

***[HbO_2_]***

Day 1

| **Descriptives** | | | | |
| --- | --- | --- | --- | --- |
|  | | | Statistic | Std. Error |
| *[HbO_2_]* (O) | Mean | | .19375 | .002818 |
|  | 95% Confidence Interval for Mean | Lower Bound | .18778 |  |
|  |  | Upper Bound | .19973 |  |
|  | 5% Trimmed Mean | | .19427 |  |
|  | Median | | .19526 |  |
|  | Variance | | .000 |  |
|  | Std. Deviation | | .011617 |  |
|  | Minimum | | .170 |  |
|  | Maximum | | .208 |  |
|  | Range | | .038 |  |
|  | Interquartile Range | | .019 |  |
|  | Skewness | | -.477 | .550 |
|  | Kurtosis | | -.846 | 1.063 |

| **Descriptives** | | | | |
| --- | --- | --- | --- | --- |
|  | | | Statistic | Std. Error |
| *[HbO_2_]* (N) | Mean | | .20155 | .001321 |
|  | 95% Confidence Interval for Mean | Lower Bound | .19890 |  |
|  |  | Upper Bound | .20419 |  |
|  | 5% Trimmed Mean | | .20206 |  |
|  | Median | | .20169 |  |
|  | Variance | | .000 |  |
|  | Std. Deviation | | .010060 |  |
|  | Minimum | | .159 |  |
|  | Maximum | | .220 |  |
|  | Range | | .061 |  |
|  | Interquartile Range | | .011 |  |
|  | Skewness | | -1.272 | .314 |
|  | Kurtosis | | 4.542 | .618 |

O:oliguric patients N:non-oliguric patients

Day 2

| **Descriptives** | | | | |
| --- | --- | --- | --- | --- |
|  | | | Statistic | Std. Error |
| *[HbO_2_]* (O) | Mean | | .19437 | .002833 |
|  | 95% Confidence Interval for Mean | Lower Bound | .18836 |  |
|  |  | Upper Bound | .20037 |  |
|  | 5% Trimmed Mean | | .19510 |  |
|  | Median | | .19681 |  |
|  | Variance | | .000 |  |
|  | Std. Deviation | | .011680 |  |
|  | Minimum | | .165 |  |
|  | Maximum | | .211 |  |
|  | Range | | .046 |  |
|  | Interquartile Range | | .016 |  |
|  | Skewness | | -.981 | .550 |
|  | Kurtosis | | 1.279 | 1.063 |

| **Descriptives** | | | | |
| --- | --- | --- | --- | --- |
|  | | | Statistic | Std. Error |
| *[HbO_2_]* (N) | Mean | | .19648 | .003171 |
|  | 95% Confidence Interval for Mean | Lower Bound | .19012 |  |
|  |  | Upper Bound | .20283 |  |
|  | 5% Trimmed Mean | | .19952 |  |
|  | Median | | .20064 |  |
|  | Variance | | .001 |  |
|  | Std. Deviation | | .023726 |  |
|  | Minimum | | .109 |  |
|  | Maximum | | .228 |  |
|  | Range | | .118 |  |
|  | Interquartile Range | | .012 |  |
|  | Skewness | | -2.677 | .319 |
|  | Kurtosis | | 7.172 | .628 |

O:oliguric patients N:non-oliguric patients

Day 3

| **Descriptives** | | | | |
| --- | --- | --- | --- | --- |
|  | | | Statistic | Std. Error |
| *[HbO_2_]* (O) | Mean | | .17483 | .009574 |
|  | 95% Confidence Interval for Mean | Lower Bound | .15349 |  |
|  |  | Upper Bound | .19616 |  |
|  | 5% Trimmed Mean | | .17743 |  |
|  | Median | | .18704 |  |
|  | Variance | | .001 |  |
|  | Std. Deviation | | .031754 |  |
|  | Minimum | | .100 |  |
|  | Maximum | | .203 |  |
|  | Range | | .103 |  |
|  | Interquartile Range | | .030 |  |
|  | Skewness | | -1.661 | .661 |
|  | Kurtosis | | 2.302 | 1.279 |

| **Descriptives** | | | | |
| --- | --- | --- | --- | --- |
|  | | | Statistic | Std. Error |
| *[HbO_2_]* (N) | Mean | | .19639 | .003187 |
|  | 95% Confidence Interval for Mean | Lower Bound | .18998 |  |
|  |  | Upper Bound | .20279 |  |
|  | 5% Trimmed Mean | | .19966 |  |
|  | Median | | .20167 |  |
|  | Variance | | .001 |  |
|  | Std. Deviation | | .022759 |  |
|  | Minimum | | .103 |  |
|  | Maximum | | .218 |  |
|  | Range | | .115 |  |
|  | Interquartile Range | | .011 |  |
|  | Skewness | | -2.813 | .333 |
|  | Kurtosis | | 8.117 | .656 |

O:oliguric patients N:non-oliguric patients

***[HbR]***

Day 1

| **Descriptives** | | | | |
| --- | --- | --- | --- | --- |
|  | | | Statistic | Std. Error |
| *[HbR]* (O) | Mean | | .22036 | .003633 |
|  | 95% Confidence Interval for Mean | Lower Bound | .21265 |  |
|  |  | Upper Bound | .22806 |  |
|  | 5% Trimmed Mean | | .21975 |  |
|  | Median | | .21924 |  |
|  | Variance | | .000 |  |
|  | Std. Deviation | | .014980 |  |
|  | Minimum | | .200 |  |
|  | Maximum | | .251 |  |
|  | Range | | .051 |  |
|  | Interquartile Range | | .019 |  |
|  | Skewness | | .711 | .550 |
|  | Kurtosis | | .122 | 1.063 |

| **Descriptives** | | | | |
| --- | --- | --- | --- | --- |
|  | | | Statistic | Std. Error |
| *[HbR]* (N) | Mean | | .22390 | .002229 |
|  | 95% Confidence Interval for Mean | Lower Bound | .21944 |  |
|  |  | Upper Bound | .22836 |  |
|  | 5% Trimmed Mean | | .22391 |  |
|  | Median | | .22229 |  |
|  | Variance | | .000 |  |
|  | Std. Deviation | | .016974 |  |
|  | Minimum | | .191 |  |
|  | Maximum | | .260 |  |
|  | Range | | .069 |  |
|  | Interquartile Range | | .027 |  |
|  | Skewness | | .046 | .314 |
|  | Kurtosis | | -.876 | .618 |

O:oliguric patients N:non-oliguric patients

Day 2

| **Descriptives** | | | | |
| --- | --- | --- | --- | --- |
|  | | | Statistic | Std. Error |
| *[HbR]* (O) | Mean | | .21869 | .004559 |
|  | 95% Confidence Interval for Mean | Lower Bound | .20903 |  |
|  |  | Upper Bound | .22836 |  |
|  | 5% Trimmed Mean | | .21779 |  |
|  | Median | | .21409 |  |
|  | Variance | | .000 |  |
|  | Std. Deviation | | .018799 |  |
|  | Minimum | | .195 |  |
|  | Maximum | | .258 |  |
|  | Range | | .063 |  |
|  | Interquartile Range | | .025 |  |
|  | Skewness | | .888 | .550 |
|  | Kurtosis | | .245 | 1.063 |

| **Descriptives** | | | | |
| --- | --- | --- | --- | --- |
|  | | | Statistic | Std. Error |
| *[HbR]* (N) | Mean | | .23075 | .004686 |
|  | 95% Confidence Interval for Mean | Lower Bound | .22136 |  |
|  |  | Upper Bound | .24014 |  |
|  | 5% Trimmed Mean | | .22607 |  |
|  | Median | | .22234 |  |
|  | Variance | | .001 |  |
|  | Std. Deviation | | .035068 |  |
|  | Minimum | | .192 |  |
|  | Maximum | | .358 |  |
|  | Range | | .165 |  |
|  | Interquartile Range | | .029 |  |
|  | Skewness | | 2.354 | .319 |
|  | Kurtosis | | 6.028 | .628 |

O:oliguric patients N:non-oliguric patients

Day 3

| **Descriptives** | | | | |
| --- | --- | --- | --- | --- |
|  | | | Statistic | Std. Error |
| *[HbR]* (O) | Mean | | .23885 | .017250 |
|  | 95% Confidence Interval for Mean | Lower Bound | .20042 |  |
|  |  | Upper Bound | .27729 |  |
|  | 5% Trimmed Mean | | .23480 |  |
|  | Median | | .21370 |  |
|  | Variance | | .003 |  |
|  | Std. Deviation | | .057210 |  |
|  | Minimum | | .194 |  |
|  | Maximum | | .357 |  |
|  | Range | | .164 |  |
|  | Interquartile Range | | .025 |  |
|  | Skewness | | 1.763 | .661 |
|  | Kurtosis | | 1.712 | 1.279 |

| **Descriptives** | | | | |
| --- | --- | --- | --- | --- |
|  | | | Statistic | Std. Error |
| *[HbR]* (N) | Mean | | .23097 | .005460 |
|  | 95% Confidence Interval for Mean | Lower Bound | .22001 |  |
|  |  | Upper Bound | .24194 |  |
|  | 5% Trimmed Mean | | .22625 |  |
|  | Median | | .21765 |  |
|  | Variance | | .002 |  |
|  | Std. Deviation | | .038992 |  |
|  | Minimum | | .192 |  |
|  | Maximum | | .357 |  |
|  | Range | | .165 |  |
|  | Interquartile Range | | .032 |  |
|  | Skewness | | 2.218 | .333 |
|  | Kurtosis | | 4.764 | .656 |

O:oliguric patients N:non-oliguric patients

***[HbT]***

Day 1

| **Descriptives** | | | | |
| --- | --- | --- | --- | --- |
|  | | | Statistic | Std. Error |
| *[HbT]* (O) | Mean | | .41411 | .004967 |
|  | 95% Confidence Interval for Mean | Lower Bound | .40358 |  |
|  |  | Upper Bound | .42464 |  |
|  | 5% Trimmed Mean | | .41374 |  |
|  | Median | | .41128 |  |
|  | Variance | | .000 |  |
|  | Std. Deviation | | .020480 |  |
|  | Minimum | | .379 |  |
|  | Maximum | | .456 |  |
|  | Range | | .078 |  |
|  | Interquartile Range | | .019 |  |
|  | Skewness | | .627 | .550 |
|  | Kurtosis | | .455 | 1.063 |

| **Descriptives** | | | | |
| --- | --- | --- | --- | --- |
|  | | | Statistic | Std. Error |
| *[HbT]* (N) | Mean | | .42545 | .002889 |
|  | 95% Confidence Interval for Mean | Lower Bound | .41967 |  |
|  |  | Upper Bound | .43123 |  |
|  | 5% Trimmed Mean | | .42640 |  |
|  | Median | | .42334 |  |
|  | Variance | | .000 |  |
|  | Std. Deviation | | .021999 |  |
|  | Minimum | | .355 |  |
|  | Maximum | | .462 |  |
|  | Range | | .107 |  |
|  | Interquartile Range | | .032 |  |
|  | Skewness | | -.498 | .314 |
|  | Kurtosis | | .530 | .618 |

O:oliguric patients N:non-oliguric patients

Day 2

| **Descriptives** | | | | |
| --- | --- | --- | --- | --- |
|  | | | Statistic | Std. Error |
| *[HbT]* (O) | Mean | | .41306 | .006696 |
|  | 95% Confidence Interval for Mean | Lower Bound | .39887 |  |
|  |  | Upper Bound | .42726 |  |
|  | 5% Trimmed Mean | | .41289 |  |
|  | Median | | .40845 |  |
|  | Variance | | .001 |  |
|  | Std. Deviation | | .027608 |  |
|  | Minimum | | .360 |  |
|  | Maximum | | .469 |  |
|  | Range | | .109 |  |
|  | Interquartile Range | | .034 |  |
|  | Skewness | | .423 | .550 |
|  | Kurtosis | | .411 | 1.063 |

| **Descriptives** | | | | |
| --- | --- | --- | --- | --- |
|  | | | Statistic | Std. Error |
| *[HbT]* (N) | Mean | | .42723 | .003052 |
|  | 95% Confidence Interval for Mean | Lower Bound | .42111 |  |
|  |  | Upper Bound | .43334 |  |
|  | 5% Trimmed Mean | | .42710 |  |
|  | Median | | .42461 |  |
|  | Variance | | .001 |  |
|  | Std. Deviation | | .022840 |  |
|  | Minimum | | .383 |  |
|  | Maximum | | .477 |  |
|  | Range | | .094 |  |
|  | Interquartile Range | | .032 |  |
|  | Skewness | | .129 | .319 |
|  | Kurtosis | | -.543 | .628 |

O:oliguric patients N:non-oliguric patients

Day 3

| **Descriptives** | | | | |
| --- | --- | --- | --- | --- |
|  | | | Statistic | Std. Error |
| *[HbT]* (O) | Mean | | .41368 | .009881 |
|  | 95% Confidence Interval for Mean | Lower Bound | .39166 |  |
|  |  | Upper Bound | .43569 |  |
|  | 5% Trimmed Mean | | .41296 |  |
|  | Median | | .40542 |  |
|  | Variance | | .001 |  |
|  | Std. Deviation | | .032772 |  |
|  | Minimum | | .359 |  |
|  | Maximum | | .482 |  |
|  | Range | | .123 |  |
|  | Interquartile Range | | .028 |  |
|  | Skewness | | .743 | .661 |
|  | Kurtosis | | 1.266 | 1.279 |

| **Descriptives** | | | | |
| --- | --- | --- | --- | --- |
|  | | | Statistic | Std. Error |
| *[HbT]* (N) | Mean | | .42736 | .003395 |
|  | 95% Confidence Interval for Mean | Lower Bound | .42054 |  |
|  |  | Upper Bound | .43418 |  |
|  | 5% Trimmed Mean | | .42667 |  |
|  | Median | | .42298 |  |
|  | Variance | | .001 |  |
|  | Std. Deviation | | .024244 |  |
|  | Minimum | | .385 |  |
|  | Maximum | | .485 |  |
|  | Range | | .100 |  |
|  | Interquartile Range | | .037 |  |
|  | Skewness | | .538 | .333 |
|  | Kurtosis | | -.403 | .656 |

O:oliguric patients N:non-oliguric patients

***StO_2_***

Day 1

| **Descriptives** | | | | |
| --- | --- | --- | --- | --- |
|  | | | Statistic | Std. Error |
| *StO_2_* (O) | Mean | | 46.8034 | .49944 |
|  | 95% Confidence Interval for Mean | Lower Bound | 45.7447 |  |
|  |  | Upper Bound | 47.8622 |  |
|  | 5% Trimmed Mean | | 46.8578 |  |
|  | Median | | 47.3630 |  |
|  | Variance | | 4.240 |  |
|  | Std. Deviation | | 2.05922 |  |
|  | Minimum | | 42.15 |  |
|  | Maximum | | 50.48 |  |
|  | Range | | 8.33 |  |
|  | Interquartile Range | | 3.28 |  |
|  | Skewness | | -.555 | .550 |
|  | Kurtosis | | .279 | 1.063 |

| **Descriptives** | | | | |
| --- | --- | --- | --- | --- |
|  | | | Statistic | Std. Error |
| *StO_2_* (N) | Mean | | 47.4156 | .25743 |
|  | 95% Confidence Interval for Mean | Lower Bound | 46.9001 |  |
|  |  | Upper Bound | 47.9311 |  |
|  | 5% Trimmed Mean | | 47.4480 |  |
|  | Median | | 47.5822 |  |
|  | Variance | | 3.844 |  |
|  | Std. Deviation | | 1.96055 |  |
|  | Minimum | | 43.18 |  |
|  | Maximum | | 50.81 |  |
|  | Range | | 7.64 |  |
|  | Interquartile Range | | 3.13 |  |
|  | Skewness | | -.164 | .314 |
|  | Kurtosis | | -.842 | .618 |

O:oliguric patients N:non-oliguric patients

Day 2

| **Descriptives** | | | | |
| --- | --- | --- | --- | --- |
|  | | | Statistic | Std. Error |
| *StO_2_* (O) | Mean | | 47.0986 | .40495 |
|  | 95% Confidence Interval for Mean | Lower Bound | 46.2401 |  |
|  |  | Upper Bound | 47.9570 |  |
|  | 5% Trimmed Mean | | 47.1193 |  |
|  | Median | | 47.6065 |  |
|  | Variance | | 2.788 |  |
|  | Std. Deviation | | 1.66967 |  |
|  | Minimum | | 44.23 |  |
|  | Maximum | | 49.59 |  |
|  | Range | | 5.36 |  |
|  | Interquartile Range | | 3.10 |  |
|  | Skewness | | -.315 | .550 |
|  | Kurtosis | | -1.061 | 1.063 |

| **Descriptives** | | | | |
| --- | --- | --- | --- | --- |
|  | | | Statistic | Std. Error |
| *StO_2_* (N) | Mean | | 46.1506 | .80197 |
|  | 95% Confidence Interval for Mean | Lower Bound | 44.5434 |  |
|  |  | Upper Bound | 47.7578 |  |
|  | 5% Trimmed Mean | | 47.0802 |  |
|  | Median | | 47.7283 |  |
|  | Variance | | 36.017 |  |
|  | Std. Deviation | | 6.00140 |  |
|  | Minimum | | 24.00 |  |
|  | Maximum | | 51.28 |  |
|  | Range | | 27.27 |  |
|  | Interquartile Range | | 3.14 |  |
|  | Skewness | | -2.948 | .319 |
|  | Kurtosis | | 8.155 | .628 |

O:oliguric patients N:non-oliguric patients

Day 3

| **Descriptives** | | | | |
| --- | --- | --- | --- | --- |
|  | | | Statistic | Std. Error |
| *StO_2_* (O) | Mean | | 42.7721 | 2.75968 |
|  | 95% Confidence Interval for Mean | Lower Bound | 36.6232 |  |
|  |  | Upper Bound | 48.9211 |  |
|  | 5% Trimmed Mean | | 43.5750 |  |
|  | Median | | 46.7107 |  |
|  | Variance | | 83.774 |  |
|  | Std. Deviation | | 9.15283 |  |
|  | Minimum | | 21.83 |  |
|  | Maximum | | 49.26 |  |
|  | Range | | 27.43 |  |
|  | Interquartile Range | | 5.73 |  |
|  | Skewness | | -1.849 | .661 |
|  | Kurtosis | | 2.284 | 1.279 |

| **Descriptives** | | | | |
| --- | --- | --- | --- | --- |
|  | | | Statistic | Std. Error |
| *StO_2_* (N) | Mean | | 46.1951 | .87575 |
|  | 95% Confidence Interval for Mean | Lower Bound | 44.4361 |  |
|  |  | Upper Bound | 47.9541 |  |
|  | 5% Trimmed Mean | | 47.1181 |  |
|  | Median | | 47.8809 |  |
|  | Variance | | 39.114 |  |
|  | Std. Deviation | | 6.25412 |  |
|  | Minimum | | 22.53 |  |
|  | Maximum | | 51.68 |  |
|  | Range | | 29.15 |  |
|  | Interquartile Range | | 3.63 |  |
|  | Skewness | | -2.739 | .333 |
|  | Kurtosis | | 7.154 | .656 |

O:oliguric patients N:non-oliguric patients

***[H_2_O]***

Day 1

| **Descriptives** | | | | |
| --- | --- | --- | --- | --- |
|  | | | Statistic | Std. Error |
| *[H_2_O]* (O) | Mean | | 10.2896 | .58956 |
|  | 95% Confidence Interval for Mean | Lower Bound | 9.0397 |  |
|  |  | Upper Bound | 11.5394 |  |
|  | 5% Trimmed Mean | | 10.2428 |  |
|  | Median | | 9.9132 |  |
|  | Variance | | 5.909 |  |
|  | Std. Deviation | | 2.43082 |  |
|  | Minimum | | 6.31 |  |
|  | Maximum | | 15.11 |  |
|  | Range | | 8.80 |  |
|  | Interquartile Range | | 4.05 |  |
|  | Skewness | | .233 | .550 |
|  | Kurtosis | | -.721 | 1.063 |

| **Descriptives** | | | | |
| --- | --- | --- | --- | --- |
|  | | | Statistic | Std. Error |
| *[H_2_O]* (N) | Mean | | 10.5253 | .46012 |
|  | 95% Confidence Interval for Mean | Lower Bound | 9.6039 |  |
|  |  | Upper Bound | 11.4467 |  |
|  | 5% Trimmed Mean | | 10.2811 |  |
|  | Median | | 9.8967 |  |
|  | Variance | | 12.279 |  |
|  | Std. Deviation | | 3.50420 |  |
|  | Minimum | | 5.66 |  |
|  | Maximum | | 23.05 |  |
|  | Range | | 17.40 |  |
|  | Interquartile Range | | 4.06 |  |
|  | Skewness | | 1.200 | .314 |
|  | Kurtosis | | 1.872 | .618 |

O:oliguric patients N:non-oliguric patients

Day 2

| **Descriptives** | | | | |
| --- | --- | --- | --- | --- |
|  | | | Statistic | Std. Error |
| *[H_2_O]* (O) | Mean | | 9.8706 | .92734 |
|  | 95% Confidence Interval for Mean | Lower Bound | 7.9047 |  |
|  |  | Upper Bound | 11.8365 |  |
|  | 5% Trimmed Mean | | 9.5444 |  |
|  | Median | | 9.1842 |  |
|  | Variance | | 14.619 |  |
|  | Std. Deviation | | 3.82354 |  |
|  | Minimum | | 4.58 |  |
|  | Maximum | | 21.03 |  |
|  | Range | | 16.45 |  |
|  | Interquartile Range | | 3.04 |  |
|  | Skewness | | 1.611 | .550 |
|  | Kurtosis | | 3.752 | 1.063 |

| **Descriptives** | | | | |
| --- | --- | --- | --- | --- |
|  | | | Statistic | Std. Error |
| *[H_2_O]* (N) | Mean | | 10.7256 | .44650 |
|  | 95% Confidence Interval for Mean | Lower Bound | 9.8308 |  |
|  |  | Upper Bound | 11.6205 |  |
|  | 5% Trimmed Mean | | 10.6132 |  |
|  | Median | | 10.6844 |  |
|  | Variance | | 11.164 |  |
|  | Std. Deviation | | 3.34133 |  |
|  | Minimum | | 4.77 |  |
|  | Maximum | | 19.84 |  |
|  | Range | | 15.07 |  |
|  | Interquartile Range | | 5.01 |  |
|  | Skewness | | .460 | .319 |
|  | Kurtosis | | -.423 | .628 |

O:oliguric patients N:non-oliguric patients

Day 3

| **Descriptives** | | | | |
| --- | --- | --- | --- | --- |
|  | | | Statistic | Std. Error |
| *[H_2_O]* (O) | Mean | | 12.6112 | 1.42087 |
|  | 95% Confidence Interval for Mean | Lower Bound | 9.4453 |  |
|  |  | Upper Bound | 15.7771 |  |
|  | 5% Trimmed Mean | | 12.5268 |  |
|  | Median | | 11.1564 |  |
|  | Variance | | 22.207 |  |
|  | Std. Deviation | | 4.71248 |  |
|  | Minimum | | 6.43 |  |
|  | Maximum | | 20.31 |  |
|  | Range | | 13.88 |  |
|  | Interquartile Range | | 8.01 |  |
|  | Skewness | | .303 | .661 |
|  | Kurtosis | | -1.377 | 1.279 |

| **Descriptives** | | | | |
| --- | --- | --- | --- | --- |
|  | | | Statistic | Std. Error |
| *[H_2_O]* (N) | Mean | | 10.3889 | .42501 |
|  | 95% Confidence Interval for Mean | Lower Bound | 9.5352 |  |
|  |  | Upper Bound | 11.2425 |  |
|  | 5% Trimmed Mean | | 10.3199 |  |
|  | Median | | 10.3481 |  |
|  | Variance | | 9.212 |  |
|  | Std. Deviation | | 3.03517 |  |
|  | Minimum | | 4.60 |  |
|  | Maximum | | 17.96 |  |
|  | Range | | 13.36 |  |
|  | Interquartile Range | | 3.79 |  |
|  | Skewness | | .284 | .333 |
|  | Kurtosis | | -.147 | .656 |

O:oliguric patients N:non-oliguric patients

**1.3 Detailed analyses and results for Table 3**

**Tests of Normality**

***[H_2_O]***

**Day 1**

| **Tests of Normality** | | | | | | |
| --- | --- | --- | --- | --- | --- | --- |
|  | Kolmogorov-Smirnov^a^ | | | Shapiro-Wilk | | |
|  | Statistic | df | Sig. | Statistic | df | Sig. |
| *[H_2_O]* | .092 | 75 | .184 | .929 | 75 | .000 |
| a. Lilliefors Significance Correction | | | | | | |

**Day 2**

| **Tests of Normality** | | | | | | |
| --- | --- | --- | --- | --- | --- | --- |
|  | Kolmogorov-Smirnov^a^ | | | Shapiro-Wilk | | |
|  | Statistic | df | Sig. | Statistic | df | Sig. |
| *[H_2_O]* | .124 | 73 | .007 | .956 | 73 | .013 |
| a. Lilliefors Significance Correction | | | | | | |

**Day 3**

| **Tests of Normality** | | | | | | |
| --- | --- | --- | --- | --- | --- | --- |
|  | Kolmogorov-Smirnov^a^ | | | Shapiro-Wilk | | |
|  | Statistic | df | Sig. | Statistic | df | Sig. |
| *[H_2_O]* | .086 | 62 | .200^*^ | .973 | 62 | .186 |
| *. This is a lower bound of the true significance. | | | | | | |
| a. Lilliefors Significance Correction | | | | | | |

***[HbO_2_]***

**Day 1**

| **Tests of Normality** | | | | | | |
| --- | --- | --- | --- | --- | --- | --- |
|  | Kolmogorov-Smirnov^a^ | | | Shapiro-Wilk | | |
|  | Statistic | df | Sig. | Statistic | df | Sig. |
| *[HbO_2_]* | .123 | 75 | .007 | .947 | 75 | .003 |
| a. Lilliefors Significance Correction | | | | | | |

**Day 2**

| **Tests of Normality** | | | | | | |
| --- | --- | --- | --- | --- | --- | --- |
|  | Kolmogorov-Smirnov^a^ | | | Shapiro-Wilk | | |
|  | Statistic | df | Sig. | Statistic | df | Sig. |
| *[HbO_2_]* | .242 | 73 | .000 | .679 | 73 | .000 |
| a. Lilliefors Significance Correction | | | | | | |

**Day 3**

| **Tests of Normality** | | | | | | |
| --- | --- | --- | --- | --- | --- | --- |
|  | Kolmogorov-Smirnov^a^ | | | Shapiro-Wilk | | |
|  | Statistic | df | Sig. | Statistic | df | Sig. |
| *[HbO_2_]* | .282 | 62 | .000 | .690 | 62 | .000 |
| a. Lilliefors Significance Correction | | | | | | |

***[HbR]***

**Day 1**

| **Tests of Normality** | | | | | | |
| --- | --- | --- | --- | --- | --- | --- |
|  | Kolmogorov-Smirnov^a^ | | | Shapiro-Wilk | | |
|  | Statistic | df | Sig. | Statistic | df | Sig. |
| *[HbR]* | .093 | 75 | .178 | .977 | 75 | .194 |
| a. Lilliefors Significance Correction | | | | | | |

**Day 2**

| **Tests of Normality** | | | | | | |
| --- | --- | --- | --- | --- | --- | --- |
|  | Kolmogorov-Smirnov^a^ | | | Shapiro-Wilk | | |
|  | Statistic | df | Sig. | Statistic | df | Sig. |
| *[HbR]* | .175 | 73 | .000 | .741 | 73 | .000 |
| a. Lilliefors Significance Correction | | | | | | |

**Day 3**

| **Tests of Normality** | | | | | | |
| --- | --- | --- | --- | --- | --- | --- |
|  | Kolmogorov-Smirnov^a^ | | | Shapiro-Wilk | | |
|  | Statistic | df | Sig. | Statistic | df | Sig. |
| *[HbR]* | .227 | 62 | .000 | .702 | 62 | .000 |
| a. Lilliefors Significance Correction | | | | | | |

***[HbT]***

**Day 1**

| **Tests of Normality** | | | | | | |
| --- | --- | --- | --- | --- | --- | --- |
|  | Kolmogorov-Smirnov^a^ | | | Shapiro-Wilk | | |
|  | Statistic | df | Sig. | Statistic | df | Sig. |
| *[HbT]* | .071 | 75 | .200^*^ | .970 | 75 | .072 |
| *. This is a lower bound of the true significance. | | | | | | |
| a. Lilliefors Significance Correction | | | | | | |

**Day 2**

| **Tests of Normality** | | | | | | |
| --- | --- | --- | --- | --- | --- | --- |
|  | Kolmogorov-Smirnov^a^ | | | Shapiro-Wilk | | |
|  | Statistic | df | Sig. | Statistic | df | Sig. |
| *[HbT]* | .070 | 73 | .200^*^ | .987 | 73 | .671 |
| *. This is a lower bound of the true significance. | | | | | | |
| a. Lilliefors Significance Correction | | | | | | |

**Day 3**

| **Tests of Normality** | | | | | | |
| --- | --- | --- | --- | --- | --- | --- |
|  | Kolmogorov-Smirnov^a^ | | | Shapiro-Wilk | | |
|  | Statistic | df | Sig. | Statistic | df | Sig. |
| *[HbT]* | .132 | 62 | .009 | .960 | 62 | .043 |
| a. Lilliefors Significance Correction | | | | | | |

***StO_2_***

**Day 1**

| **Tests of Normality** | | | | | | |
| --- | --- | --- | --- | --- | --- | --- |
|  | Kolmogorov-Smirnov^a^ | | | Shapiro-Wilk | | |
|  | Statistic | df | Sig. | Statistic | df | Sig. |
| *StO_2_* (%) | .075 | 75 | .200^*^ | .981 | 75 | .324 |
| *. This is a lower bound of the true significance. | | | | | | |
| a. Lilliefors Significance Correction | | | | | | |

**Day 2**

| **Tests of Normality** | | | | | | |
| --- | --- | --- | --- | --- | --- | --- |
|  | Kolmogorov-Smirnov^a^ | | | Shapiro-Wilk | | |
|  | Statistic | df | Sig. | Statistic | df | Sig. |
| *StO_2_* (%) | .281 | 73 | .000 | .558 | 73 | .000 |
| a. Lilliefors Significance Correction | | | | | | |

**Day 3**

| **Tests of Normality** | | | | | | |
| --- | --- | --- | --- | --- | --- | --- |
|  | Kolmogorov-Smirnov^a^ | | | Shapiro-Wilk | | |
|  | Statistic | df | Sig. | Statistic | df | Sig. |
| *StO_2_* (%) | .284 | 62 | .000 | .628 | 62 | .000 |
| a. Lilliefors Significance Correction | | | | | | |

**Intake and Output (Data recorded on the day prior to the NIRS measurement)**

**Day 1**

| **Tests of Normality** | | | | | | |
| --- | --- | --- | --- | --- | --- | --- |
|  | Kolmogorov-Smirnov^a^ | | | Shapiro-Wilk | | |
|  | Statistic | df | Sig. | Statistic | df | Sig. |
| Intake and Output (mL/day) | .108 | 68 | .048 | .971 | 68 | .109 |
| a. Lilliefors Significance Correction | | | | | | |

**Day 2**

| **Tests of Normality** | | | | | | |
| --- | --- | --- | --- | --- | --- | --- |
|  | Kolmogorov-Smirnov^a^ | | | Shapiro-Wilk | | |
|  | Statistic | df | Sig. | Statistic | df | Sig. |
| Intake and Output (mL/day) | .115 | 73 | .017 | .947 | 73 | .004 |
| a. Lilliefors Significance Correction | | | | | | |

**Day 3**

| **Tests of Normality** | | | | | | |
| --- | --- | --- | --- | --- | --- | --- |
|  | Kolmogorov-Smirnov^a^ | | | Shapiro-Wilk | | |
|  | Statistic | df | Sig. | Statistic | df | Sig. |
| Intake and Output (mL/day) | .081 | 62 | .200^*^ | .982 | 62 | .513 |
| *. This is a lower bound of the true significance. | | | | | | |
| a. Lilliefors Significance Correction | | | | | | |

**Urine output (Data recorded on the day prior to the NIRS measurement)**

**Day 1**

| **Tests of Normality** | | | | | | |
| --- | --- | --- | --- | --- | --- | --- |
|  | Kolmogorov-Smirnov^a^ | | | Shapiro-Wilk | | |
|  | Statistic | df | Sig. | Statistic | df | Sig. |
| Urine output (mL/day) | .164 | 68 | .000 | .784 | 68 | .000 |
| a. Lilliefors Significance Correction | | | | | | |

**Day 2**

| **Tests of Normality** | | | | | | |
| --- | --- | --- | --- | --- | --- | --- |
|  | Kolmogorov-Smirnov^a^ | | | Shapiro-Wilk | | |
|  | Statistic | df | Sig. | Statistic | df | Sig. |
| Urine output (mL/day) | .110 | 73 | .029 | .919 | 73 | .000 |
| a. Lilliefors Significance Correction | | | | | | |

**Day 3**

| **Tests of Normality** | | | | | | |
| --- | --- | --- | --- | --- | --- | --- |
|  | Kolmogorov-Smirnov^a^ | | | Shapiro-Wilk | | |
|  | Statistic | df | Sig. | Statistic | df | Sig. |
| Urine output (mL/day) | .134 | 62 | .007 | .932 | 62 | .002 |
| a. Lilliefors Significance Correction | | | | | | |

**Intravascular fluid administration (Data recorded on the day prior to the NIRS measurement)**

**Day 1**

| **Tests of Normality** | | | | | | |
| --- | --- | --- | --- | --- | --- | --- |
|  | Kolmogorov-Smirnov^a^ | | | Shapiro-Wilk | | |
|  | Statistic | df | Sig. | Statistic | df | Sig. |
| Intravascular fluid administration (mL/day) | .131 | 68 | .006 | .866 | 68 | .000 |
| a. Lilliefors Significance Correction | | | | | | |

**Day 2**

| **Tests of Normality** | | | | | | |
| --- | --- | --- | --- | --- | --- | --- |
|  | Kolmogorov-Smirnov^a^ | | | Shapiro-Wilk | | |
|  | Statistic | df | Sig. | Statistic | df | Sig. |
| Intravascular fluid administration (mL/day) | .154 | 73 | .000 | .872 | 73 | .000 |
| a. Lilliefors Significance Correction | | | | | | |

**Day 3**

| **Tests of Normality** | | | | | | |
| --- | --- | --- | --- | --- | --- | --- |
|  | Kolmogorov-Smirnov^a^ | | | Shapiro-Wilk | | |
|  | Statistic | df | Sig. | Statistic | df | Sig. |
| Intravascular fluid administration (mL/day) | .153 | 62 | .001 | .893 | 62 | .000 |
| a. Lilliefors Significance Correction | | | | | | |

**VEGF**

**Day 1**

| **Tests of Normality** | | | | | | |
| --- | --- | --- | --- | --- | --- | --- |
|  | Kolmogorov-Smirnov^a^ | | | Shapiro-Wilk | | |
|  | Statistic | df | Sig. | Statistic | df | Sig. |
| VEGF (pg/mL) | .172 | 69 | .000 | .825 | 69 | .000 |
| a. Lilliefors Significance Correction | | | | | | |

**Albumin**

**Day 1**

| **Tests of Normality** | | | | | | |
| --- | --- | --- | --- | --- | --- | --- |
|  | Kolmogorov-Smirnov^a^ | | | Shapiro-Wilk | | |
|  | Statistic | df | Sig. | Statistic | df | Sig. |
| Albumin (g/dL) | .083 | 66 | .200^*^ | .990 | 66 | .891 |
| *. This is a lower bound of the true significance. | | | | | | |
| a. Lilliefors Significance Correction | | | | | | |

**Day 1**

**Pearson’s correlation coefficients**

| **Correlations** | | | | | | |
| --- | --- | --- | --- | --- | --- | --- |
|  | | *[H2O]* | *[HbO2]* | *[HbR]* | *[HbT]* | *StO2* |
| Intake and Output | Pearson Correlation | -.075 | -.216 | -.129 | -.207 | -.041 |
|  | Sig. (2-tailed) | .544 | .077 | .295 | .091 | .741 |
|  | N | 68 | 68 | 68 | 68 | 68 |
| Urine output | Pearson Correlation | -.023 | .142 | .119 | .162 | -.004 |
|  | Sig. (2-tailed) | .854 | .249 | .333 | .188 | .973 |
|  | N | 68 | 68 | 68 | 68 | 68 |
| Intravascular fluid administration | Pearson Correlation | .015 | -.233 | -.117 | -.207 | -.067 |
|  | Sig. (2-tailed) | .901 | .056 | .341 | .091 | .585 |
|  | N | 68 | 68 | 68 | 68 | 68 |
| VEGF (pg/mL) | Pearson Correlation | .406^**^ | -.274^*^ | -.053 | -.179 | -.146 |
|  | Sig. (2-tailed) | .001 | .023 | .663 | .142 | .232 |
|  | N | 69 | 69 | 69 | 69 | 69 |
| **Albumin (g/dL)** | **Pearson Correlation** | **-.329^**^** | .118 | **-.024** | **.036** | **.085** |
|  | Sig. (2-tailed) | .007 | .345 | .848 | .773 | .495 |
|  | N | 66 | 66 | 66 | 66 | 66 |
| **. Correlation is significant at the 0.01 level (2-tailed). | | | | | | |
| *. Correlation is significant at the 0.05 level (2-tailed). | | | | | | |

**Day 2**

**Pearson’s correlation coefficients**

| **Correlations** | | | | | | |
| --- | --- | --- | --- | --- | --- | --- |
|  | | *[H2O]* | *[HbO2]* | *[HbR]* | *[HbT]* | *StO2* |
| Intake and Output | Pearson Correlation | -.090 | .081 | .058 | .148 | .004 |
|  | Sig. (2-tailed) | .447 | .494 | .624 | .212 | .974 |
|  | N | 73 | 73 | 73 | 73 | 73 |
| Urine output | Pearson Correlation | .077 | .079 | .090 | .188 | -.020 |
|  | Sig. (2-tailed) | .516 | .506 | .447 | .111 | .866 |
|  | N | 73 | 73 | 73 | 73 | 73 |
| Intravascular fluid administration | Pearson Correlation | -.003 | .101 | .017 | .111 | .035 |
|  | Sig. (2-tailed) | .980 | .398 | .884 | .351 | .769 |
|  | N | 73 | 73 | 73 | 73 | 73 |
| **. Correlation is significant at the 0.01 level (2-tailed). | | | | | | |
| *. Correlation is significant at the 0.05 level (2-tailed). | | | | | | |

**Day 3**

**Pearson’s correlation coefficients**

| **Correlations** | | | | | | |
| --- | --- | --- | --- | --- | --- | --- |
|  | | *[H2O]* | *[HbO2]* | *[HbR]* | *[HbT]* | *StO2* |
| **Intake and Output** | **Pearson Correlation** | **.046** | .028 | -.198 | -.292^*^ | .130 |
|  | Sig. (2-tailed) | .721 | .830 | .123 | .021 | .314 |
|  | N | 62 | 62 | 62 | 62 | 62 |
| Urine output | Pearson Correlation | -.262^*^ | .301^*^ | -.029 | .247 | .149 |
|  | Sig. (2-tailed) | .040 | .018 | .822 | .052 | .248 |
|  | N | 62 | 62 | 62 | 62 | 62 |
| Intravascular fluid administration | Pearson Correlation | .091 | -.022 | -.036 | -.080 | .007 |
|  | Sig. (2-tailed) | .480 | .866 | .780 | .537 | .957 |
|  | N | 62 | 62 | 62 | 62 | 62 |
| **. Correlation is significant at the 0.01 level (2-tailed). | | | | | | |
| *. Correlation is significant at the 0.05 level (2-tailed). | | | | | | |

**Day 1**

**Spearman’s rho correlation coefficients**

| Correlations | | | | | | |
| --- | --- | --- | --- | --- | --- | --- |
|  | | *[H2O]* | *[HbO2]* | *[HbR]* | *[HbT]* | *StO2* |
| **Intake and Output** | **Correlation Coefficient** | **-.050** | **-.139** | **-.142** | **-.223** | **.012** |
|  | Sig. (2-tailed) | .687 | .257 | .247 | .067 | .923 |
|  | N | 68 | 68 | 68 | 68 | 68 |
| **Urine output** | **Correlation Coefficient** | **-.084** | **.233** | **-.025** | **.136** | **.181** |
|  | Sig. (2-tailed) | .498 | .056 | .838 | .269 | .139 |
|  | N | 68 | 68 | 68 | 68 | 68 |
| **Intravascular fluid administration** | **Correlation Coefficient** | **.010** | **-.143** | **-.204** | **-.259^*^** | **.029** |
|  | Sig. (2-tailed) | .937 | .246 | .095 | .033 | .816 |
|  | N | 68 | 68 | 68 | 68 | 68 |
| **VEGF (pg/mL)** | **Correlation Coefficient** | **.449^**^** | **-.246^*^** | **-.105** | **-.266^*^** | **-.105** |
|  | Sig. (2-tailed) | .000 | .042 | .388 | .027 | .392 |
|  | N | 69 | 69 | 69 | 69 | 69 |
| **Albumin (g/dL)** | **Correlation Coefficient** | -.336^**^ | **.130** | -.010 | .044 | .075 |
|  | Sig. (2-tailed) | .006 | .300 | .936 | .726 | .549 |
|  | N | 66 | 66 | 66 | 66 | 66 |
| **. Correlation is significant at the 0.01 level (2-tailed). | | | | | | |
| *. Correlation is significant at the 0.05 level (2-tailed). | | | | | | |

**Day 2**

**Spearman’s rho correlation coefficients**

| **Correlations** | | | | | | |
| --- | --- | --- | --- | --- | --- | --- |
|  |  | *[H2O]* | *[HbO2]* | *[HbR]* | *[HbT]* | *StO2* |
| **Intake and Output** | **Correlation Coefficient** | **-.013** | **.036** | **.036** | **.041** | **.014** |
|  | Sig. (2-tailed) | .912 | .761 | .765 | .731 | .907 |
|  | N | 73 | 73 | 73 | 73 | 73 |
| **Urine output** | **Correlation Coefficient** | **.098** | **.204** | **.156** | **.194** | **.038** |
|  | Sig. (2-tailed) | .411 | .083 | .187 | .100 | .750 |
|  | N | 73 | 73 | 73 | 73 | 73 |
| **Intravascular fluid administration** | **Correlation Coefficient** | **.077** | **-.021** | **.059** | **.043** | **-.080** |
|  | Sig. (2-tailed) | .517 | .860 | .620 | .721 | .501 |
|  | N | 73 | 73 | 73 | 73 | 73 |
| **. Correlation is significant at the 0.01 level (2-tailed). | | | | | | |
| *. Correlation is significant at the 0.05 level (2-tailed). | | | | | | |

**Day 3**

**Spearman’s rho correlation coefficients**

| **Correlations** | | | | | | |
| --- | --- | --- | --- | --- | --- | --- |
|  |  | *[H2O]* | *[HbO2]* | *[HbR]* | *[HbT]* | *StO2* |
| **Intake and Output** | **Correlation Coefficient** | .101 | **-.140** | **-.166** | **-.268^*^** | **.061** |
|  | Sig. (2-tailed) | .433 | .276 | .196 | .035 | .638 |
|  | N | 62 | 62 | 62 | 62 | 62 |
| **Urine output** | **Correlation Coefficient** | **-.240** | **.367^**^** | **.136** | **.297^*^** | **.099** |
|  | Sig. (2-tailed) | .060 | .003 | .293 | .019 | .443 |
|  | N | 62 | 62 | 62 | 62 | 62 |
| **Intravascular fluid administration** | **Correlation Coefficient** | **.179** | **-.092** | **-.025** | **-.142** | **-.042** |
|  | Sig. (2-tailed) | .164 | .477 | .846 | .270 | .747 |
|  | N | 62 | 62 | 62 | 62 | 62 |
| **. Correlation is significant at the 0.01 level (2-tailed). | | | | | | |
| *. Correlation is significant at the 0.05 level (2-tailed). | | | | | | |

**1.4 Detailed analyses and results for Table 4**

**Tests of Normality**

**Intake and Output (Data recorded on the day prior to the NIRS measurement)**

**Day 1**

| **Tests of Normality** | | | | | | |
| --- | --- | --- | --- | --- | --- | --- |
|  | Kolmogorov-Smirnov^a^ | | | Shapiro-Wilk | | |
|  | Statistic | df | Sig. | Statistic | df | Sig. |
| Intake and Output (mL/day) | .108 | 68 | .048 | .971 | 68 | .109 |
| a. Lilliefors Significance Correction | | | | | | |

**Urine output (Data recorded on the day prior to the NIRS measurement)**

**Day 1**

| **Tests of Normality** | | | | | | |
| --- | --- | --- | --- | --- | --- | --- |
|  | Kolmogorov-Smirnov^a^ | | | Shapiro-Wilk | | |
|  | Statistic | df | Sig. | Statistic | df | Sig. |
| Urine output (mL/day) | .164 | 68 | .000 | .784 | 68 | .000 |
| a. Lilliefors Significance Correction | | | | | | |

**Intravascular fluid administration (Data recorded on the day prior to the NIRS measurement)**

**Day 1**

| **Tests of Normality** | | | | | | |
| --- | --- | --- | --- | --- | --- | --- |
|  | Kolmogorov-Smirnov^a^ | | | Shapiro-Wilk | | |
|  | Statistic | df | Sig. | Statistic | df | Sig. |
| Intravascular fluid administration (mL/day) | .131 | 68 | .006 | .866 | 68 | .000 |
| a. Lilliefors Significance Correction | | | | | | |

**VEGF Day 1**

| **Tests of Normality** | | | | | | |
| --- | --- | --- | --- | --- | --- | --- |
|  | Kolmogorov-Smirnov^a^ | | | Shapiro-Wilk | | |
|  | Statistic | df | Sig. | Statistic | df | Sig. |
| VEGF (pg/mL) | .172 | 69 | .000 | .825 | 69 | .000 |
| a. Lilliefors Significance Correction | | | | | | |

**Albumin Day 1**

| **Tests of Normality** | | | | | | |
| --- | --- | --- | --- | --- | --- | --- |
|  | Kolmogorov-Smirnov^a^ | | | Shapiro-Wilk | | |
|  | Statistic | df | Sig. | Statistic | df | Sig. |
| Albumin (g/dL) | .083 | 66 | .200^*^ | .990 | 66 | .891 |
| *. This is a lower bound of the true significance. | | | | | | |
| a. Lilliefors Significance Correction | | | | | | |

**Pearson’s correlation coefficients**

| **Correlations** | | | | | | |
| --- | --- | --- | --- | --- | --- | --- |
|  | | Intake and Output | Urine output | Intravascular fluid administration | VEGF | Albumin |
| Intake and Output | Pearson Correlation | 1 | -.420^**^ | .672^**^ | -.005 | .131 |
|  | Sig. (2-tailed) |  | .000 | .000 | .968 | .322 |
|  | N | 68 | 68 | 68 | 64 | 59 |
| Urine output | Pearson Correlation | -.420^**^ | 1 | -.093 | -.054 | -.143 |
|  | Sig. (2-tailed) | .000 |  | .452 | .669 | .280 |
|  | N | 68 | 68 | 68 | 64 | 59 |
| Intravascular fluid administration | Pearson Correlation | .672^**^ | -.093 | 1 | -.046 | .107 |
|  | Sig. (2-tailed) | .000 | .452 |  | .719 | .421 |
|  | N | 68 | 68 | 68 | 64 | 59 |
| VEGF (pg/mL) | Pearson Correlation | -.005 | -.054 | -.046 | 1 | -.429^**^ |
|  | Sig. (2-tailed) | .968 | .669 | .719 |  | .001 |
|  | N | 64 | 64 | 64 | 69 | 60 |
| Albumin (g/dL) | Pearson Correlation | .131 | -.143 | .107 | -.429^**^ | 1 |
|  | Sig. (2-tailed) | .322 | .280 | .421 | .001 |  |
|  | N | 59 | 59 | 59 | 60 | 66 |
| **. Correlation is significant at the 0.01 level (2-tailed). | | | | | | |

**Spearman’s rho correlation coefficients**

| **Correlations** | | | | | | |
| --- | --- | --- | --- | --- | --- | --- |
|  | | Intake and Output | Urine output | Intravascular fluid administration | VEGF | Albumin |
| **Intake and Output** | **Correlation Coefficient** | **1.000** | **-.390^**^** | **.544^**^** | **.093** | **.107** |
|  | Sig. (2-tailed) |  | .001 | .000 | .464 | .419 |
|  | N | 68 | 68 | 68 | 64 | 59 |
| **Urine output** | **Correlation Coefficient** | **-.390^**^** | **1.000** | **-.043** | **.011** | **-.143** |
|  | Sig. (2-tailed) | .001 |  | .727 | .932 | .280 |
|  | N | 68 | 68 | 68 | 64 | 59 |
| **Intravascular fluid administration** | **Correlation Coefficient** | **.544^**^** | **-.043** | **1.000** | **-.033** | **.052** |
|  | Sig. (2-tailed) | .000 | .727 |  | .799 | .696 |
|  | N | 68 | 68 | 68 | 64 | 59 |
| **VEGF (pg/mL)** | **Correlation Coefficient** | **.093** | **.011** | **-.033** | **1.000** | **-.401^**^** |
|  | Sig. (2-tailed) | .464 | .932 | .799 |  | .002 |
|  | N | 64 | 64 | 64 | 69 | 60 |
| **Albumin (g/dL)** | **Correlation Coefficient** | **.107** | **-.143** | **.052** | **-.401^**^** | **1.000** |
|  | Sig. (2-tailed) | .419 | .280 | .696 | .002 |  |
|  | N | 59 | 59 | 59 | 60 | 66 |
| **. Correlation is significant at the 0.01 level (2-tailed). | | | | | | |

**1.5 Detailed analyses and results for Figure 2**

As the relative tissue concentrations of Hb and H_2_O and tissue oxygenation were repeatedly measured at designated intervals over time, generalized estimating equations, considering the correlation within individuals, were employed to evaluate the differences in NIRS parameters between groups.

*[HbO_2_]*

| *[HbO_2_]* | | p-value | Z |
| --- | --- | --- | --- |
| group difference at | Day1 | 0.010 |  |
|  | Day2 | 0.686 | 0.404 |
|  | Day3 | 0.052 | 1.944 |
| Urine≧500 mL | Day1 vs Day2 | 0.106 | -1.250 |
|  | Day1 vs Day3 | 0.368 | -0.338 |
|  | Day2 vs Day3 | 0.492 | -0.019 |
| Urine＜500 mL | Day1 vs Day2 | 0.775 |  |
|  | Day1 vs Day3 | 0.066 |  |
|  | Day2 vs Day3 | 0.063 | 1.860 |
| Urine≧500 mL vs Urine＜500 mL | | 0.010 |  |

| **Tests of Model Effects** | | | |
| --- | --- | --- | --- |
| Source | Type III | | |
|  | Wald Chi-Square | df | Sig. |
| (Intercept) | 10197.507 | 1 | .000 |
| day | 5.623 | 2 | .060 |
| day012UO total average | 7.226 | 1 | .007 |
| day * day012UO total average | 5.315 | 2 | .070 |
| Dependent Variable: *[HbO_2_]*  Model: (Intercept), day, day012UO total average, day * day012UO total average | | | |

| **Parameter Estimates** | | | | | | | |
| --- | --- | --- | --- | --- | --- | --- | --- |
| Parameter | B | Std. Error | 95% Wald Confidence Interval | | Hypothesis Test | | |
|  |  |  | Lower | Upper | Wald Chi-Square | df | Sig. |
| (Intercept) | .194 | .0027 | .188 | .199 | 5024.174 | 1 | .000 |
| [day=3] | -.018 | .0099 | -.038 | .001 | 3.391 | 1 | .066 |
| [day=2] | .001 | .0022 | -.004 | .005 | .082 | 1 | .775 |
| [day=1] | 0^a^ | . | . | . | . | . | . |
| [day012UO total average =1] | .008 | .0030 | .002 | .014 | 6.614 | 1 | .010 |
| [day012UO total average =0] | 0^a^ | . | . | . | . | . | . |
| [day=3] * [day012UO total average n =1] | .013 | .0104 | -.007 | .034 | 1.633 | 1 | .201 |
| [day=3] * [day012UO total average =0] | 0^a^ | . | . | . | . | . | . |
| [day=2] * [day012UO total average =1] | -.006 | .0036 | -.013 | .001 | 2.639 | 1 | .104 |
| [day=2] * [day012UO total average =0] | 0^a^ | . | . | . | . | . | . |
| [day=1] * [day012UO total average =1] | 0^a^ | . | . | . | . | . | . |
| [day=1] * [day012UO total average =0] | 0^a^ | . | . | . | . | . | . |
| (Scale) | .000 |  |  |  |  |  |  |
| Dependent Variable: *[HbO_2_]*  Model: (Intercept), day, day012UO total average, day * day012UO total average | | | | | | | |
| a. Set to zero because this parameter is redundant. | | | | | | | |

◎p-value(Day2)

Z=(β_4_+β_24_)/ √se(β_4_)^2^+se(β_24_)^2^

=(0.008+(-0.006)) / √0.003^2^+0.0036^2^= 0.404

p-value=2*(1-NORMSDIST(0.404))= 0.686

◎p-value(Day3)

Z=(β_4_+β_34_) / √se(β_4_)^2^+se(β_34_)^2^

=(0.008+0.013) / √0.003^2^+0.0104^2^= 1.944

p-value=2*(1-NORMSDIST(1.944))= 0.052

◎p-value(Urine≧500 mL:Day1 vs Day2)

Z=(β_2_+β_24_) / √se(β_2_)^2^+se(β_24_)^2^

=(0.001+(-0.006)) / √0.0022^2^+0.0036^2^= -1.250

p-value=NORMSDIST(-1.250)= 0.106

◎p-value(Urine≧500 mL:Day1 vs Day3)

Z=(β_3_+β_34_) / √se(β_3_)^2^+se(β_34_)^2^

=(-0.018+0.013) / √0.0099^2^+0.0104^2^= -0.338

p-value=NORMSDIST(-0.338)= 0.368

◎p-value Urine≧500 mL:Day2 vs Day3)

Z=〔 (β_2_+β_24_)-(β_3_+β_34_) 〕/ √〔 se(β_2_)+se(β_24_) 〕^2^+〔 se(β_3_)+se(β_34_) 〕^2^

=(0.001+(-0.006))-(-0.018+0.013) / √(0.0022+0.0036)^2^+(0.0099+0.0104)^2^

= -0.019

p-value=NORMSDIST(- 0.019)= 0.492

◎p-value(Urine＜500 mL:Day2 vs Day3)

Z=(β_2_-β_3_) / √se(β_2_)^2^+se(β_3_)^2^

=0.001-(-0.018) / √0.0022^2^+0.0099^2^= 1.860

p-value=2*(1-NORMSDIST(1.860))= 0.063

*[HbR]*

| *[HbR]* | | p-value | Z |
| --- | --- | --- | --- |
| group difference at | Day1 | 0.394 |  |
|  | Day2 | 0.059 | 1.888 |
|  | Day3 | 0.311 | -0.493 |
| Urine≧500 mL | Day1 vs Day2 | 0.214 | 1.241 |
|  | Day1 vs Day3 | 0.754 | 0.313 |
|  | Day2 vs Day3 | 0.499 | -0.004 |
| Urine＜500 mL | Day1 vs Day2 | 0.519 |  |
|  | Day1 vs Day3 | 0.219 |  |
|  | Day2 vs Day3 | 1.813 | -1.321 |
| Urine≧500 mL vs Urine＜500 mL | | 0.394 |  |

| **Tests of Model Effects** | | | |
| --- | --- | --- | --- |
| Source | Type III | | |
|  | Wald Chi-Square | df | Sig. |
| (Intercept) | 3625.877 | 1 | .000 |
| day | 3.345 | 2 | .188 |
| day012UO total average | .107 | 1 | .744 |
| day * day012UO total average | 3.980 | 2 | .137 |
| Dependent Variable: *[HbR]*  Model: (Intercept), day, day012UO total average, day * day012UO total average | | | |

| **Parameter Estimates** | | | | | | | |
| --- | --- | --- | --- | --- | --- | --- | --- |
| Parameter | B | Std. Error | 95% Wald Confidence Interval | | Hypothesis Test | | |
|  |  |  | Lower | Upper | Wald Chi-Square | df | Sig. |
| (Intercept) | .220 | .0035 | .213 | .227 | 3908.242 | 1 | .000 |
| [day=3] | .018 | .0150 | -.011 | .048 | 1.511 | 1 | .219 |
| [day=2] | -.002 | .0026 | -.007 | .003 | .416 | 1 | .519 |
| [day=1] | 0^a^ | . | . | . | . | . | . |
| [day012UO total average =1] | .004 | .0042 | -.005 | .012 | .726 | 1 | .394 |
| [day012UO total average =0] | 0^a^ | . | . | . | . | . | . |
| [day=3] * [day012UO total average =1] | -.012 | .0158 | -.043 | .019 | .538 | 1 | .463 |
| [day=3] * [day012UO total average =0] | 0^a^ | . | . | . | . | . | . |
| [day=2] * [day012UO total average =1] | .008 | .0048 | -.001 | .018 | 3.108 | 1 | .078 |
| [day=2] * [day012UO total average =0] | 0^a^ | . | . | . | . | . | . |
| [day=1] * [day012UO total average =1] | 0^a^ | . | . | . | . | . | . |
| [day=1] * [day012UO total average =0] | 0^a^ | . | . | . | . | . | . |
| (Scale) | .001 |  |  |  |  |  |  |
| Dependent Variable: *[HbR]*  Model: (Intercept), day, day012UO total average, day * day012UO total average | | | | | | | |
| a. Set to zero because this parameter is redundant. | | | | | | | |

◎p-value(Day2)

Z=(β_4_+β_24_) / √se(β_4_)^2^+se(β_24_)^2^

=(0.004+0.008) / √0.0042^2^+0.0048^2^= 1.888

p-value=2*(1-NORMSDIST(1.888))= 0.059

◎p-value(Day3)

Z=(β_4_+β_34_) / √se(β_4_)^2^+se(β_34_)^2^

=(0.004+(-0.012)) / √0.0042^2^+0.0158^2^= -0.493

p-value=NORMSDIST(-0.493)= 0.311

◎p-value(Urine≧500 mL:Day1 vs Day2)

Z=(β_2_+β_24_) / √se(β_2_)^2^+se(β_24_)^2^

=(-0.002+0.008) / √0.0026^2^+0.0048^2^= 1.241

p-value=2*(1-NORMSDIST(1.241))= 0.214

◎p-value(Urine≧500 mL:Day1 vs Day3)

Z=(β_3_+β_34_) / √se(β_3_)^2^+se(β_34_)^2^

=(0.018+(-0.012)) / √0.015^2^+0.0158^2^= 0.313

p-value=2*(1-NORMSDIST(0.313))= 0.754

◎p-value Urine≧500 mL:Day2 vs Day3)

Z=〔 (β_2_+β_24_)-(β_3_+β_34_) 〕/ √〔 se(β_2_)+se(β_24_) 〕^2^+〔 se(β_3_)+se(β_34_) 〕^2^

=(-0.002+0.008)-( 0.018+(-0.012)) / √(0.0026+0.0048)^2^+(0.015+0.0158)^2^

= -0.004

p-value= NORMSDIST(-0.004)= 0.499

◎p-value(Urine＜500 mL:Day2 vs Day3)

Z=(β_2_-β_3_) / √se(β_2_)^2^+se(β_3_)^2^

=(-0.002-0.018) / √0.0026^2^+0.015^2^= -1.321

p-value=NORMSDIST(-1.321)= 1.813

*[HbT]*

| *[HbT]* | | p-value | Z |
| --- | --- | --- | --- |
| group difference at | Day1 | 0.043 |  |
|  | Day2 | 0.042 | 2.032 |
|  | Day3 | 0.168 | 1.379 |
| Urine≧500 mL | Day1 vs Day2 | 0.806 | 0.246 |
|  | Day1 vs Day3 | 0.827 | 0.218 |
|  | Day2 vs Day3 | 0.475 | -0.062 |
| Urine＜500 mL | Day1 vs Day2 | 0.695 |  |
|  | Day1 vs Day3 | 0.840 |  |
|  | Day2 vs Day3 | 0.366 | -0.343 |
| Urine≧500 mL vs Urine＜500 mL | | 0.043 |  |

| **Tests of Model Effects** | | | |
| --- | --- | --- | --- |
| Source | Type III | | |
|  | Wald Chi-Square | df | Sig. |
| (Intercept) | 16017.485 | 1 | .000 |
| day | .231 | 2 | .891 |
| day012UO total average | 3.433 | 1 | .064 |
| day * day012UO total average | .364 | 2 | .834 |
| Dependent Variable: *[HbT]*  Model: (Intercept), day, day012UO total average, day * day012UO total average | | | |

| **Parameter Estimates** | | | | | | | |
| --- | --- | --- | --- | --- | --- | --- | --- |
| Parameter | B | Std. Error | 95% Wald Confidence Interval | | Hypothesis Test | | |
|  |  |  | Lower | Upper | Wald Chi-Square | df | Sig. |
| (Intercept) | .414 | .0048 | .405 | .424 | 7385.008 | 1 | .000 |
| [day=3] | .001 | .0060 | -.011 | .013 | .041 | 1 | .840 |
| [day=2] | -.001 | .0027 | -.006 | .004 | .153 | 1 | .695 |
| [day=1] | 0^a^ | . | . | . | . | . | . |
| [day012UO total average =1] | .011 | .0056 | .000 | .022 | 4.093 | 1 | .043 |
| [day012UO total average =0] | 0^a^ | . | . | . | . | . | . |
| [day=3] * [day012UO total average =1] | .001 | .0067 | -.012 | .014 | .013 | 1 | .910 |
| [day=3] * [day012UO total average =0] | 0^a^ | . | . | . | . | . | . |
| [day=2] * [day012UO total average =1] | .002 | .0035 | -.005 | .009 | .364 | 1 | .546 |
| [day=2] * [day012UO total average =0] | 0^a^ | . | . | . | . | . | . |
| [day=1] * [day012UO total average =1] | 0^a^ | . | . | . | . | . | . |
| [day=1] * [day012UO total average =0] | 0^a^ | . | . | . | . | . | . |
| (Scale) | .001 |  |  |  |  |  |  |
| Dependent Variable: *[HbT]*  Model: (Intercept), day, day012UO total average, day * day012UO total average | | | | | | | |
| a. Set to zero because this parameter is redundant. | | | | | | | |

◎p-value(Day2)

Z=(β_4_+β_24_)/ √se(β_4_)^2^+se(β_24_)^2^

=0.011+0.002 / √0.0056^2^+0.0035^2^= 2.032

p-value=2*(1-NORMSDIST(2.032))= 0.042

◎p-value(Day3)

Z=(β_4_+β_34_) / √se(β_4_)^2^+se(β_34_)^2^

=0.011+0.001 / √0.0056^2^+0.0067^2^= 1.379

p-value=2*(1-NORMSDIST(1.379))= 0.168

◎p-value(Urine≧500 mL:Day1 vs Day2)

Z=(β_2_+β_24_) / √se(β_2_)^2^+se(β_24_)^2^

=(-0.001+0.002) / √0.0027^2^+0.0035^2^= 0.246

p-value=2*(1-NORMSDIST(0.246))= 0.806

◎p-value(Urine≧500 mL:Day1 vs Day3)

Z=(β_3_+β_34_) / √se(β_3_)^2^+se(β_34_)^2^

=(0.001+0.001) / √0.006^2^+0.0067^2^= 0.218

p-value=2*(1-NORMSDIST(0.218))= 0.827

◎p-value Urine≧500 mL:Day2 vs Day3)

Z=〔 (β_2_+β_24_)-(β_3_+β_34_) 〕/ √〔 se(β_2_)+se(β_24_) 〕^2^+〔 se(β_3_)+se(β_34_) 〕^2^

=(-0.001+0.002)-( 0.001+0.001) / √(0.0027+0.0035)^2^+(0.006+0.0067)^2^

= -0.062

p-value= NORMSDIST(-0.062)= 0.475

◎p-value(Urine＜500 mL:Day2 vs Day3)

Z=(β_2_-β_3_) / √se(β_2_)^2^+se(β_3_)^2^

=(-0.00104-0.00122))/ √0.0027^2^+0.006^2^= -0.343

p-value=NORMSDIST(-0.343)= 0.366

*StO_2_* (%)

| *StO_2_* | | p-value | Z |
| --- | --- | --- | --- |
| group difference at | Day1 | 0.264 |  |
|  | Day2 | 0.171 | -0.949 |
|  | Day3 | 0.232 | 1.195 |
| Urine≧500 mL | Day1 vs Day2 | 0.096 | -1.302 |
|  | Day1 vs Day3 | 0.380 | -0.307 |
|  | Day2 vs Day3 | 0.492 | -0.020 |
| Urine＜500 mL | Day1 vs Day2 | 0.543 |  |
|  | Day1 vs Day3 | 0.136 |  |
|  | Day2 vs Day3 | 0.115 | 1.577 |
| Urine≧500 mL vs Urine＜500 mL | | 0.264 |  |

| **Tests of Model Effects** | | | |
| --- | --- | --- | --- |
| Source | Type III | | |
|  | Wald Chi-Square | df | Sig. |
| (Intercept) | 7605.568 | 1 | .000 |
| day | 4.190 | 2 | .123 |
| day012UO total average | .904 | 1 | .342 |
| day * day012UO total average | 5.028 | 2 | .081 |
| Dependent Variable: Tissue hemoglobin oxygen saturation (%)  Model: (Intercept), day, day012UO total average, day * day012UO total average | | | |

| **Parameter Estimates** | | | | | | | |
| --- | --- | --- | --- | --- | --- | --- | --- |
| Parameter | B | Std. Error | 95% Wald Confidence Interval | | Hypothesis Test | | |
|  |  |  | Lower | Upper | Wald Chi-Square | df | Sig. |
| (Intercept) | 46.803 | .4845 | 45.854 | 47.753 | 9330.949 | 1 | .000 |
| [day=3] | -3.925 | 2.6320 | -9.084 | 1.233 | 2.224 | 1 | .136 |
| [day=2] | .295 | .4848 | -.655 | 1.245 | .371 | 1 | .543 |
| [day=1] | 0^a^ | . | . | . | . | . | . |
| [day012UO total average =1] | .612 | .5476 | -.461 | 1.685 | 1.250 | 1 | .264 |
| [day012UO total average =0] | 0^a^ | . | . | . | . | . | . |
| [day=3] * [day012UO total average =1] | 2.755 | 2.7638 | -2.661 | 8.172 | .994 | 1 | .319 |
| [day=3] * [day012UO total average =0] | 0^a^ | . | . | . | . | . | . |
| [day=2] * [day012UO total average =1] | -1.577 | .8572 | -3.257 | .103 | 3.385 | 1 | .066 |
| [day=2] * [day012UO total average =0] | 0^a^ | . | . | . | . | . | . |
| [day=1] * [day012UO total average =1] | 0^a^ | . | . | . | . | . | . |
| [day=1] * [day012UO total average =0] | 0^a^ | . | . | . | . | . | . |
| (Scale) | 25.030 |  |  |  |  |  |  |
| Dependent Variable: Tissue hemoglobin oxygen saturation (%)  Model: (Intercept), day, day012UO total average, day * day012UO total average | | | | | | | |
| a. Set to zero because this parameter is redundant. | | | | | | | |

◎p-value(Day2)

Z=(β_4_+β_24_)/ √se(β_4_)^2^+se(β_24_)^2^

=(0.612+(-1.577)) / √0.5476^2^+0.8572^2^= -0.949

p-value=NORMSDIST(-0.949)= 0.171

◎p-value(Day3)

Z=(β_4_+β_34_)/ √se(β_4_)^2^+se(β_34_)^2^

=(0.612+2.755)/ √0.5476^2^+2.7638^2^= 1.195

p-value=2*(1-NORMSDIST(1.195))= 0.232

◎p-value(Urine≧500 mL:Day1 vs Day2)

Z=(β_2_+β_24_) / √se(β_2_)^2^+se(β_24_)^2^

=(0.295+(-1.577)) / √0.4848^2^+0.8572^2^= -1.302

p-value=NORMSDIST(-1.302)= 0.096

◎p-value(Urine≧500 mL:Day1 vs Day3)

Z=(β_3_+β_34_) / √se(β_3_)^2^+se(β_34_)^2^

=(-3.925+2.755) / √2.632^2^+2.7638^2^= -0.307

p-value=NORMSDIST(-0.307)= 0.380

◎p-value Urine≧500 mL:Day2 vs Day3)

Z=〔 (β_2_+β_24_)-(β_3_+β_34_) 〕/ √〔 se(β_2_)+se(β_24_) 〕^2^+〔 se(β_3_)+se(β_34_) 〕^2^

=(0.295+(-1.577))-( -3.925+2.755)/ √(0.4848+0.8572)^2^+(2.632+2.7638)^2^

= -0.020

p-value=NORMSDIST(-0.020)= 0.492

◎p-value(Urine＜500 mL:Day2 vs Day3)

Z=(β_2_-β_3_) / √se(β_2_)^2^+se(β_3_)^2^

=0.295-(-3.925) / √0.4848^2^+2.632^2^= 1.577

p-value=2*(1-NORMSDIST(1.577))= 0.115

*[H_2_O]*

| *[H_2_O]* | | p-value | Z |
| --- | --- | --- | --- |
| group difference at | Day1 | 0.747 |  |
|  | Day2 | 0.380 | 0.878 |
|  | Day3 | 0.061 | -1.550 |
| Urine≧500 mL | Day1 vs Day2 | 0.766 | 0.297 |
|  | Day1 vs Day3 | 0.466 | -0.085 |
|  | Day2 vs Day3 | 0.866 | 0.169 |
| Urine＜500 mL | Day1 vs Day2 | 0.550 |  |
|  | Day1 vs Day3 | 0.034 |  |
|  | Day2 vs Day3 | 0.018 | -2.106 |
| Urine≧500 mL vs Urine＜500 mL | | 0.747 |  |

| **Tests of Model Effects** | | | |
| --- | --- | --- | --- |
| Source | Type III | | |
|  | Wald Chi-Square | df | Sig. |
| (Intercept) | 667.938 | 1 | .000 |
| day | 5.030 | 2 | .081 |
| day012UO total average | .148 | 1 | .701 |
| day * day012UO total average | 10.054 | 2 | .007 |
| Dependent Variable: *[H_2_O]*  Model: (Intercept), day, day012UO total average, day * day012UO total average | | | |

| **Parameter Estimates** | | | | | | | |
| --- | --- | --- | --- | --- | --- | --- | --- |
| Parameter | B | Std. Error | 95% Wald Confidence Interval | | Hypothesis Test | | |
|  |  |  | Lower | Upper | Wald Chi-Square | df | Sig. |
| (Intercept) | 10.290 | .5720 | 9.169 | 11.411 | 323.642 | 1 | .000 |
| [day=3] | 2.273 | 1.0696 | .177 | 4.370 | 4.518 | 1 | .034 |
| [day=2] | -.419 | .7005 | -1.792 | .954 | .358 | 1 | .550 |
| [day=1] | 0^a^ | . | . | . | . | . | . |
| [day012UO total average =1] | .236 | .7316 | -1.198 | 1.670 | .104 | 1 | .747 |
| [day012UO total average =0] | 0^a^ | . | . | . | . | . | . |
| [day=3] * [day012UO total average =1] | -2.410 | 1.1965 | -4.755 | -.065 | 4.057 | 1 | .044 |
| [day=3] * [day012UO total average =0] | 0^a^ | . | . | . | . | . | . |
| [day=2] * [day012UO total average =1] | .744 | .8426 | -.907 | 2.396 | .780 | 1 | .377 |
| [day=2] * [day012UO total average =0] | 0^a^ | . | . | . | . | . | . |
| [day=1] * [day012UO total average =1] | 0^a^ | . | . | . | . | . | . |
| [day=1] * [day012UO total average =0] | 0^a^ | . | . | . | . | . | . |
| (Scale) | 11.402 |  |  |  |  |  |  |
| Dependent Variable: *[H_2_O]*  Model: (Intercept), day, day012UO total average, day * day012UO total average | | | | | | | |
| a. Set to zero because this parameter is redundant. | | | | | | | |

◎p-value(Day2)

Z=(β_4_+β_24_) / √se(β_4_)^2^+se(β_24_)^2^

=(0.236+0.744) / √0.7316^2^+0.8426^2^= 0.878

p-value=2*(1-NORMSDIST(0.878))= 0.380

◎p-value(Day3)

Z=(β_4_+β_34_) / √se(β_4_)^2^+se(β_34_)^2^

=(0.236+(-2.41)) / √0.7316^2^+1.1965^2^= -1.550

p-value=NORMSDIST(-1.550)= 0.061

◎p-value(Urine≧500 mL:Day1 vs Day2)

Z=(β_2_+β_24_) / √se(β_2_)^2^+se(β_24_)^2^

=(-0.419+0.744)/ √0.7005^2^+0.8426^2^= 0.297

p-value=2*(1-NORMSDIST(0.297))= 0.766

◎p-value(Urine≧500 mL:Day1 vs Day3)

Z=(β_3_+β_34_) / √se(β_3_)^2^+se(β_34_)^2^

=(2.273+(-2.41)) / √1.0696^2^+1.1965^2^= -0.085

p-value=NORMSDIST(-0.085)= 0.466

◎p-value Urine≧500 mL:Day2 vs Day3)

Z=〔 (β_2_+β_24_)-(β_3_+β_34_) 〕/ √〔 se(β_2_)+se(β_24_) 〕^2^+〔 se(β_3_)+se(β_34_) 〕^2^

=(-0.419+0.744)-( 2.273+(-2.41)) / √(0.7005+0.8426)^2^+(1.0696+1.1965)^2^

= 0.169

p-value=2*(1-NORMSDIST(0.169))= 0.866

◎p-value(Urine＜500 mL:Day2 vs Day3)

Z=(β_2_-β_3_) / √se(β_2_)^2^+se(β_3_)^2^

=(-0.419)- 2.273/ √0.7005^2^+1.0696^2^= -2.106

p-value=NORMSDIST(-2.106))= 0.018

**1.6 Detailed analyses and results for Figure 3**

**VEGF**

Oliguria

| **Correlations** | | |
| --- | --- | --- |
|  | | VEGF (pg/mL) |
| *[H_2_O]* | Correlation Coefficient | .532^**^ |
|  | Sig. (2-tailed) | .003 |
|  | N | 29 |
| *. Correlation is significant at the 0.05 level (2-tailed). | | |
| **. Correlation is significant at the 0.01 level (2-tailed). | | |

Non-oliguria

| **Correlations** | | |
| --- | --- | --- |
|  | | VEGF (pg/mL) |
| *[H_2_O]* | Correlation Coefficient | .304 |
|  | Sig. (2-tailed) | .076 |
|  | N | 35 |
| *. Correlation is significant at the 0.05 level (2-tailed). | | |
| **. Correlation is significant at the 0.01 level (2-tailed). | | |

**Albumin**

Oliguria

| **Correlations** | | |
| --- | --- | --- |
|  | | Albumin (g/dL) |
| *[H_2_O]* | Pearson Correlation | -.456^*^ |
|  | Sig. (2-tailed) | .025 |
|  | N | 24 |
| *. Correlation is significant at the 0.05 level (2-tailed). | | |
| **. Correlation is significant at the 0.01 level (2-tailed). | | |

Non-oliguria

| **Correlations** | | |
| --- | --- | --- |
|  | | Albumin (g/dL) |
| *[H_2_O]* | Pearson Correlation | -.281 |
|  | Sig. (2-tailed) | .102 |
|  | N | 35 |
| *. Correlation is significant at the 0.05 level (2-tailed). | | |
| **. Correlation is significant at the 0.01 level (2-tailed). | | |
